# A chromosome‐scale reference genome of trifoliate orange (*Poncirus trifoliata*) provides insights into disease resistance, cold tolerance and genome evolution in *Citrus*

**DOI:** 10.1111/tpj.14993

**Published:** 2020-10-18

**Authors:** Ze Peng, Jessen V. Bredeson, Guohong A. Wu, Shengqiang Shu, Nidhi Rawat, Dongliang Du, Saroj Parajuli, Qibin Yu, Qian You, Daniel S. Rokhsar, Frederick G. Gmitter, Zhanao Deng

**Affiliations:** ^1^ Department of Environmental Horticulture Gulf Coast Research and Education Center University of Florida IFAS 14625 County Road 672 Wimauma FL 33598 USA; ^2^ Molecular and Cell Biology Department University of California, Berkeley Berkeley CA 94720 USA; ^3^ US Department of Energy Joint Genome Institute Lawrence Berkeley National Lab 1 Cyclotron Road Berkeley CA 94720 USA; ^4^ Citrus Research and Education Center University of Florida, IFAS 700 Experiment Station Rd Lake Alfred FL 33850 USA

**Keywords:** citrus, cold tolerance, disease resistance, evolution, genome, Huanglongbing, *Poncirus*

## Abstract

Trifoliate orange (*Poncirus trifoliata*), a deciduous close relative of evergreen *Citrus*, has important traits for citrus production, including tolerance/resistance to citrus greening disease (Huanglongbing, HLB) and other major diseases, and cold tolerance. It has been one of the most important rootstocks, and one of the most valuable sources of resistance and tolerance genes for citrus. Here we present a high‐quality, chromosome‐scale genome assembly of *P. trifoliata*. The 264.9‐Mb assembly contains nine chromosomal pseudomolecules with 25 538 protein‐coding genes, covering 97.2% of the estimated gene space. Comparative analyses of *P. trifoliata* and nine *Citrus* genomes revealed 605 species‐specific genes and six rapidly evolving gene families in the *P. trifoliata* genome. *Poncirus trifoliata* has evolved specific adaptation in the *C‐repeat/DREB binding factor* (*CBF*)‐dependent and *CBF*‐independent cold signaling pathways to tolerate cold. We identified candidate genes within quantitative trait loci for HLB tolerance, and at the loci for resistance to citrus tristeza virus and citrus nematode. Genetic diversity analysis of *Poncirus* accessions and *Poncirus*/*Citrus* hybrids shows a narrow genetic base in the US germplasm collection, and points to the importance of collecting and preserving more natural genetic variation. Two phenotypically divergent *Poncirus* accessions are found to be clonally related, supporting a previous conjecture that dwarf Flying Dragon originated as a mutant of a non‐dwarfing type. The high‐quality genome reveals features and evolutionary insights of *Poncirus*, and it will serve as a valuable resource for genetic, genomic and molecular research and manipulation in citrus.

## Significance Statement

A high‐quality, chromosome‐scale reference genome of trifoliate orange has enabled a comprehensive comparative genomics analysis of *Citrus*‐related genomes, discovery of gene families under expansion or positive selection, identification of gene families associated with important disease resistance and cold tolerance, and localization of candidate genes for tolerance to Huanglongbing, the most destructive disease in citrus. This genome will be a valuable resource for genomic research and manipulation in citrus and related plants.

## INTRODUCTION

Citrus is one of the most economically important fruit crops grown in more than 114 countries (Talon and Gmitter, 2008). In 2017, the world citrus acreage was 9.3 million hectares, and citrus production was 146.6 million tons (FAO statistics, http://www.fao.org/faostat/en/). The most commonly cultivated citrus species include sweet orange (*Citrus* × *sinensis*), mandarin (*C. reticulata*), grapefruit (*C*. × *paradisi*), pummelo (*C. maxima*) and lemon (*C*. × *limon*). Citrus is mainly consumed as fresh fruit or juice, which provide abundant vitamin C and other phytochemicals for human nutrition. However, commercial production of citrus is confronted with many challenges from biotic and abiotic stresses. Currently, the most devastating disease in citrus is Huanglongbing (HLB) or citrus greening disease, which is presumably caused by the bacterium *Candidatus* Liberibacter asiaticus (*C*Las) transmitted by the Asian citrus psyllid (*Diaphorina citri*, ACP; Bové, 2006). HLB has caused tremendous yield loss in citrus throughout the world (Bové, 2006). In Florida alone, the estimated economic loss due to HLB was at least $4 billion during the period from 2012−2013 to 2015−2016 (Trejo‐pech *et al*., 2018). Other diseases that limit citrus productivity include several species of *Phytophthora* and nematodes, and citrus tristeza virus (CTV; Radhika *et al*., 2008; Kamiri *et al*., 2018). Additionally, catastrophic economic losses caused by cold (freezes) have been recorded in several regions of the world (Sahin‐Cevik and Moore, 2006). Therefore, improving disease resistance and cold tolerance of cultivated citrus has been a major objective of major citrus breeding programs in the world.

Trifoliate orange (*Poncirus trifoliata*, 2*n* = 2*x* = 18) is a close relative of *Citrus* species, both belonging to the Rutaceae family. It was considered the only species in *Poncirus* until the discovery of *Poncirus polyandra* in the 1980s (Ding *et al*., 1984; Pang *et al*., 2003). Native to China, *P. trifoliata* has become naturalized in other regions of Asia, USA, Australia and Europe (Nesom, 2014). *Poncirus trifoliata* is sexually compatible with *Citrus* species (Spiegel‐Roy and Goldschmidt, 1996), but distinct from *Citrus* in many characteristics. It is deciduous (versus evergreen in *Citrus*), has three leaflets on each leaf and large thorns on the shoots, and produces bitter, inedible fruit containing many seeds. *Poncirus trifoliata* and its hybrids have been widely utilized as rootstocks in citrus production due to several advantageous features, including cold hardiness and resistance/tolerance to several major diseases and pests (Boava *et al*., 2011; Gong and Liu, 2013). *Poncirus trifoliata* is resistant to CTV, the most important viral pathogen of citrus (Kitajima *et al*., 1964; Tanaka *et al*., 1971; Garnsey and Barrett, 1987; Gmitter *et al*., 1996), and citrus nematodes (*Tylenchulus semipenetrans*; Ducharme, 1948; Cameron *et al*., 1954; Ling *et al*., 2000), and highly tolerant to *Phytophthora* species causing citrus seedling damping‐off, root and foot rot, and gummosis (Graham, 1990; Yang *et al*., 2001; Kamiri *et al*., 2018; Tian *et al*., 2018). A single dominant locus (*Ctv*) has been identified for the CTV resistance in *P*. *trifoliata* (Gmitter *et al*., 1996), and genetically and physically mapped (Deng *et al*., 2001; Yang *et al*., 2003). *Poncirus trifoliata* can provide high levels of tolerance to HLB and confers resistance to ACP (George and Lapointe, 2019). *Poncirus trifoliata* has been a major source of valuable genes for use in citrus sexual hybridization and somatic hybridization. It is expected to be a very important source of valuable genes for using cisgenic approaches to improving *Citrus* toward enhanced resistance to important biotic and abiotic stresses. *Poncirus* *trifoliata* also has considerable medicinal values, with anti‐inflammatory, anti‐tumor and anti‐anaphylactic activities (Yi *et al*., 2004; Rahman *et al*., 2015).

Another important feature of *P. trifoliata* is its cold tolerance. *Poncirus trifoliata* is extremely cold‐hardy and can survive −26°C (Spiegel‐Roy and Goldschmidt, 1996). This capability was probably acquired during its evolution in a cold habitat in northeastern Asia (Oustric *et al*., 2017). Other than *P. trifoliata*, Ichang papeda (*Citrus ichangensis*) has been considered the most cold‐hardy among evergreen *Citrus* species (Gmitter and Hu, 1990). Kumquats (*Fortunella* spp.) are considered cold tolerant within the *Aurantioideae* sub‐family of the citrus family (Krueger and Navarro, 2007). On the other hand, *C. maxima* and *C. medica* are the most cold‐sensitive species in *Citrus* (Nordby and Yelenosky, 1982; Crifò *et al*., 2011). Therefore, a comparative analysis of these cold‐tolerant and cold‐sensitive genomes would yield insights into the evolutionary signatures leading to their differences in cold tolerance. Studies in *Arabidopsis* have shown that the *C‐repeat/DREB binding factor* (*CBF*) pathway plays an important role in plant tolerance to cold stresses (Shi *et al*., 2018). When plants are under cold environmental conditions, *CBF* genes are induced rapidly to express and then activate the downstream target cold‐regulated (*COR*) genes or *CBF* regulons (Gilmour *et al*., 1998; Liu *et al*., 1998; Shi *et al*., 2018). However, *CBFs* only regulate less than 20% of the *COR* genes (Park *et al*., 2015; Shi *et al*., 2015). Therefore, *CBF*‐independent pathways are also critical for cold signaling regulation (Park *et al*., 2015). In *P. trifoliata*, a few genes have been well characterized and involved in cold tolerance, including *bHLH* (Huang *et al*., 2013), *PRP* (Peng *et al*., 2015), *ICE1* (Huang *et al*., 2015) and *ERF109* (Wang *et al*., 2018b). A previous transcriptome study revealed differentially expressed *P. trifoliata* genes (DEGs) in response to cold stresses at three time points (6, 24 and 72 h) after cold treatment (Wang *et al*., 2015). Overall, the molecular mechanisms of cold tolerance in *P. trifoliata* remain poorly understood. Thus, more studies are needed to fully exploit this unusual and striking feature of *P. trifoliata*, which makes it a perfect model and valuable source of candidate genes for improving cold tolerance in citrus. With the potential to confer to citrus these many favorable traits, including resistance to several major diseases and cold tolerance, a reference genome of *P. trifoliata* would greatly expedite the genetic and genomic research and improvement of citrus.

In order to explore the unique features of *P. trifoliata*, we performed genome sequencing and *de novo* assembly, and obtained a high‐quality, chromosome‐scale reference genome for this important species. We investigated the gene family evolution and positive selection in *P. trifoliata* by performing a comparative genomic analysis of the *P. trifoliata* genome and publicly available genomes of *Citrus*. To further understand *Poncirus*, we obtained and analyzed the genome sequences of *P. polyandra*, the only other species in *Poncirus*, and a number of important accessions of *P. trifoliata* and its hybrid cultivars. Moreover, we identified candidate genes in *P*. *trifoliata* by using previously identified quantitative trait loci (QTLs) for HLB tolerance, and loci for resistance to CTV and citrus nematodes. The high‐quality genome assembly has uncovered features and evolutionary insights of *Poncirus*, and it will serve as a valuable resource for genetic, genomic and molecular research and manipulation of citrus.

## RESULTS

### Genome sequencing and assembly

**Figure 1:**
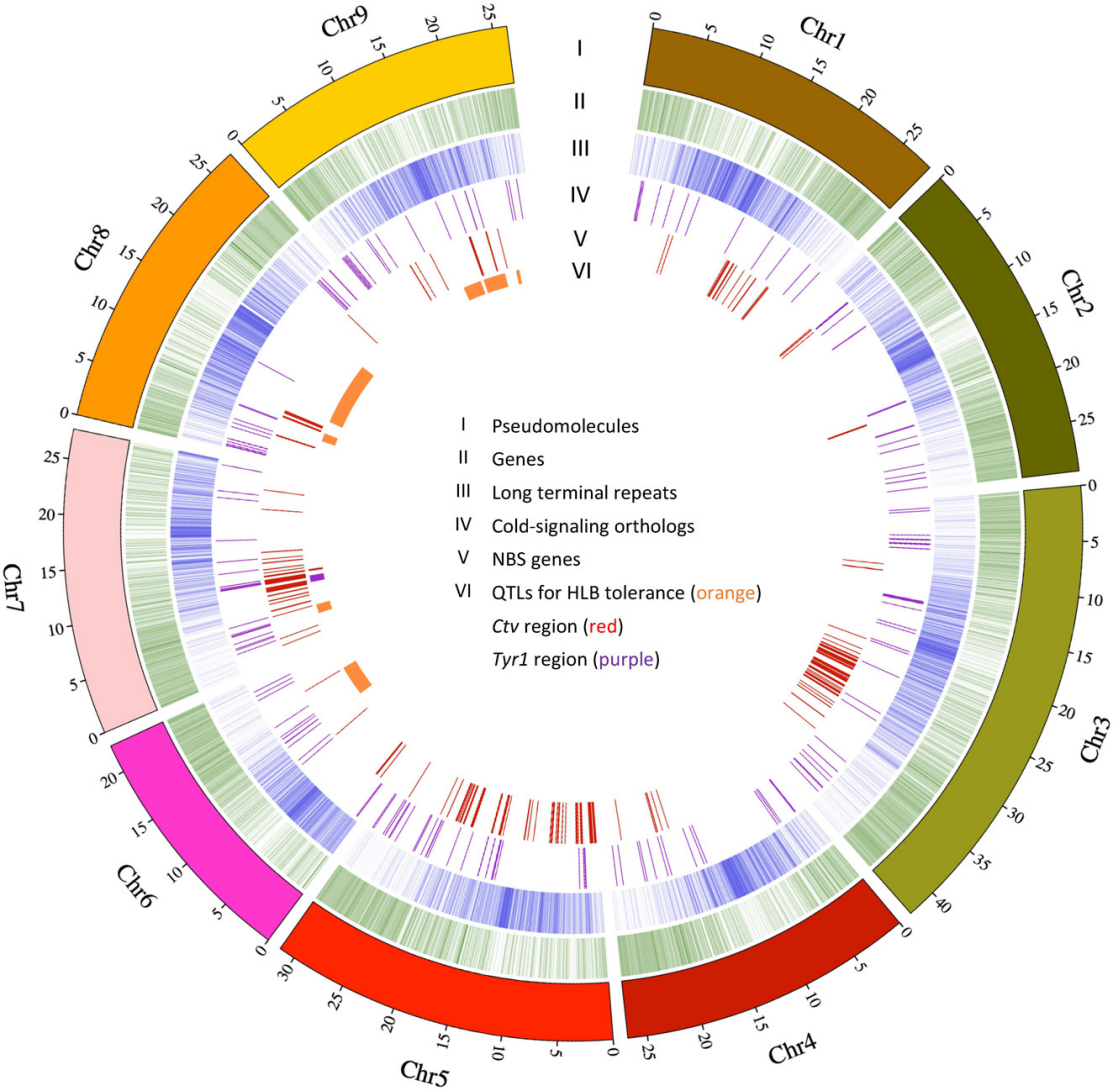
Characterization of the *Poncirus trifoliata* genome. I. The nine assembled pseudomolecules (in Mb) corresponding to the nine chromosomes (Chr1−Chr9) of *Citrus × clementina*. II. Locations of predicted gene models. III. Locations of predicted long terminal repeat (LTR) transposable elements (TEs). IV. Locations of predicted homologous cold signaling genes. V. Locations of predicted nucleotide‐binding site (NBS)‐containing genes. VI. Orange: locations of quantitative trait loci (QTLs) associated with Huanglongbing (HLB) tolerance and loci for resistance to citrus tristeza virus (*Ctv*) and citrus nematode (*Tyr1*). Red: *Ctv* region. Purple: *Tyr1* region.

The HLB‐tolerant *P. trifoliata* accession DPI 50‐7 was selected as the reference genotype for whole‐genome sequencing and assembly. We sequenced the genome of DPI 50‐7 *de novo* combining ~140 × PacBio single‐molecule long‐read sequence reads with ~80 × Illumina shotgun sequence (Table S1). To achieve chromosome‐scale scaffolding, we used Hi‐C chromosome conformation capture data (Figure S1). The nine chromosomal pseudomolecules were numbered and oriented according to the convention of *C. × clementina* (Wu *et al*., 2014; Figure S2). These sequences represent a non‐redundant mosaic of the two haplotypes of diploid *P. trifoliata*, representing more than 99% of the assembled contigs (Figure 1). The largest scaffold was 42.7 Mb (Chr3), and the overall N50 length was 27.7 Mb, corresponding to the median chromosome length (Table 1). The GC content was estimated to be 33.9%.

**Table 1:** Summary statistics of the *Poncirus trifoliata* genome assembly and annotation

Total size of genome assembly	264.9 Mb
Chromosome number (2*n*)	18
Number of chromosomal pseudomolecules	9
Number of scaffolds	152
Longest scaffold	42.7 Mb
Scaffold N50 length	27.7 Mb
Number of contigs	809
Contig N50 length	842.8 kb
GC content	33.9%
Number of gene models	25 538
Number of genes with isoforms	4398
Mean transcript length	1667 bp
Mean coding sequence length	1268 bp
Mean exon length	296 bp
Percentage of TEs	42.6%

TE, transposable element.

### Genome annotation

A large proportion (42.6%) of the *P. trifoliata* genome was comprised of transposable elements (TEs). Long terminal repeats (*LTRs*) were the most predominant repeats (24.62%), including *LTR/Gypsy* (9.42%) and *LTR/Copia* (6.37%; Table S2). Other types of repeats accounted for smaller proportions of the genome, including DNA transposons (10.40%), LINE (2.57%) and SINE (0.25%). An integrated strategy combining *ab initio*‐ and homology‐based methods was applied to predict gene models in the *P. trifoliata* genome. In total, we identified 25 538 protein‐coding genes with 33 230 transcripts. The transcripts for primary gene models had a mean length of 1667 bp, a mean coding sequence length of 1268 bp, and a mean exon length of 296 bp (Table 1). In addition, 4398 genes had spliced isoforms with an average of 2.7 isoforms per gene. To evaluate the completeness of our genome, the predicted protein sequences were compared with the 1440 eukaryotic genes in the BUSCO OrthoDB9 embryophyta dataset and obtained a completeness of 97.2%. The completeness of this genome is currently the highest compared with 10 other publicly available *Citrus* genomes (Table S3). The high completeness and N50 length support the quality of this genome assembly.

### Identification of cold signaling‐related genes, nucleotide‐binding site (NBS)‐leucine‐rich repeat (LRR) genes and transcription factors (TFs)

To identify cold signaling‐related genes in *Poncirus* and *Citrus*, we focused on homologs of *Arabidopsis* genes that are involved in cold signaling (Guo *et al*., 2018; Liu *et al*., 2018a; Shi *et al*., 2018) and *P. trifoliata* genes known to respond to cold stress. Most of the *Arabidopsis thaliana* cold signaling pathway genes and *P. trifoliata* cold tolerance‐related genes had homologs in *Citrus* species. Based on analysis using existing annotations of the 10 *Citrus*‐related genomes, the homologs of *A. thaliana* cold signaling pathway genes were assigned to 45 gene families containing 69 *P. trifoliata* genes (Figure 1; Table S4). In addition, there were 49 gene families containing either ‘first‐wave’ TF genes or *CBF* regulon genes in *A. thaliana*, in which 119 *P. trifoliata* genes were included. Several gene families containing cold signaling‐related genes from *A. thaliana* found no members in *Citrus*‐related species. This may be a consequence of comparing a perennial woody plant with an herbaceous annual plant, and that different genetic players have been adopted to confer cold tolerance in *Citrus* species compared with *A. thaliana*. After re‐assigning gene families using re‐annotated gene models, an average of ~88% of genes or gene families containing cold signaling‐related genes were recovered for the 10 genomes (Tables S4 and S5). If only considering low‐copy genes (less than two genes for each species), a higher percentage (~92%) of genes or gene families were recovered. Therefore, the vast majority of genes were not influenced (when it comes to finding the gene) by the bias of different annotation pipelines of genomes. By matching *P. trifoliata* genes from the current study to previously identified DEGs upon cold treatment in *P. trifoliata* (Wang *et al*., 2015), a total of 118, 467 and 1292 genes were found to be differentially expressed at 6, 24 and 72 h after cold treatment, respectively. The evolutionary dynamics of these candidate cold signaling‐related genes were further investigated in the following sections.

A total of 348 NBS genes was identified in the genome of *P. trifoliata* (Figure 1; Table S6). These NBS genes were further classified into different classes based on the presence of Toll/Interleukin‐1 receptor (TIR), coiled coil (CC) and LRR domains. The two largest classes with at least one of these domains were the ‘CC‐NBS’ (84; 24.1%) and ‘CC‐NBS‐LRR’ (77; 22.1%) types. A small proportion (58; 16.7%) contained the TIR domain compared with other domains. Through phylogenetic analysis, these NBS genes could be classified into three major groups (Figure S3). The TF analysis revealed a total of 1493 putative TF genes in 58 families, representing 5.8% of the predicted gene models in the *P. trifoliata* genome (Table S7). The most predominant TF family in *P. trifoliata* is the bHLH family (124), followed by NAC (123), ERF (110) and MYB (109) families.

### **Gene family analysis and evolutionary dynamics among** ****Citrus**** **‐related species**

**Figure 2:**
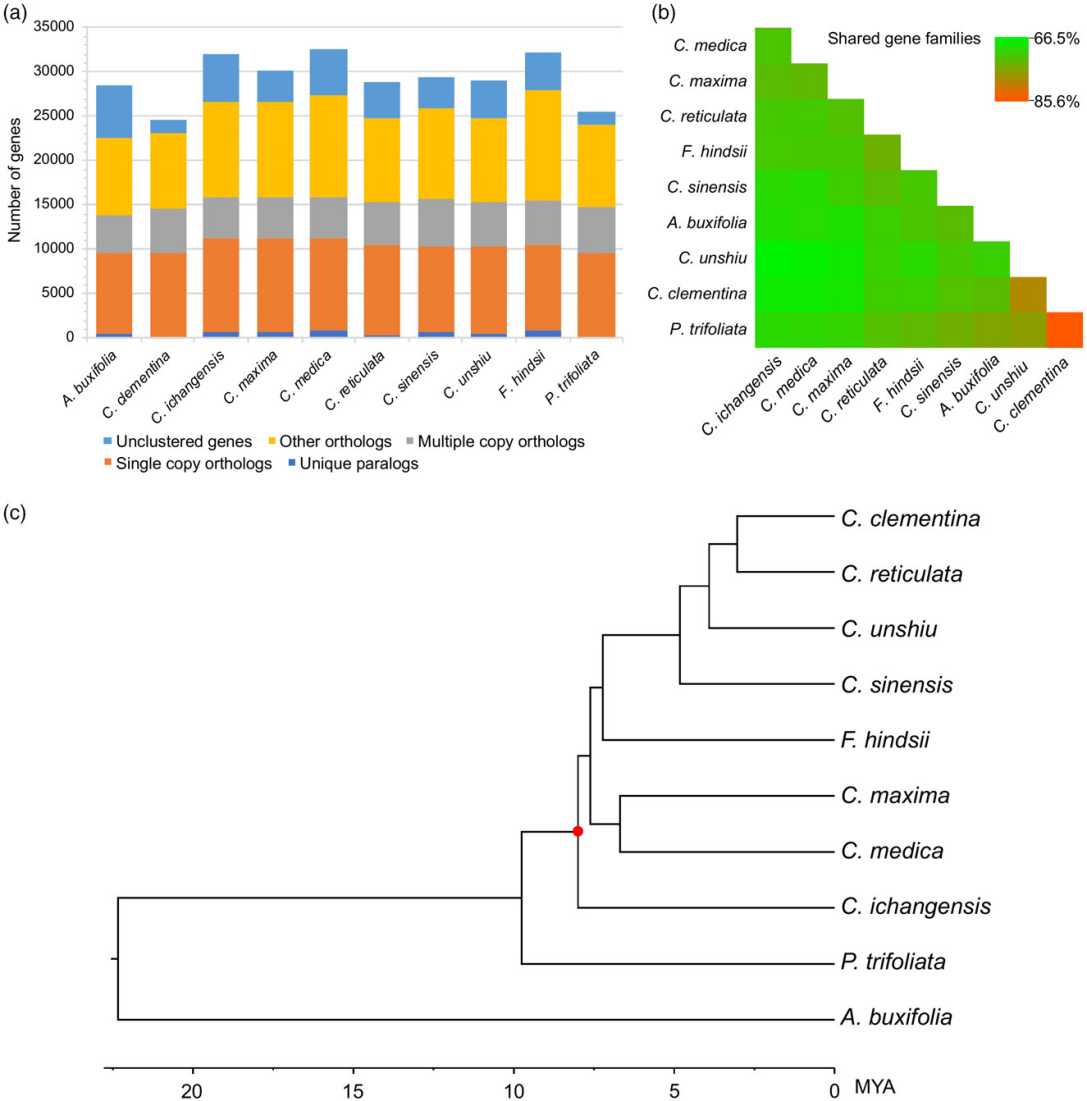
Comparative genomics of the 10 *Citrus* and *Citrus*‐related species analyzed in this study using existing annotations. (a) Gene numbers for different types of orthologous/paralogous gene family relationships in 10 species. Detailed classification of orthologous/paralogous types is provided in Table S5 (column ‘Classification’). (b) A heatmap for comparison of shared gene families for each pair of species. (c) A phylogenetic tree containing 10 analyzed species. Note that some citrus, e.g. *Citrus* × *sinensis*, arose as the offspring of previously admixed individuals, and thus divergence times between admixtures are not meaningful for these so‐called species.

The gene family assignment using existing annotations was first explored to have a global view of gene families among the 10 genomes. A total of 253 788 (86.7%) genes from the 10 species, including *P. trifoliata* and nine other *Citrus* and *Citrus*‐related species, were assigned to 28 226 gene families, among which 15 763 (55.8%; 98 960 genes) represented one‐to‐one orthologous gene families (Figure 2a; Table S8). There were 11 597 (41.1%) gene families shared by all 10 species, which implied their conservation. In *P. trifoliata*, 9467 (37.1%) genes were single‐copy orthologs, 208 genes were assigned to 67 gene families specific to *P. trifoliata*, while 1529 genes were unclustered (Figure 2a; Table S9). Among these genes, analysis using re‐annotations supported that 605 genes are specific to *P. trifoliata*. These exonerate recoverable genes are not present in other genomes under the same annotation method. By calculating the percentage of shared gene families relative to the total (unique) gene families of each pair of species, *P. trifoliata* shared a higher proportion of its gene families with *C. × clementina* (Figure 2b). To explore the phylogenetic relationship of the 10 *Citrus*‐related species, a high‐confidence phylogenetic tree was constructed using the protein sequences of 5747 single‐copy orthologs (Figure 2c). The species tree topology obtained from the coalescence method was the same as that of the concatenation method. The phylogenetic tree revealed that *P. trifoliata* diverged from the common ancestor of *Citrus* clade about 9.8 million years ago (MYA). This result is close to previous estimations on *Citrus−Poncirus* divergence time (4.0–9.6 MYA in Pfeil and Crisp, 2008; 9.1–9.2 MYA in Wu *et al*., 2018). Within the *Citrus* clade, *C. ichangensis* is located at the basal position, while *C. maxima* (pummelo) and *C. medica* (citron) form a distinct cluster. *Fortunella hindsii* (kumquat) is located at a basal position relative to *C. × clementina* (Clementine), *C. reticulata* (mandarin), *C. unshiu* (Satsuma) and *C. × sinensis* (sweet orange). It is noteworthy that the cold‐tolerant species, *P. trifoliata*, *C. ichangensis* and *F. hindsii* all seem to be located at a more basal position than other cold‐sensitive species. The re‐annotation and analysis of single‐copy gene families showed a high agreement between existing annotations and re‐annotations (Table S10). The same species tree topology was obtained after re‐constructing the tree using exonerate models. Therefore, the phylogenetic tree obtained from existing annotations was used for further analysis.

Through gene family expansion and contraction analysis using existing annotations, in *P. trifoliata* there were 45 rapid evolving gene families that were significantly expanded (41) or contracted (4) across the species tree with a family‐wide *P* ≤ 0.01 and branch‐specific *P* ≤ 0.01 (Table S11). We performed further filtering on these gene families through re‐analysis using re‐annotated gene models. An expanded family was considered supported by re‐annotation if it met the following thresholds: (i) at least half of the *P. trifoliata* genes from this family were recovered by re‐annotation and the exonerate models were assigned to the same family; (ii) the re‐analysis using exonerate models also showed a family‐wide *P* ≤ 0.01 and branch‐specific *P* ≤ 0.01. No significantly contracted families were supported by re‐annotation. We finally identified six significantly expanded gene families for *P. trifoliata*, which were supported by both existing annotations and re‐annotations (Table S11). To further investigate the gene functions, gene ontology (GO) enrichment analysis was performed on the 605 genes specific to *P. trifoliata*, and on the six rapid evolving gene families in *P. trifoliata*, respectively (Table S12). The structural constituents of cell wall (GO:0005199) and plant‐type cell wall organization (GO:0009664) were significantly enriched for genes specific to *P. trifoliata*. Three GO terms were significantly enriched for the rapid evolving genes, including protein binding (GO:0005515), DNA binding (GO:0003677) and protein dimerization activity (GO:0046983). Specifically, among the six rapid evolving gene families, one family (OG1.5_1006) encodes a group of LRR proteins, whose only homolog in *Arabidopsis* (AT2G34930) encodes a disease resistance family protein involved in defense response. Interestingly, two gene families were associated with TEs, including a gag protein family associated with retrotransposon (OG1.5_1264) and a Tam3‐transposase family associated with DNA transposon (OG1.5_1413). Another family was a group of zinc finger proteins with hAT dimerization domain. The remaining two families had unknown functions.

As shown in Table S4, most of the gene families containing cold signaling‐related genes had similar gene numbers across all *Citrus*‐related species, showing no significant differential gene gain/loss between cold‐hardy and other *Citrus* species, including cold‐sensitive *C. maxima*, *C. medica* and *C. × sinensis*. However, a larger number of genes was observed in cold‐tolerant *P. trifoliata* and *F. hindsii* for an amino acid transporter family (OG1.5_1258) containing a DEG responsive to cold stress in *P. trifoliata*. This gene family contains fewer gene members in the cold‐sensitive *Citrus* species. Interestingly, for an FAR1 TF family (OG1.5_1201) containing two DEGs responsive to cold stress, a much larger number of genes was observed in all three cold‐tolerant species than in the cold‐sensitive *Citrus* species. A similar trend was observed for gene assignment using re‐annotated gene models. These *P. trifoliata* genes may potentially play an important role in increasing its cold tolerance.

### **Whole‐genome duplication (WGD) and divergence between the two** ****Poncirus****  **species**

To investigate the evolutionary history of the *P. trifoliata* genome and other *Citrus* genomes, the *Ks* was estimated for paralogous gene groups in each species. A clear secondary peak (*Ks* = ~1.5) was identified for *P. trifoliata*, similar with *C. × clementina*, *C. × sinensis*, as well as with other *Citrus*‐related genomes (Figures S4a and S5a–g). Similar results have been observed previously in *C*.* × clementina* (*Ks* peak = 1.5; Wu *et* *al*., 2014) and *C*.* × sinensis* (*Ks* mean = 1.27; Xu *et al*., 2013). Thus, as expected, we confirm that the *Citrus−Poncirus* lineage shared an ancient WGD event, which is also shared with other eudicots. To study the divergence between *Poncirus* species, we predicted a total of 8326 gene models for *P*. *polyandra* based on the existing re‐sequencing data, and calculated the *Ks* values between these genes and their *P*. *trifoliata* orthologs. We observed a clear peak at *Ks* = 0.007 (Figure S4b). By assuming the neutral substitution rate *r* to be 1 ~ 2 × 10^−9^ (Wu *et al*., 2014), the divergence time between *P*. *trifoliata* and *P*. *polyandra* was estimated to be approximately 1.75 ~ 3.5 MYA. Among the 8326 genes identified from *P*. *polyandra*, 2170 match to the 5747 single‐copy orthologs among 10 *Citrus*‐related species. If we include *P*. *polyandra* gene models when constructing the *Poncirus−Citrus* phylogenetic tree (using 2170 single‐copy orthologs including *P*. *polyandra*), the estimated divergence time between *P*. *trifoliata* and *P*. *polyandra* is about 2.82 MYA, which falls into the range of the *Ks*‐based method (Figure 2c). These results indicate that from an evolutionary perspective the *P*. *polyandra/P*. *trifoliata* split is one of the most recent divergences among the 11 *Citrus*‐related species.

### Positive selection (dN/dS analysis)

**Figure 3:**
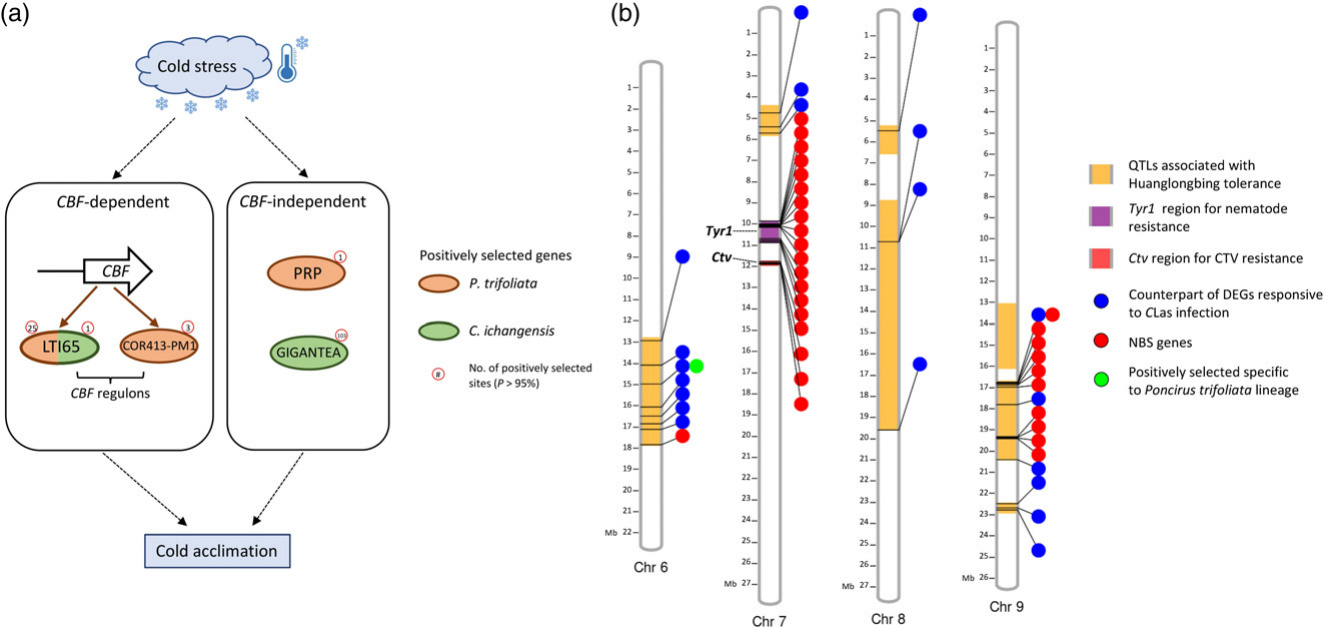
Candidate genes associated with cold tolerance and disease resistance. (a) Positively selected genes associated with cold signaling pathways and specific to two cold‐tolerant species. Genes in brown ovals were specifically positively selected in the *Poncirus trifoliata* lineage. Genes in green ovals were specifically positively selected in the *Citrus ichangensis* lineage. (b) A graphic map showing quantitative trait loci (QTL) and candidate genes in the *P. trifoliata* genome associated with tolerance to the Huanglongbing (HLB) disease, as well as candidate genes associated with citrus tristeza virus (CTV) and nematode resistance within *Ctv* and *Tyr1* regions.

To gain more insight into adaptive evolution in the *P*. *trifoliata* genome, positive selection was first tested for the 5747 single‐copy orthologs from *P*. *trifoliata* and nine *Citrus* species using a branch‐site model. Our analysis revealed that 35 genes underwent positive selection specifically in the *P*. *trifoliata* lineage, which was supported by both existing annotations and re‐annotations (Table S13; ‘Category 4’). To further compare the positively selected genes among the three cold‐tolerant species, additional analyses were performed using the three cold‐sensitive species as the background branch (Table S13; ‘Category 1–3’). Similar with *C*. *ichangensis*, *P*. *trifoliata* has evolved particular adaptation in both the *CBF*‐dependent and *CBF*‐independent cold signaling pathways to tolerate the cold environment from a more northern and temperate natural habitat (Figure 3a). Four genes (shown in Figure 3a) from *P*. *trifoliata* and *C*. *ichangensis* were supported by both existing annotations and re‐annotations. Strikingly, the homolog of a *CBF* regulon, *LOW‐TEMPERATURE‐INDUCED 65* (*LTI65*), was positively selected in two cold‐tolerant species *P*. *trifoliata* and *C*. *ichangensis* (Figure 3a). However, *P*. *trifoliata* has a larger number of positively selected sites (25) than *C*. *ichangensis* (1). We further noticed *LTI65* remains a positively selected gene when treating the nine species as the background branch and *P*. *trifoliata* as the foreground branch. Therefore, *P*. *trifoliata*, the most cold‐hardy species among the 10, underwent extensive and lineage‐specific adaptations in *LTI65*, a player in the *CBF*‐dependent cold signaling pathway. The other two positively selected genes in *P*. *trifoliata* were *COR413‐PM1*, another *CBF* regulon, and *hybrid proline‐rich protein* (*PRP*). Importantly, the *PRP* gene has already been characterized in *P*. *trifoliata* previously and confirmed to confer cold tolerance (Peng *et al*., 2015). Additionally, three DEGs responsive to cold stress were also positively selected uniquely in the *P*. *trifoliata* genome, which was supported by both existing annotations and re‐annotations (Table S13; ‘Category 4’). These species‐unique adaptations together with the unique expansions of genes responsive to cold stress may explain why *P*. *trifoliata* is the most cold‐tolerant species among the 10 species analyzed.

### Candidate genes associated with HLB tolerance and resistance to CTV and citrus nematodes

To further exploit the HLB tolerance feature of *P*. *trifoliata*, we mapped the markers flanking 14 QTL intervals associated with HLB tolerance that were previously placed on linkage maps of *P*. *trifoliata* (Huang *et al*., 2018). Their genomic locations were found in the *P*. *trifoliata* genome with a physical size ranging from 0.24 Mb (QTL: FS‐2016‐t8) to 10.61 Mb (QTL: CD‐2015‐t8; Table S14). Some of the QTLs are overlapping; after overlapping QTLs were merged, seven unique genomic regions for the 14 QTLs were observed (highlighted in Figure 3b). By searching the *P*. *trifoliata* counterparts (reciprocal best hits) of DEGs responsive to *C*Las infection within these QTL regions, we identified 20 *P*. *trifoliata* genes that are counterparts of previously identified DEGs potentially associated with HLB (Figure 3b; Table S15; Fu *et al*., 2016; Hu *et al*., 2017; Yu *et al*., 2017). Interestingly, a TF WRKY70 (orange1.1g020291m/Ptrif.0006s1042) was upregulated upon *C*Las infection in sweet orange and positively selected uniquely in *P*. *trifoliata* (considering nine species as the background branch). The positive selection of this gene was also supported by re‐annotations. Considering the evidence, this TF gene may play an important role in *P*. *trifoliata* tolerance to HLB. Strikingly, 11 NBS genes (putative disease resistance genes) were found in these QTL regions, and 10 of them (as two clusters) are located in a single QTL FS‐2015‐t9a (phenotypic variation explained 24.5%; physical size 3.75 Mb) on Chromosome 9 (Table S16). Moreover, one of them (Ptrif.0009s1449) is a counterpart of a DEG (orange1.1t00706.1) responsive to *C*Las infection. Overall, these genes can serve as candidate genes for further investigation of the genetic factors conferring HLB tolerance in *P*. *trifoliata*.

By mapping the *Ctv* locus controlling CTV resistance and *Tyr1* locus controlling nematode resistance to the *P*. *trifoliata* genome, candidate disease resistance genes (NBS genes) within these regions were identified (Figure 3b; Table S16). Strikingly, there was a total of 15 NBS genes (as two clusters) located within the *Tyr1* region (1 Mb in size). Overall, these major loci controlling disease resistance in *P*. *trifoliata* have clusters of NBS genes. Chromosomes 7 and 9 of *P*. *trifoliata* may play a very important role in conferring resistance/tolerance to HLB, CTV and citrus nematodes.

### **Genetic diversity and relatedness of** ****Poncirus****  **and hybrids**

**Figure 4:**
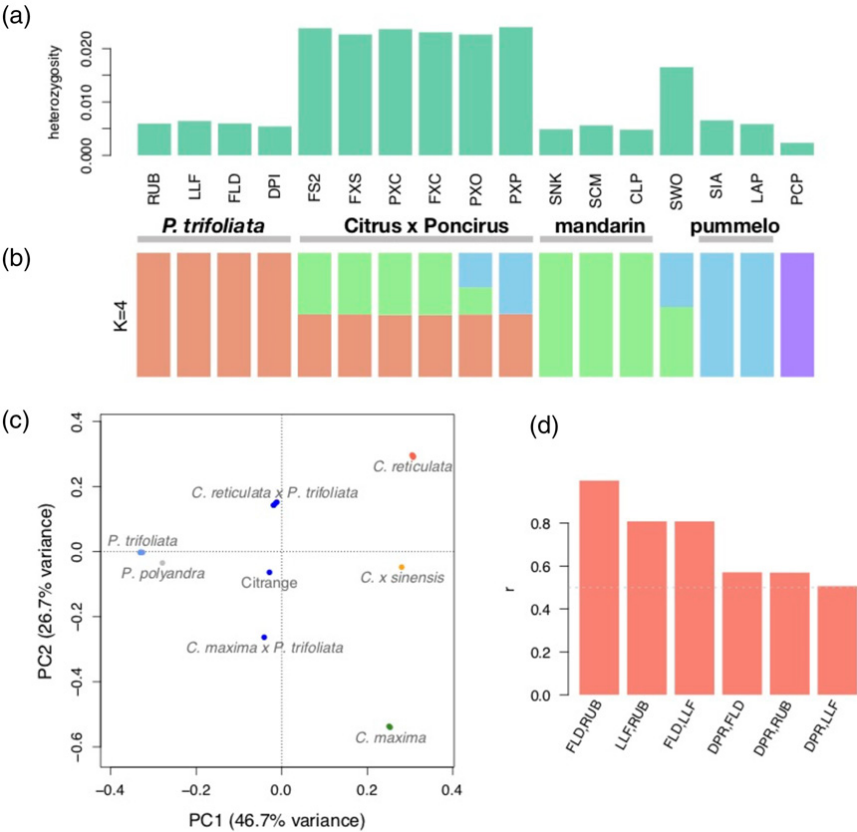
Genetic diversity and relatedness of *Poncirus* and hybrids. (a) Heterozygosity of five *Poncirus* accessions and six *Citrus* accessions, including *Poncirus trifoliata* (RUB = Rubidoux, LLF = Little Leaf, FLD = Flying Dragon, and DPI = DPI 50‐7), *Poncirus polyandra* (PCP), *Citrus reticulata* (CLP = Cleopatra, SNK = Sunki, and SCM = Sun Chun Sha Kat), *Citrus maxima* (LAP = low acid and SIA = Siamese) and *Citrus sinensis* (SWO), and six *Poncirus*/*Citrus* hybrids [PXO = Carrizo citrange (*C*. × *sinensis* × *P*. *trifoliata*), PXP = *Citrus maxima* × *P. trifoliata*, and four *C*. *reticulata*/*Poncirus* hybrids FXC, PXC, FXS, FS2]. Accession IDs are provided in Table S1. (b) Admixture analysis at k = 4 reveals the genetic makeup of the re‐sequenced accessions in terms of four species. Each bar represents the ancestry proportion of one accession, and the accessions are ordered as in panel. Note that *Poncirus polyandra* (PCP) represents a genetic isolate. (c) Principle component analysis (PCA) shows a population structure that is consistent with admixture analysis. (d) Genetic relatedness among the four *P. trifoliata* accessions. The coefficient of relatedness is shown for each pair of accessions. In particular, FLD and RUB are clonally related (*r* = 1), and all pairs show high degrees of relatedness (*r* ≥ 0.5).

All four *P*. *trifoliata* accessions (DPI 50‐7, Flying Dragon, Little Leaf and Rubidoux) have genome‐wide average heterozygosity at 0.5–0.6% (Figure 4a), similar to that observed in most pure *Citrus* species (Wu *et al*., 2018). By contrast, the heterozygosity of the *P*. *polyandra* accession is much lower (~0.2%). The *Poncirus/Citrus* hybrids are characterized by inter‐specific/generic sequence divergence with heterozygosity 2.3–2.4% (Figure 4a, where the three‐letter codes for the 17 accessions are also defined). Admixture analysis using 194 598 nuclear genic single nucleotide polymorphisms (SNPs) revealed the genetic composition of the 17 re‐sequenced accessions. At k = 3, the three source populations correspond to *Poncirus*, *C*. *maxima* (pummelos) and *C*. *reticulata* (mandarins). With k = 4, *P*. *polyandra* (PCP) separates from *P*. *trifoliata*, with the six *Poncirus*/*Citrus* hybrids showing no genetic contribution from *P*. *polyandra* (Figure 4b). These six hybrids exhibit three patterns of admixture, with four being mandarin/*Poncirus* hybrids, one as pummelo/*Poncirus* hybrid (PXP), and the Carrizo citrange (PXO) being consistent with its parentage *C*.* × sinensis* × *P*. *trifoliata*. The sweet orange (SWO, *C*.* × sinensis*) is an admixture of *C*. *maxima* and *C*. *reticulata* with a complex origin (Figure 4b; Wu *et al*., 2014). Note that the ADMIXTURE software did not reveal the small amount of pummelo admixture in *C*. *reticulata* cultivars Cleopatra (CLP) and Sunki (SNK) as identified with local ancestry analysis (Wu *et al*., 2018). Principle component analysis with the same set of nuclear genic SNPs revealed a population structure that is consistent with admixture analysis (Figure 4c). In particular, PC1 separates *Poncirus* from *Citrus* with *Poncirus*/*Citrus* hybrids at intermediate positions. PC2 separates pummelos from mandarins with sweet orange situated between the two parental species (Figure 4c). *Poncirus polyandra* splits from the other accessions with PC3.

Genetic relatedness among the *P. trifoliata* accessions and *Poncirus*/*Citrus* hybrids are computed following the method of Wu *et al*. (2018). The four *P. trifoliata* accessions are genetically related to each other with coefficient of relatedness > 0.5 (Figure 4d). Throughout the genome, each *Poncirus* pair shares either one or both haplotypes (Figure S6). In particular, Flying Dragon and Rubidoux are related by somatic mutations with *r* = 1, though their phenotypes are quite different, including the dwarfing trait of Flying Dragon (Cheng and Roose, 1995). This clonal relatedness is in line with an earlier proposal that Flying Dragon originated as a mutant of a non‐dwarfing genotype (Cheng and Roose, 1995). We also note that a previously published *Poncirus* accession (*P. trifoliata* Pomeroy; Wu *et al*., 2018) is clonally related to Little Leaf. The high degree of relatedness among the four *P. trifoliata* accessions indicates a very narrow genetic base for the *Poncirus* accessions in the US germplasm, and points to the importance of exploring additional genetic variation that exists in the native habitat of *Poncirus* (Zhu *et al*., 2015).

Among the six *Poncirus*/*Citrus* hybrids, genome‐wide haplotype sharing analysis verified the parentage of Carrizo citrange with *C. × sinensis* as the maternal parent and *P. trifoliata* Flying Dragon/Rubidoux (or its somatic mutant) being the paternal parent. The pummelo/*Poncirus* hybrid (PXP) has *P. trifoliata* Little Leaf as the paternal parent, with the maternal parent being an unknown *C. maxima* that is different from the Siamese pummelos LAP and SIA. Of the four mandarin/*Poncirus* hybrids, FXC = Cleopatra mandarin × *P. trifoliata* Flying Dragon, PXC = *P. trifoliata* Little Leaf × Cleopatra mandarin, FXS = unknown mandarin × *P. trifoliata* Little Leaf, and FS2 = unknown mandarin × *P. trifoliata* Flying Dragon, where the maternal parents are listed first, we did not distinguish between somatic mutants in listing the parentage (e.g. FLD and RUB). By ‘unknown mandarin’ in the parents of FXS and FS2, we meant that the parents cannot be found in the three mandarins (CLP, SNK, SCM) of our analysis.

## DISCUSSION

The availability of a high‐quality reference genome is a prerequisite for effective and efficient mining of a crop genome. As a major rootstock in the citrus industry and a major source of resistance genes for citrus breeding, *P. trifoliata* has many desirable traits, including resistance to several major diseases, especially tolerance to HLB, and cold tolerance. Since more than a century ago, many of these traits have been targeted for introducing into citrus for crop improvement. However, a *P*. *trifoliata* genome assembly was not available until now. Our study filled this gap by providing a high‐quality and chromosome‐scale reference genome of *P. trifoliata* to the citrus research community. This genome should lay a solid foundation for future genetic, genomic and molecular studies in this important species.

### ****De novo**** **assembly and TE**

Assembling heterozygous genomes *de novo* has been a challenge (Kajitani *et al*., 2014). High contents of repetitive sequences often present a major barrier to reliable assembly of such genomes from short‐read sequences. We applied an integrated assembly strategy combining Illumina short‐read sequencing, PacBio long‐read sequencing and Hi‐C scaffolding technology to facilitate the assembly process and improve the accuracy. Our assembly strategy has proven to be effective. The Hi‐C data and resulting scaffolding improved the assembly N50 length by approximately 32‐fold. To our knowledge, this genome is the fourth reference genome assembly at chromosome‐scale or pseudomolecule‐level in *Citrus*‐related genera or, more broadly speaking, in the family *Rutaceae*, following *C. × sinensis* (Xu *et al*., 2013), *C. × clementina* (Wu *et al*., 2014) and *C. maxima* (Wang *et al*., 2018a). The completeness of this genome is 97.2% based on the BUSCO score, which is higher than that of previous reports, such as *C. reticulata* (96%; Wang *et al*., 2018a) and *F. hindsii* (95.1%; Zhu *et al*., 2019). A considerable proportion of the *P. trifoliata* genome is comprised of TEs (42.63%), and the most abundant repeat types are LTR retrotransposons (*Copia* and *Gypsy* most frequent). This seems a shared feature among *Citrus*‐related genomes.

### Re‐annotation of genes of interest

One limitation of using existing gene model annotations of other citrus genomes for comparative analysis is that some findings could be potentially caused by the different annotation pipelines. Therefore, we first used existing annotations to gain a global view of gene family assignment and identified a preliminary set of genes or gene families of interest, including homologs of cold signaling‐related genes, rapid evolving gene families, *P. trifoliata*‐specific genes and single‐copy genes across 10 genomes. To address and minimize potential biases from different annotation pipelines, we therefore applied a homology‐based gene annotation pipeline (Blast/Exonerate) to all the genomic regions potentially containing the preliminary set of genes of interest for all the 10 genomes, including *P. trifoliata* itself. Findings from existing annotations were further filtered according to the re‐analysis using the re‐annotated gene models (under the same pipeline).

### Phylogenetic analysis

A phylogenetic tree is needed to understand the evolutionary relationships among species. The use of highly conserved and low‐copy nuclear genes in resolving phylogenetic relationships has long been recognized (Duarte *et al*., 2010; Zhang *et al*., 2012). Previously, as the genome of *P. trifoliata* was unavailable, a phylogenetic tree was constructed by using SNPs located within low‐copy genes for nine *Citrus*‐related species, including *P. trifoliata* (Zhu *et al*., 2019). With the availability of 10 *Citrus*‐related genomes including this *P. trifoliata* genome, we were able to use protein sequences of 5747 conserved and single‐copy nuclear genes (genome‐wide) across the 10 species to re‐construct a highly confident species tree. The species tree topology was supported by both the concatenation method and the coalescence method after using either existing annotations or re‐annotations. To test the effect of gene numbers on tree structure for the concatenation method, we also constructed additional trees by using a subset of these single‐copy genes, including using 100, 1000, 2000, 3000 and 4000 randomly selected genes. It was found that when using more than 2000 genes, the general tree structure became stable and remained unchanged even if more genes were included. Therefore, this species tree can be considered relatively confident and stable. In this analysis, *P. trifoliata* showed the closest relationship to *C. ichangensis* among the analyzed species, which is consistent with a previous report (Wu *et al*., 2018). The *P. trifoliata* genome will be useful for phylogenetic studies in citrus.

### ****Poncirus polyandra****

*Poncirus polyandra* is the only existing species within *Poncirus* other than *P. trifoliata*. Almost extinct in the wild, *P. polyandra* has been classified as a protected wild plant (national second‐class) in China (http://rep.iplant.cn/protlist). Recently, the chloroplast genome of *P. polyandra* was sequenced and its 105 predicted gene models were used for phylogenetic analysis (Li *et al*., 2019; Yang *et al*., 2019). In this study, the genome sequences of *P. polyandra* were reported, and a pipeline combining reference‐based assembly using RGAAT and gene prediction using Genewise was applied for gene model predictions. We obtained 8326 high‐quality predicted protein‐coding genes in *P. polyandra*. This pipeline can also be applied to other species with a reference genome available from a close relative. On the basis of these 8326 genes, we proposed a divergence time of 2.82 MYA between *P. trifoliata* and *P. polyandra*. Our study provided additional genomic resources to this rare and endangered species, which can be useful for future research of *P. polyandra* and may provide guidance to develop conservation strategies.

### **What’s unique about the genome of** ****Poncirus trifoliata**** **?**

Our analysis using existing annotations revealed that 11 597 gene families were shared by the 10 analyzed *Poncirus* and *Citrus* species, indicating that these gene families may have conserved functions and represent the essence of *Citrus*‐related species. However, there were 605 genes in *P. trifoliata* that are not shared with the remaining nine species. These *P. trifoliata*‐specific genes are likely not required for the basic growth and development of *Citrus* species, but they may represent the diversity or uniqueness of *P. trifoliata*. Notably, the GO enrichment analysis revealed that two cell‐wall‐related GO terms were significantly enriched in *P. trifoliata*‐specific genes, which may indicate a unique cell wall structure and organization in *P. trifoliata*. LRR proteins play an important role in plant innate immunity against pathogens, and most immune receptors contain the LRR domain for pathogen recognition (Padmanabhan *et al*., 2009). While the NBS‐LRR genes are the most common type of disease resistance genes in plants (McHale *et al*., 2006), interestingly, we identified a rapid evolving gene family in *P. trifoliata* comprised of non‐NBS type LRR genes. The expansion of this family in the *P. trifoliata* genome may have a potential role in its disease resistance as their single homolog in *Arabidopsis* belongs to a disease resistance gene family. TEs provide an abundant source of genetic variations, thereby contributing to the evolution and genetic diversity of plants (Kidwell and Lisch, 1997; Butelli *et al*., 2012; Borredá *et al*., 2019). In *P*. *trifoliata*, we identified two significantly expanded gene families encoding the retrotransposon gag protein and Tam3‐transposase. Interestingly, in contrast to most TEs whose regulations are epigenetic dependent, the activity of Tam3 is controlled by its own Tam3‐transposase as characterized in *Antirrhinum majus* (Zhou *et al*., 2017). Moreover, the transposition of Tam3 is closely controlled by temperature, with more transposition activities at lower temperatures and repressed at higher temperatures (Hashida et al., 2003, 2006). Therefore, it is likely that the cold habitat of *P*. *trifoliata* is associated with the expansion of Tam3‐transposase gene families. Overall, the enriched functions for lineage‐specific genes and rapid evolving genes in *P*. *trifoliata* may be essential for its adaptive evolution, and contribute to the characters specific to this species.

### **Adaptive evolution of** ****Poncirus trifoliata****  **and** ****Citrus**** 

As the most cold‐hardy species among all *Citrus*‐related species (Spiegel‐Roy and Goldschmidt, 1996), the *P*. *trifoliata* genome provides a valuable candidate gene pool to improve cold tolerance in citrus. Our analysis revealed specific adaptions potentially associated with cold tolerance not only in *P*. *trifoliata*, but also in the other well‐known cold‐tolerant species, *C*. *ichangensis*. Our analysis indicated that *P*. *trifoliata* and *C*. *ichangensis* had adaptations in both the *CBF*‐independent and *CBF*‐dependent pathways. One positively selected gene homologous to *LTI65* was shared by *P*. *trifoliata* and *C*. *ichangensis*. Therefore, *LTI65* could be important in regulating cold signaling as it was positively selected in two cold‐tolerant species. These positively selected genes can be good candidates for further studies and genetic engineering to improve cold tolerance in citrus. Apart from the above adaptations, the DEGs responsive to cold and positively selected specifically in *P*. *trifoliata* branch may reveal genetic factors and mechanisms of cold tolerance in *P*. *trifoliata*.

### Tolerance to HLB, CTV and citrus nematode

A recent study identified QTLs for HLB tolerance in *P. trifoliata* (Huang *et al*., 2018), based on linkage maps of *P. trifoliata* and reference genomes of *C. × clementina* and *C. × sinensis*. Because the *P*. *trifoliata* genome was not available then, the study was not able to associate the QTLs with *P. trifoliata* genes or genomic sequences. With the availability of this *P. trifoliata* genome, candidate genes located within these QTLs could be mined. To be considered as a candidate gene controlling HLB tolerance, it would be reasonable to assume that its expression likely will change after infection with *C*Las. Therefore, we catalogued *P*. *trifoliata* genes that are counterparts of DEGs responsive to *C*Las infection based on three previous transcriptome studies. The WRKY70 TF gene under positive selection on Chromosome 6 and QTL FS‐2015‐t9a harboring two NBS gene clusters on Chromosome 9 may be promising candidates for further studies. Similarly, NBS gene clusters were also observed within *Ctv* and *Tyr1* regions on Chromosome 7. The NBS genes identified in this study may facilitate understanding the disease resistance in *Poncirus* and expedite introgression of resistance genes into *Citrus*.

### **Need to preserve the genetic diversity in** ****Poncirus**** 

*Poncirus* is extremely important for current citrus breeding and future genetic improvement of citrus. Our study reveals a surprisingly narrow genetic base in the US germplasm collection, and indicates a strong need for further exploration and preservation of its natural genetic variation. A previous study (Zhu *et al*., 2015) indicates a wide genetic diversity in the native habitat of *P*. *trifoliata* in China. While still in existence, preserving and sharing this natural variation should be considered as an important item on the agenda of citrus germplasm researchers in the world to prevent the tragedy that had happened to *P*. *polyandra*, the only other species in *Poncirus*.

### Conclusion

In summary, our study significantly enriched the genomic resources for *P*. *trifoliata*, an extremely important relative of *Citrus* with remarkable disease resistance and cold tolerance. Our reference genome and comprehensive evolutionary analysis are expected to contribute to a better understanding of disease resistance, cold tolerance and other unique biological features of *P*. *trifoliata*, and play an important role in breeding, genetic, genomic and molecular research of *Citrus*.

## EXPERIMENTAL PROCEDURES

### Plant materials

*Poncirus trifoliata* accessions DPI 50‐7, Flying Dragon, Little Leaf and Rubidoux, six *P*. *trifoliata/Citrus* hybrids, including Carrizo citrange, US‐802, US‐812, US‐897 and US‐942, and XCitroncirus X639, and one *P*. *polyandra* accession were sequenced (Table S1). *Poncirus trifoliata* DPI 50‐7 is an accession maintained by Florida Department of Plant Industry (DPI; https://www.fdacs.gov/Divisions‐Offices/Plant‐Industry). This accession has been used in various studies (Graham *et al*., 2003; Albiach‐Marti *et al*., 2004). Based on genotyping data (F.G. Gmitter Jr.), its heterozygosity was lower than some other *Poncirus* accessions and it has a high percentage of nucellar seedlings that develop from nucellar embryos, a type of apomixis that is important for *P*. *trifoliata* to be used as citrus rootstock. According to the most recent 2018–2019 report from Florida Department of Agriculture and Consumer Services Bureau of Citrus Budwood Registration (2019; https://www.fdacs.gov/Divisions‐Offices/Plant‐Industry/Bureaus‐and‐Services/Citrus‐Budwood‐Registration), *Poncirus* accessions and *Poncirus* hybrids were used as the rootstock for 3 224 857 citrus trees in Florida in 2018–2019. *Poncirus* and *Poncirus* hybrid rootstocks accounted for 82.2% of the top 20 rootstocks used in the 2018–2019 citrus propagation cycle (a total of 3 918 440 citrus plants budded on the top 20 rootstocks). Six *Poncirus* hybrids were included in this study, including Carrizo citrange, US‐802, US‐812, US‐897, US‐942 (Castle *et al*., 2009; Bowman *et al*., 2016) and XCitroncirus X639 (Table S1). Nucellar embryony is widespread in *Citrus* and *Poncirus* (Wang *et al*., 2017). It is very important and valuable for rootstock cultivars, because it allows for production of highly uniform plants (because of origin from the nucellar tissue) from seeds for use as rootstock for grafting (Castle *et al*., 2009). On the other hand, this may have led to high levels of relatedness among *Poncirus* accessions in germplasm collections. The selected and preserved accessions may have some differences in morphology and have different names, but might have originated from nucellar seedlings and have little to minor differences at the DNA level or in the genome. Young leaf tissues were collected from the above accessions and immediately put into liquid nitrogen for subsequent DNA extraction using the CTAB method (Cheng *et al*., 2003).

### Genome sequencing and assembly

The genome of *P*. *trifoliata* accession DPI 50‐7 was sequenced using PacBio, Illumina HiSeq 2500 (125 bp paired‐end reads) and Hi‐C technology. High‐molecular‐weight DNA was extracted from approximately 30 g of young leaves of *P*. *trifoliata* DPI 50‐7 seedlings as described by Kim *et al*. (2014) and sent to the University of Florida Interdisciplinary Center for Biotechnology Research (UF/ICBR; Gainesville, FL, USA), where library construction and SMRT sequencing were performed. A total of 10 µg of DNA was sheared in a Covaris g‐tube and converted to a sequencing library using the PacBio SMRT‐bell template kit following the manufacturer’s instructions (20 kb template preparation using BluePippin size selection) with a low threshold of 15 kb. A total of two continuous long‐read (CLR) libraries were made and sequenced. A total of 41 SMRT cells were run on an RS II sequencer using the P6‐C4 chemistry and a 4−6‐h movie length. Illumina whole‐genome shotgun sequencing was performed by Novogene Corporation (Beijing, China) for *P*. *trifoliata* accessions DPI 50‐7, Flying Dragon, Little Leaf and Rubidoux, *P*. *polyandra* and *P*. *trifoliata* hybrids using the Illumina HiSeq 2500 platform (125‐bp paired‐end reads). The standard Illumina sequencing protocol was followed for short‐insert paired‐end libraries. PacBio sequencing yielded 4 953 819 polymerase reads with an N50 length of 10 874 bp and a total of 37.1 Gb polymerase read bases (approximately 140 × coverage) for DPI 50‐7. A total of 21.2 Gb of raw short‐read sequences (approximately 80 × coverage) was obtained for DPI 50‐7, and 8.6–12.4 Gb of raw short‐read sequences (approximately 32–46 × coverage) was obtained for each of the other *P*. *trifoliata* accessions and hybrids (Table S1).

The *Poncirus trifoliata* DPI 50‐7 genome was assembled using ~140 × depth of sequencing coverage in PacBio CLR data as input to Falcon (Chin *et al*., 2016; v2018.03.12‐04.00) using the following parameters: genome_size=300000000 input_type=raw length_cutoff=5000 length_cutoff_pr=5000 pa_HPCdaligner_option=[‐v ‐B20 ‐t16 ‐k14 ‐e.70 ‐l3000 ‐s100] ovlp_HPCdaligner_option=[‐v ‐B20 ‐t32 ‐k25 ‐w5 ‐h70 ‐e.94 ‐l3000 ‐s100] pa_DBsplit_option=[‐x500 ‐s350] ovlp_DBsplit_option=[‐x500 ‐s350] falcon_sense_option=[‐‐output_multi ‐‐min_idt 0.70 ‐‐min_cov 2 ‐‐min_cov_aln 4 ‐‐max_cov_aln 30 ‐‐n_core 4] overlap_filtering_setting=[‐‐max_diff 38 ‐‐min_cov 3 ‐‐max_cov 58 ‐‐bestn 10 ‐‐min_len 5000]. Short‐insert paired‐end Illumina sequencing reads from DPI 50‐7 were aligned to the primary contigs with BWA‐MEM (Li, 2013; v0.7.17‐r1188) and filtered for properly‐paired primary alignments using SAMtools (Li *et al*., 2009; v1.6), then the median depth of each contig was calculated using BEDtools (Quinlan and Hall, 2010; v2.27.0). Primary contigs with median depth approximately half the expected depth (or lower) were aligned to all other primary contigs with minimap2 (Li, 2018; v2.5‐284‐g1739a26). Contigs with alignments at 90% similarity and aligned over 90% of their lengths were deemed redundant and set aside as alternate haplotigs. The remaining contigs were then scaffolded using the Dovetail Genomics HiRise Hi‐C‐based scaffolder. The assembly was curated for correctness and residual redundancy in JuiceBox (Durand *et al*., 2016a; Dudchenko *et al*., 2018; v1.9.0) with the JuiceBox Assembly Tools using Hi‐C alignments performed with Juicer (Durand *et al*., 2016b; v1.5.6‐64‐g94ec691). PacBio long reads were aligned to the scaffolds and alternate haplotigs with BLASR (Chaisson and Tesler, 2012) via pbalign (smrtlink v2.3.0, patch 5; parameters: ‐‐nproc 8 ‐‐algorithm blasr ‐‐maxHits 1 ‐‐algorithmOptions “‐useQuality ‐minReadLength 5000 ‐minSubreadLength 5000”) and the scaffolds polished using the Arrow (Chin *et al*., 2013) polishing algorithm (parameters: ‐‐diploid ‐‐noEvidenceConsensusCall lowercasereference ‐‐refineDinucleotideRepeats). A total of two long‐read signal polishing iterations were performed. The DPI 50‐7 short‐insert paired reads were aligned to the scaffolds and filtered (as described above), variants were called with FreeBayes (Garrison and Marth, 2012; v1.3.1‐17‐gaa2ace8; parameters: ‐‐genotype‐qualities ‐‐use‐mapping‐quality ‐‐report‐genotype‐likelihood‐max ‐‐standard‐filters ‐‐haplotype‐length 2 ‐‐strict‐vcf), and the resulting variants selected for supporting depth within the range of 10–135 ×, which were then used to patch small‐scale errors using a custom script (ILEC v0.2.0‐28‐g190e036; https://bitbucket.org/rokhsar‐lab/map4cns). This short‐read‐based polishing process was performed twice to yield the intermediate v1.2 assembly, and a total of six times to produce the final v1.3 assembly. The final polished scaffolds (v1.3) were then ordered, oriented and named to correspond to that of the *Citrus × clementina* (v1.0) assembly (downloaded from Phytozome).

### Genome annotation

A *de novo* TE library was constructed and annotated by RepeatModeler (Smit and Hubley, 2010). For LTR retrotransposons, we applied LTR_finder (Xu and Wang, 2007) and LTR_harvest (Ellinghaus *et al*., 2008) to identify candidates in the *P*. *trifoliata* genome. Outputs were processed using LTR_retriever (Ou and Jiang, 2018). The unknown predicted TEs were further classified using TEClass (Abrusán *et al*., 2009). The above predicted TEs were integrated into RepBase (Bao *et al*., 2015). Finally, RepeatMasker (Tarailo‐Graovac and Chen, 2009) was used for TE annotation in *P*. *trifoliata* genome assembly.

For gene model annotation, transcript assemblies for DPI 50‐7 were first generated from ~39 million pairs of 125‐bp (paired‐end) Illumina RNA‐seq reads using PERTRAN (Shu *et al*., 2013). A total of 46 423 transcript assemblies was generated using PASA (Haas *et al*., 2003) from above RNA‐seq transcript assemblies. Protein‐coding gene loci were determined based on transcript assembly alignments and/or EXONERATE alignments of protein sequences from Arabidopsis, poplar, cotton, Clementine mandarin, citrus, Kitaake rice, green foxtail, grape and Swiss‐Prot proteomes, aligned to the repeat‐soft‐masked *P. trifoliata* genome using RepeatMasker. Alignment footprints were extended up to 2 kb sequences in both directions unless extending into another locus on the same strand. Gene models were predicted by the homology‐based predictors, FGENESH+ (Salamov and Solovyev, 2000), FGENESH_EST, GenomeScan (Yeh *et al*., 2001), PASA assembly open reading frames (ORFs), and from AUGUSTUS via BRAKER1 (Hoff *et al*., 2015). For each locus, the best scored predictions were selected considering several positive factors (EST and protein support) and one negative factor (overlapping with repeats). PASA was used to further improve the selected gene predictions, including adding UTRs, splicing correction and adding alternative transcripts. A protein homology analysis was performed by comparing the PASA‐improved gene model proteins with the above‐mentioned proteomes to obtain the Cscore and protein coverage. The selected gene models were subject to Pfam analysis. The gene models whose protein sequences matched more than 30% of the Pfam TE domains were filtered out.

### Comprehensive search for cold signaling pathway and cold signaling‐related genes

For the analysis of cold signaling genes in *P. trifoliata*, the *CBF*‐dependent and *CBF*‐independent cold signaling pathway genes characterized in *A. thaliana* were summarized from three most recent reports (Guo *et al*., 2018; Liu *et al*., 2018a; Shi *et al*., 2018). The protein sequences of a total of 66 non‐redundant genes from the three reports were downloaded from NCBI (https://www.ncbi.nlm.nih.gov) or UniProt (https://www.uniprot.org/). In addition, 44 *CBF* regulon genes contributing to freezing tolerance and 27 cold‐induced ‘first‐wave’ TF genes that are induced in parallel with *CBF* genes in *A. thaliana* were also included in the analysis (Park *et al*., 2015, 2018). The homologs of the above genes in *Citrus*‐related genomes were determined using the Blast/OrthoMCL method as described in gene family analysis below. A previous transcriptome study revealed *P. trifoliata* DEGs in response to cold stress at three time points (6, 24 and 72 h) after cold treatment (Wang *et al*., 2015). To find *P. trifoliata* genes corresponding to the expressed sequence tag (EST) assemblies in that report, the EST sequences were compared with the transcript sequences of *P. trifoliata* gene models using BLAST. To ensure finding the correct corresponding genes, a high cutoff of alignment was used: (i) best hit from BLAST; (ii) [(alignment length × percentage of identity)/(query or subject length)] ≥ 99%.

### Analysis of NBS‐LRR genes

To identify NBS‐containing genes in the *P. trifoliata* genome, the protein sequences were first scanned using the NBS Hidden Markov Model (HMM) profile (PF00931) and ‘hmmsearch’ in Hmmer under E‐value 1e‐04 (Finn *et al*., 2011). In addition, a *P. trifoliata*‐specific NBS domain HMM profile was made by using the high‐quality hits from ‘hmmsearch’ (E‐value 1e‐60) and ‘hmmbuild’, which was also used to scan the protein sequences. The presence of NBS domain was further confirmed using PfamScan (Finn *et al*., 2016). The TIR and LRR domains were identified using the NCBI conserved domain tool. The CC domain was identified using Marcoil (probability > 90%; Delorenzi and Speed, 2002). To construct a phylogenetic tree for NBS genes, the NBS domain sequences for those with a complete NBS domain were included for tree construction using RAxML same as below. The phylogenetic tree was visualized using MEGA7.

### TF gene analysis

To identify genes encoding TFs in *P. trifoliata*, the protein sequences of predicted gene models were searched against the Plant Transcription Factor Database v5.0 (http://planttfdb.cbi.pku.edu.cn).

### Candidate gene identification within regions associated with disease resistance

Our previous report of QTLs associated with HLB tolerance (Huang *et al*., 2018) was based on linkage maps of *P. trifoliata* and either *C. clementina* or *C. sinensis* as the reference genomes. With the availability of this *P. trifoliata* reference genome, we mapped the markers flanking the QTL intervals directly to their own *P. trifoliata* reference genome. To search for candidate genes associated with *C*Las infection and HLB tolerance, three transcriptome studies reporting DEGs responsive to *C*Las infection in *C. sinensis*, *C. jambhiri* (rough lemon) and/or *C. hystrix* (Kaffir lime) were utilized (Fu *et al*., 2016; Hu *et al*., 2017; Yu *et al*., 2017). To find the counterpart genes of these DEGs in *P. trifoliata*, a combination of Reciprocal Best Hit and OrthoMCL (same as below) methods were applied. Thus, a *P. trifoliata* gene was considered as the counterpart if it is the reciprocal best hit of and orthologous to a DEG responsive to *C*Las infection. Other genes were also searched within the QTL regions, including NBS genes and positively selected genes specific to the *P. trifoliata* lineage among the 10 species described above. The two major loci associated with CTV (*Ctv*) and nematode resistance (*Tyr1*) were also mapped to the genome for candidate gene identification (Ling *et al*., 2000; Yang *et al*., 2003).

### Gene family analysis

The protein sequences of collected cold signaling‐related genes from above and gene models from *C. clementina* (v1.0), *A. thaliana TAIR10* (downloaded at Phytozome), *Citrus* × *sinensis* (v2), *C. ichangensis* (v1.0), *C. reticulata* (v1.0), *C. maxima* (v1.0), *C. medica* (v1.0), *Atalantia buxifolia* (v1.0), *Fortunella hindsii* (v1.0; downloaded from http://citrus.hzau.edu.cn/orange/download/index.php), *C. unshiu* (http://www.citrusgenome.jp) and *P. trifoliata* (from this study) were compared using all‐against‐all BLAST (*E*‐value 1e‐5). For gene models with multiple isoforms, protein sequences corresponding to the longest ORF were selected for further analysis. Homologous/orthologous gene families were assigned using OrthoMCL (inflation value 1.5; Fischer *et al*., 2011). The gene families were compared between the three cold‐hardy species (*P. trifoliata*, *C. ichangensis* and *F. hindsii*) and three cold‐sensitive *Citrus* species, including *C. maxima*, *C. medica* (Nordby and Yelenosky, 1982; Crifò *et al*., 2011) and *C*. × *sinensis*. The functions of genes were retrieved from the genome annotation files. GO enrichment analysis was performed using FatiGO software (http://babelomics.org).

### Phylogenetic analysis

A total of 5747 single‐copy gene families (one‐to‐one) across all 10 *Citrus*‐related species were used to construct a phylogenetic tree. The species tree topology was constructed using two methods, including a concatenation method and a coalescence method. For the concatenation method, the protein sequences for each gene family were first aligned using MAFFT (Katoh *et al*., 2005). Then, alignments were trimmed using Gblocks (Talavera and Castresana, 2007). The resulting sequence alignments were concatenated into a supermatrix to construct a species tree using RAxML with 1000 bootstraps (Stamatakis, 2014). The best protein substitution model ‘PROTGAMMAJTTF’ was selected using a perl script ProteinModelSelection.pl (https://cme.h‐its.org/exelixis/web/software/raxml/). *Atalantia buxifolia* was treated as the outgroup. The evolution time was calibrated using r8s by fixing the *Citrus* root age at 8 MYA (Wu *et al*., 2018). The phylogenetic tree was visualized using MEGA7 (Kumar *et al*., 2016). In addition, a coalescence method was applied using the same set of single‐copy gene families to compare the tree topologies with that of the concatenation method. Firstly, individual gene trees were constructed for each of the single‐copy gene families using the same method described for the concatenation method (using RAxML under the ‘PROTGAMMAJTTF’ model with 1000 bootstraps). The best‐scoring maximum likelihood (ML) gene trees were further analyzed using ASTRAL (v5.7.3; Zhang *et al*., 2018) to estimate a species tree under a multi‐species coalescent model with default parameters.

### Gene family evolution analysis

CAFÉ was used to investigate gene family expansion and contraction across all branches of the species tree (De Bie *et al*., 2006). Only gene families containing at least one gene in no less than two species were included for the analysis. We also excluded from this analysis gene families containing more than 100 genes for one or more species. CAFÉ was run with ‘‐s’ option to estimate the gene birth‐death parameter lambda and *P*‐value cutoff 0.01.

### Positive selection analysis

We first performed a positive selection analysis using the single‐copy gene families across all 10 *Citrus*‐related species (Table S13; ‘Category 4’). The protein alignments were converted to their corresponding CDS alignments. The *P. trifoliata* branch was used as the foreground branch, while other branches were used as background branches under the branch‐site model in PAML (Yang, 2007; Jeffares *et al*., 2015). The likelihood ratio test (LRT) was conducted to compare the two models (model A and A1). Genes with an FDR < 0.05 and at least one positively selected site (Bayes probability > 95%) were considered positively selected. To further compare the positively selected genes among cold‐tolerant species, three additional phylogenetic trees were constructed using RAxML and single‐copy genes from three combinations of species, including the three cold‐sensitive species (background branch) and each of the cold‐tolerant species (foreground branch), for positive selection analysis (Table S13; ‘Category 1–3’).

### Re‐annotation of the 10 genomes using the same gene prediction pipeline

To further filter the findings from existing annotations that were produced previously by different annotation pipelines, a homology‐based gene prediction pipeline (Blast/Exonerate) was applied on all the potential genomic regions containing genes of interest for all the genomes, including *P. trifoliata* itself. The protein sequences from *P. trifoliata* were used as the seeding peptides to search for potential genomic regions containing their homologs, including candidate cold signaling‐related genes, significantly expanded gene families, *P. trifoliata*‐specific genes and single‐copy genes used for positive selection analysis. For significantly contracted gene families, the proteins from the species with the largest gene number were used as seeding peptides. The seeding peptides were compared with the 10 genomes using tBlastn/Blastx under E‐value 1e‐05. The hits in each genome were extended for 1 kb on each side (if available), and sequences were extracted for gene prediction using Exonerate (v2.2.0) under the ‘protein2genome’ model. Exonerate was run twice under either a stringent cutoff (‐‐percent 80 ‐n 1) or a less stringent cutoff (‐‐percent 50 ‐n 5). The best scoring predicted proteins were used for further analysis. The exonerate models derived from the less stringent cutoff were used to re‐run gene family assignment and expansion/contraction analysis (mainly involving gene numbers) following the same methods described above. The exonerate models derived from the stringent cutoff were used to re‐run gene family assignment, phylogenetic analysis and positive selection analysis (involving gene CDS). For a few homologs of the key cold signaling pathway genes whose exonerate models were not available for all 10 species, they were manually re‐annotated using AUGUSTUS for all species.

### **Prediction of** ****Poncirus polyandra****  **gene models**

To obtain *P. polyandra* gene models for phylogenetic analysis, a genome assembly of *P. polyandra* short reads was performed using the reference‐based genome assembly and annotation tool (RGAAT; Liu *et al*., 2018b). The reads from whole‐genome sequencing of *P. polyandra* were aligned to the *P. trifoliata* genome using BWA‐MEM. Only uniquely mapped reads were retained for further analysis. Sequence variants were identified using Samtools and Bcftools (Li *et al*., 2009) according to the instructions of RGAAT. RGAAT was run using the sequence variant result with default parameters, except ‘‐m 50’ for comparison with different species. Subsequently, the resulting assembled sequences were used for gene prediction if their matching *P. trifoliata* genomic regions contain a gene with a depth coverage ≥ 3 for all positions. *Poncirus polyandra* genes were predicted by comparing between *P. polyandra* assembled sequences and their corresponding *P. trifoliata* proteins using Genewise in Wise2 (Birney *et al*., 2004). Only predicted genes from Genewise without breaks or frameshift errors were included for downstream analysis.

### **WGD analysis and estimation of divergence within** ****Poncirus**** 

We estimated the WGDs of *P. trifoliata* genome and other *Citrus*‐related genomes based on the *Ks* (number of synonymous substitutions per synonymous site) method using a python pipeline GenoDup (Mao, 2019). For each species, the paralogous group information obtained from results of OrthoMCL, proteins and CDS of genes were used as input with ‘‐n 15’ and other parameters as default. The model plant *A. thaliana* was also included for comparison of results. To estimate the divergence time between *P. trifoliata* and *P. polyandra*, the gene pair information from the previous step, proteins and CDS from the two species were also input into the GenoDup pipeline. The divergence time was estimated using the formula T = *Ks*/2*r*, where the neutral substitution rate *r* was assumed to be 1 ~ 2 × 10^−9^ (Wu *et al*., 2014).

### Re‐sequencing data analysis

Illumina paired‐end reads from five *Poncirus* accessions, six *Poncirus*/*Citrus* hybrids and six citrus cultivars (including sweet orange, two pummelos and three mandarins) were mapped to the haploid *Poncirus* reference sequence V1.3 using BWA‐MEM. Sequences of sweet orange (SWO), Sunki mandarin (SNK) and low acid pummelo (LAP) were from previous publications (Wu *et al*., 2014, 2018). Duplicate reads are removed with sambamba before calling variants with GATK HaplotypeCaller (version 3.7‐0‐gcfedb67; McKenna *et al*., 2010). To reduce variant call errors, the following filters are applied as done previously (Wu *et al*., 2014): variants are bi‐allelic, read mapping quality ≥ 20, base quality ≥ 20, genotype quality ≥ 20, read depth between 1/2 × and 2 × the genome‐wide median, allele balance on heterozygous sites. To determine the chloroplast type of each accession, the Illumina paired‐end reads are mapped to the chloroplast genome sequence of sweet orange (Bausher *et al*., 2006), and variants are called using GATK as above.

For principal component analysis (PCA), Eigensoft (Patterson *et al*., 2006) was used on SNPs in the genic regions with missing rate < 0.05. Admixture analysis was performed with ADMIXTURE (Alexander *et al*., 2009), using the same set of SNPs as in PCA. For relatedness analysis, genome‐wide haplotype sharing between accession pairs were calculated in sliding windows of 100 kb, and the coefficient of relatedness was estimated following Wu *et al*. (2014).

## Author contributions

ZD, FG and DR designed and supervised this project. ZP, JB, GW and SS analyzed the data. JB performed genome assembly. SS and ZP performed genome annotation. ZP performed comparative genomics and evolutionary analyses. GW analyzed re‐sequencing data of *Poncirus* accessions. NR, DD and QY (Qibin Yu) prepared and collected plant materials, isolated DNA and RNA, and/or coordinated sequencing. SP confirmed SNP data of *Poncirus* accessions. QY (Qian You) participated in genome analysis. ZP, GW and JB wrote the original manuscript; ZD, DR, FG, ZP, GW and Qibin Yu critically revised the manuscript. All authors approved the manuscript.

## Conflict of interest

The authors declare no conflict of interest.

## Supporting information


**Figure S1.** Genome‐wide Hi‐C contact map of the *Poncirus trifoliata* V1.3 assembly (X and Y axes) at 250 kb matrix resolution. The nine chromosomes and 143 unplaced scaffolds are numbered and oriented according to Clementine V1.0, and are presented as blue boxes in descending order (from upper left to lower right). Contacts (red pixels) within chromosomes are denser than between chromosomes, with contacts between adjacent genomic loci (pixels nearer to the diagonal) being denser than those at greater inter‐locus distances (pixels farther off of the diagonal). This feature of Hi‐C was exploited to perform the scaffolding shown.

**Figure S2.** Dot‐plot showing segmental correspondence between *P. trifoliata* V1.3 reference sequence (*y*‐axis) and haploid Clementine reference sequence V1.0 (*x*‐axis). Aligned segments between assemblies shown with lines; purple lines indicate alignments between sequences in the same orientation, while light‐blue lines indicate alignments in the reversed orientation.

**Figure S3.** A phylogenetic tree of identified NBS genes in *Poncirus trifoliata*. Three major clusters were identified (blue, orange and purple dashed lines).

**Figure S4.** WGD and divergence between *Poncirus trifoliata* and *Poncirus polyandra*. (a) The *Ks* distribution for paralogous gene groups in *P. trifoliata*, *C. × clementina*, *C. × sinensis* and *Arabidopsis* (as a control). (b) The *Ks* distribution between *P. trifoliata* and *P. polyandra* obtained from the 8326 gene pairs.

**Figure S5.** WGD in other *Citrus*‐related species.

**Figure S6.** Genetic relatedness among four *P. trifoliata* accessions. Each row represents one pair of accessions with codes explained in Figure S5(a). Dark and light green colors denote sharing two and one haplotypes, respectively, whereas gray stands for the absence of haplotype sharing.

**Table S1.** Summary of sequenced *Poncirus* accessions using PacBio and Illumina Hiseq 2500 platforms
**Table S2.** TEs in *P. trifoliata* genome
**Table S3.** Comparison of completeness and statistics of assemblies among all published *Citrus*‐related genomes
**Table S4.** Summary of gene families containing cold signaling‐related genes
**Table S5.** Summary of cold signaling‐related genes recovered by re‐annotation
**Table S6.** Classification of identified NBS genes in *P. trifoliata*
**Table S7.** Summary of identified TF genes in *P. trifoliata* genome
**Table S8.** Gene family assignment of 10 *Citrus*‐related species based on existing annotations
**Table S9.** Genes specific to *P. trifoliata*
**Table 10.** Summary of re‐annotation of single‐copy gene families across 10 genomes
**Table S11.** Summary of rapid evolving gene families in *P. trifoliata* lineage from CAFÉ analysis
**Table S12.** GO enrichment analysis of *P. trifoliata*‐specific genes and fast evolving genes in *P. trifoliata*
**Table S13.** Summary of positively selected genes
**Table S14.** QTLs associated with HLB tolerance mapped to *P. trifoliata* genome and locations of *Ctv* and *Tyr1* regions
**Table S15.** Candidate *P. trifoliata* genes responsive to *C*Las infection and located within QTLs associated with HLB tolerance
**Table S16.** NBS genes located within QTL associated with HLB tolerance and *Ctv* and *Tyr1* regions

## Data Availability

The whole‐genome sequencing and re‐sequencing data from this study have been deposited at GenBank (BioProject: PRJNA648176, SRA accession number: SRR12323696‐SRR12323706). The *Poncirus trifoliata* genome v1.3 has been deposited in *Phytozome* (https://phytozome.jgi.doe.gov/pz/portal.html).

## References

[tpj14993-bib-0001] Abrusán, G. , Grundmann, N. , DeMester, L. and Makalowski, W. (2009) TEclass—a tool for automated classification of unknown eukaryotic transposable elements. Bioinformatics, 25, 1329–1330. 19349283 10.1093/bioinformatics/btp084

[tpj14993-bib-0002] Albiach‐Marti, M.R. , Grosser, J.W. , Gowda, S. , Mawassi, M. , Satyanarayana, T. , Garnsey, S.M. and Dawson, W.O. (2004) *Citrus tristeza virus* replicates and forms infectious virions in protoplasts of resistant citrus relatives. Mol. Breed. 14, 117–128.

[tpj14993-bib-0003] Alexander, D.H. , Novembre, J. and Lange, K. (2009) Fast model‐based estimation of ancestry in unrelated individuals. Genome Res. 19, 1655–1664. 19648217 10.1101/gr.094052.109 PMC2752134

[tpj14993-bib-0004] Bao, W. , Kojima, K.K. and Kohany, O. (2015) Repbase Update, a database of repetitive elements in eukaryotic genomes. Mobile DNA, 6, 11. 26045719 10.1186/s13100-015-0041-9 PMC4455052

[tpj14993-bib-0005] Bausher, M.G. , Singh, N.D. , Lee, S.B. , Jansen, R.K. and Daniell, H. (2006) The complete chloroplast genome sequence of *Citrus sinensis* (L.) Osbeck var ‘Ridge Pineapple’: organization and phylogenetic relationships to other angiosperms. BMC Plant Biol. 6, 21. 17010212 10.1186/1471-2229-6-21 PMC1599732

[tpj14993-bib-0006] Birney, E. , Clamp, M. and Durbin, R. (2004) GeneWise and genomewise. Genome Res. 14, 988–995. 15123596 10.1101/gr.1865504 PMC479130

[tpj14993-bib-0007] Boava, L.P. , Cristofani‐Yaly, M. , Mafra, V.S. , Kubo, K. , Kishi, L.T. , Takita, M.A. , Ribeiro‐Alves, M. and Machado, M.A. (2011) Global gene expression of *Poncirus trifoliata*, *Citrus sunki* and their hybrids under infection of *Phytophthora parasitica* . BMC Genom., 12, 39. 10.1186/1471-2164-12-39 PMC3033816 21241495

[tpj14993-bib-0008] Borredá, C. , Pérez‐Román, E. , Ibanez, V. , Terol, J. and Talon, M. (2019) Reprogramming of retrotransposon activity during speciation of the genus *Citrus* . Genome Biol. Evol. 11, 3478–3495. 31710678 10.1093/gbe/evz246 PMC7145672

[tpj14993-bib-0009] Bowman, K.D. , Faulkner, L. and Kesinger, M. (2016) New citrus rootstocks released by USDA 2001–2010: field performance and nursery characteristics. HortScience, 51, 1208–1214.

[tpj14993-bib-0010] Bové, J.M. (2006) Huanglongbing: a destructive, newly emerging, century‐old disease of citrus. J. Plant Pathol. 88, 7–37.

[tpj14993-bib-0011] Butelli, E. , Licciardello, C. , Zhang, Y. , Liu, J. , Mackay, S. , Bailey, P. , Reforgiato‐Recupero, G. and Martin, C. (2012) Retrotransposons control fruit‐specific, cold‐dependent accumulation of anthocyanins in blood oranges. Plant Cell, 24, 1242–1255. 22427337 10.1105/tpc.111.095232 PMC3336134

[tpj14993-bib-0012] Castle, W.S. , Nunnallee, J. and Manthey, J.A. (2009) Screening citrus rootstocks and related selections in soil and solution culture for tolerance to low‐iron stress. HortScience, 44, 638–645.

[tpj14993-bib-0013] Cameron, J.W. , Baines, R.C. and Oscar, F.C. (1954) Resistance of hybrid seedlings of the trifoliate orange to infestation by the citrus nematode. Phytopathology, 44, 456–458.

[tpj14993-bib-0014] Chaisson, M.J. and Tesler, G. (2012) Mapping single molecule sequencing reads using basic local alignment with successive refinement (BLASR): application and theory. BMC Bioinformatics, 13, 238. 22988817 10.1186/1471-2105-13-238 PMC3572422

[tpj14993-bib-0015] Cheng, F.S. and Roose, M. (1995) Origin and inheritance of dwarfing by the citrus rootstock *Poncirus trifoliata* ‘Flying Dragon’. J. Am. Soc. Hort. Sci. 120, 286–291.

[tpj14993-bib-0016] Cheng, Y. , Guo, W. , Yi, H. , Pang, X. and Deng, X. (2003) An efficient protocol for genomic DNA extraction from *Citrus* species. Plant Mol. Biol. Rep. 21, 177–178.

[tpj14993-bib-0017] Chin, C.S. , Alexander, D.H. , Marks, P. ***et al*** (2013) Nonhybrid, finished microbial genome assemblies from long‐read SMRT sequencing data. Nat. Methods, 10, 563. 23644548 10.1038/nmeth.2474

[tpj14993-bib-0118] Chin, C.S. , Peluso, P. , Sedlazeck, F.J. ***et al*** (2016) Phased diploid genome assembly with single‐molecule real‐time sequencing. Nat. Meth. 13, 1050–1054. 10.1038/nmeth.4035 PMC5503144 27749838

[tpj14993-bib-0018] Crifò, T. , Puglisi, I. , Petrone, G. , Recupero, G.R. and Lo Piero, A.R. (2011) Expression analysis in response to low temperature stress in blood oranges: implication of the flavonoid biosynthetic pathway. Gene, 476, 1–9. 21349317 10.1016/j.gene.2011.02.005

[tpj14993-bib-0019] De Bie, T. , Cristianini, N. , Demuth, J.P. and Hahn, M.W. (2006) CAFE: a computational tool for the study of gene family evolution. Bioinformatics, 22, 1269–1271. 16543274 10.1093/bioinformatics/btl097

[tpj14993-bib-0020] Delorenzi, M. and Speed, T. (2002) An HMM model for coiled‐coil domains and a comparison with PSSM‐based predictions. Bioinformatics, 18, 617–625. 12016059 10.1093/bioinformatics/18.4.617

[tpj14993-bib-0021] Deng, Z. , Huang, S. , Ling, P. , Yu, C. , Tao, Q. , Chen, C. , Wendell, M.K. , Zhang, H.B. and Gmitter, F.G. Jr (2001) Fine genetic mapping and BAC contig development for the citrus tristeza virus resistance gene locus in *Poncirus trifoliata* (Raf.). Mol. Genet. Genomics, 265, 739–747. 11459195 10.1007/s004380100471

[tpj14993-bib-0022] Ding, S. , Zhang, X. , Bao, Z. and Ling, M. (1984) A new species of *Poncirus* from China. Acta Bot. Yunnan. 6, 292–293.

[tpj14993-bib-0023] Duarte, J.M. , Wall, P.K. , Landherr, L.L. , Ma, H. , Depamphilis, C.W. , Edger, P.P. , Pires, J.C. and Leebens‐Mack, J. (2010) Identification of shared single copy nuclear genes in *Arabidopsis*, *Populus*, *Vitis* and *Oryza* and their phylogenetic utility across various taxonomic levels. BMC Evol. Biol. 10, 61. 20181251 10.1186/1471-2148-10-61 PMC2848037

[tpj14993-bib-0024] Ducharme, E.P. (1948) Resistance of *Poncirus trifoliata* rootstock to nematode infestation in Argentina. Citrus Ind. 29, 9–15.

[tpj14993-bib-0025] Dudchenko, O. , Shamim, M.S. , Batra, S. ***et al*** (2018) The Juicebox Assembly Tools module facilitates de novo assembly of mammalian genomes with chromosome‐length scaffolds for under $1000. BioRxiv, 254797 10.1101/254797

[tpj14993-bib-0026] Durand, N.C. , Robinson, J.T. , Shamim, M.S. , Machol, I. , Mesirov, J.P. , Lander, E.S. and Aiden, E.L. (2016a) Juicebox provides a visualization system for Hi‐C contact maps with unlimited zoom. Cell Syst. 3, 99–101. 27467250 10.1016/j.cels.2015.07.012 PMC5596920

[tpj14993-bib-0027] Durand, N.C. , Shamim, M.S. , Machol, I. , Rao, S.S.P. , Huntley, M.H. , Lander, E.S. and Aiden, E.L. (2016b) Juicer provides a one‐click system for analyzing loop‐resolution Hi‐C experiments. Cell Syst. 3, 95–98. 27467249 10.1016/j.cels.2016.07.002 PMC5846465

[tpj14993-bib-0028] Ellinghaus, D. , Kurtz, S. and Willhoeft, U. (2008) LTRharvest, an efficient and flexible software for de novo detection of LTR retrotransposons. BMC Bioinformatics, 9, 18. 18194517 10.1186/1471-2105-9-18 PMC2253517

[tpj14993-bib-0029] Finn, R.D. , Clements, J. and Eddy, S.R. (2011) HMMER web server: interactive sequence similarity searching. Nucleic Acids Res. 39, W29–W37. 21593126 10.1093/nar/gkr367 PMC3125773

[tpj14993-bib-0030] Finn, R.D. , Coggill, P. , Eberhardt, R.Y. , Eddy, S.R. , Mistry, J. , Mitchell, A.L. , Potter, S.C. , Punta, M. , Qureshi, M. and Sangrador‐Vegas, A. (2016) The Pfam protein families database: towards a more sustainable future. Nucleic Acids Res. 44, D279–D285. 26673716 10.1093/nar/gkv1344 PMC4702930

[tpj14993-bib-0031] Fischer, S. , Brunk, B.P. , Chen, F. , Gao, X. , Harb, O.S. , Iodice, J.B. , Shanmugam, D. , Roos, D.S. and Stoeckert, C.J. Jr (2011) Using OrthoMCL to assign proteins to OrthoMCL‐DB groups or to cluster proteomes into new ortholog groups. Curr. Protoc. Bioinformatics, 35, 1–19. 10.1002/0471250953.bi0612s35 PMC3196566 21901743

[tpj14993-bib-0032] Florida Department of Agriculture and Consumer Services Bureau of Citrus Budwood Registration (2019) Citrus budwood registration annual report 2018–2019. Chiefland, FL Available at: https://www.fdacs.gov/Divisions‐Offices/Plant‐Industry/Agriculture‐Industry/Citrus‐Health‐Response‐Program/Citrus‐Budwood‐Program/Annual‐Reports.

[tpj14993-bib-0033] Fu, S. , Shao, J. , Zhou, C. and Hartung, J.S. (2016) Transcriptome analysis of sweet orange trees infected with ‘*Candidatus* Liberibacter asiaticus’ and two strains of *Citrus Tristeza Virus* . BMC Genom., 17, 349. 10.1186/s12864-016-2663-9 PMC4865098 27169471

[tpj14993-bib-0034] Garnsey, D.J. and Barrett, S.M. (1987) Identification of citrus tristeza virus resistance in citrus relatives and its potential applications. Phytophylactica, 19, 187–192.

[tpj14993-bib-0035] Garrison, E. and Marth, G. (2012) Haplotype‐based variant detection from short‐read sequencing. arXiv preprint, arXiv, 1207–3907.

[tpj14993-bib-0036] George, J. and Lapointe, S.L. (2019) Host‐plant resistance associated with *Poncirus trifoliata* influence oviposition, development and adult emergence of *Diaphorina citri* (Hemiptera: Liviidae). Pest Manag. Sci. 75, 279–285. 29885090 10.1002/ps.5113

[tpj14993-bib-0037] Gilmour, S.J. , Zarka, D.G. , Stockinger, E.J. , Salazar, M.P. , Houghton, J.M. and Thomashow, M.F. (1998) Low temperature regulation of the *Arabidopsis* CBF family of AP2 transcriptional activators as an early step in cold‐induced COR gene expression. Plant J. 16, 433–442. 9881163 10.1046/j.1365-313x.1998.00310.x

[tpj14993-bib-0038] Gmitter, F.G. Jr and Hu, X. (1990) The possible role of Yunnan, China, in the origin of contemporary *Citrus* species (Rutaceae). Econ. Bot. 44, 267–277.

[tpj14993-bib-0039] Gmitter, F.G. Jr , Xiao, S. , Huang, S. , Hu, X.L. , Garnsey, S.M. and Deng, Z. (1996) A localized linkage map of the citrus tristeza virus resistance gene region. Theor. Appl. Genet. 92, 688–695. 24166392 10.1007/BF00226090

[tpj14993-bib-0040] Gong, X.Q. and Liu, J. (2013) Genetic transformation and genes for resistance to abiotic and biotic stresses in *Citrus* and its related genera. Plant Cell Tissue Organ Cult. 113, 137–147.

[tpj14993-bib-0041] Graham, J.H. (1990) Evaluation of tolerance of citrus rootstocks to Phytophthora root rot in chlamydospore‐infested soil. Plant Dis. 74, 743–746.

[tpj14993-bib-0042] Graham, J.H. , Bright, D.B. and McCoy, C.W. (2003) *Phytophthora*‐*Diaprepes* weevil complex: *Phytophthora* spp. relationship with citrus rootstocks. Plant Dis. 87, 85–90. 30812706 10.1094/PDIS.2003.87.1.85

[tpj14993-bib-0043] Guo, X. , Liu, D. and Chong, K. (2018) Cold signaling in plants: insights into mechanisms and regulation. J. Integr. Plant Biol. 60, 745–756. 30094919 10.1111/jipb.12706

[tpj14993-bib-0044] Haas, B.J. , Delcher, A.L. , Mount, S.M. , Wortman, J.R. , Smith, R.K. Jr , Hannick, L.I. , Maiti, R. , Ronning, C.M. , Rusch, D.B. and Town, C.D. (2003) Improving the *Arabidopsis* genome annotation using maximal transcript alignment assemblies. Nucleic Acids Res. 31, 5654–5666. 14500829 10.1093/nar/gkg770 PMC206470

[tpj14993-bib-0045] Hashida, S.N. , Uchiyama, T. , Martin, C. , Kishima, Y. , Sano, Y. and Mikami, T. (2006) The temperature‐dependent change in methylation of the Antirrhinum transposon Tam3 is controlled by the activity of its transposase. Plant Cell, 18, 104–118. 16326924 10.1105/tpc.105.037655 PMC1323487

[tpj14993-bib-0046] Hashida, S.N. , Kitamura, K. , Mikami, T. and Kishima, Y. (2003) Temperature shift coordinately changes the activity and the methylation state of transposon Tam3 in Antirrhinum majus. Plant Physiol. 132, 1207–1216. 12857803 10.1104/pp.102.017533 PMC167061

[tpj14993-bib-0047] Hoff, K.J. , Lange, S. , Lomsadze, A. , Borodovsky, M. and Stanke, M. (2015) BRAKER1: unsupervised RNA‐Seq‐based genome annotation with GeneMark‐ET and AUGUSTUS. Bioinformatics, 32, 767–769. 26559507 10.1093/bioinformatics/btv661 PMC6078167

[tpj14993-bib-0048] Hu, Y. , Zhong, X. , Liu, X. , Lou, B. , Zhou, C. and Wang, X. (2017) Comparative transcriptome analysis unveils the tolerance mechanisms of *Citrus hystrix* in response to *'Candidatus* Liberibacter asiaticus' infection. PLoS One, 12, e0189229. 29232716 10.1371/journal.pone.0189229 PMC5726760

[tpj14993-bib-0049] Huang, M. , Roose, M.L. , Yu, Q. , Du, D. , Yu, Y. , Zhang, Y. , Deng, Z. , Stover, E. and Gmitter, F.G. Jr (2018) Construction of high‐density genetic maps and detection of QTLs associated with Huanglongbing tolerance in citrus. Front. Plant Sci. 9, 1694. 30542355 10.3389/fpls.2018.01694 PMC6278636

[tpj14993-bib-0050] Huang, X. , Li, K. , Jin, C. and Zhang, S. (2015) ICE1 of *Pyrus ussuriensis* functions in cold tolerance by enhancing PuDREBa transcriptional levels through interacting with PuHHP1. Sci. Rep. 5, 17620. 26626798 10.1038/srep17620 PMC4667267

[tpj14993-bib-0051] Huang, X. , Wang, W. , Zhang, Q. and Liu, J. (2013) A basic helix‐loop‐helix transcription factor, PtrbHLH, of *Poncirus trifoliata* confers cold tolerance and modulates peroxidase‐mediated scavenging of hydrogen peroxide. Plant Physiol. 162, 1178–1194. 23624854 10.1104/pp.112.210740 PMC3668048

[tpj14993-bib-0052] Jeffares, D.C. , Tomiczek, B. , Sojo, V. and dos Reis, M. (2015) A beginners guide to estimating the non‐synonymous to synonymous rate ratio of all protein‐coding genes in a genome. Methods Mol. Biol. 1201, 65–90. 25388108 10.1007/978-1-4939-1438-8_4

[tpj14993-bib-0053] Kajitani, R. , Toshimoto, K. , Noguchi, H. ***et al*** (2014) Efficient *de novo* assembly of highly heterozygous genomes from whole‐genome shotgun short reads. Genome Res. 24, 1384–1395. 24755901 10.1101/gr.170720.113 PMC4120091

[tpj14993-bib-0054] Kamiri, M. , Stift, M. , Costantino, G. , Dambier, D. , Kabbage, T. , Ollitrault, P. and Froelicher, Y. (2018) Preferential homologous chromosome pairing in a tetraploid intergeneric somatic hybrid (*Citrus reticulata* + *Poncirus trifoliata*) revealed by molecular marker inheritance. Front. Plant Sci. 9, 1557. 30450106 10.3389/fpls.2018.01557 PMC6224360

[tpj14993-bib-0055] Kidwell, M.G. and Lisch, D. (1997) Transposable elements as sources of variation in animals and plants. Proc. Natl Acad. Sci. USA, 94, 7704–7711. 9223252 10.1073/pnas.94.15.7704 PMC33680

[tpj14993-bib-0056] Kim, K.E. , Peluso, P. , Babayan, P. ***et al*** (2014) Long‐read, whole‐genome shotgun sequence data for five model organisms. Sci. Data, 1, 14005. 10.1038/sdata.2014.45 PMC4365909 25977796

[tpj14993-bib-0057] Kitajima, E.W. , Silva, D.M. , Oliveira, A.R. , Müller, G.W. and Costa, A.S. (1964) Thread‐like particles associated with tristeza disease of citrus. Nature, 201, 1011–1012. 14191569 10.1038/2011011a0

[tpj14993-bib-0058] Katoh, K. , Kuma, K. , Toh, H. and Miyata, T. (2005) MAFFT version 5: improvement in accuracy of multiple sequence alignment. Nucleic Acids Res. 33, 511–518. 15661851 10.1093/nar/gki198 PMC548345

[tpj14993-bib-0059] Krueger, R.R. and Navarro, L. (2007) Citrus germplasm resources In Citrus Genetics, Breeding and Biotechnology (Khan I.A., ed). Wallingford, UK: CAB International, pp. 45–140.

[tpj14993-bib-0060] Kumar, S. , Stecher, G. and Tamura, K. (2016) MEGA7: molecular evolutionary genetics analysis version 7.0 for bigger datasets. Mol. Biol. Evol. 33, 1870–1874. 27004904 10.1093/molbev/msw054 PMC8210823

[tpj14993-bib-0061] Li, S. , Zong, D. , Zhou, A. and He, C. (2019) The complete chloroplast genome sequence of *Poncirus polyandra* (Rutaceae), an endangered species endemic to Yunnan Province, China. Mitochondrial DNA B, 4, 766–768.

[tpj14993-bib-0062] Li, H. (2013) Aligning sequence reads, clone sequences and assembly contigs with BWA‐MEM. arXiv preprint, arXiv, 1303–3997.

[tpj14993-bib-0063] Li, H. , Handsaker, B. , Wysoker, A. , Fennell, T. , Ruan, J. , Homer, N. , Marth, G. , Abecasis, G. and Durbin, R. (2009) The Sequence Alignment/Map format and SAMtools. Bioinformatics, 25, 2078–2079. 19505943 10.1093/bioinformatics/btp352 PMC2723002

[tpj14993-bib-0064] Li, H. (2018) Minimap2: pairwise alignment for nucleotide sequences. Bioinformatics, 34, 3094–3100. 29750242 10.1093/bioinformatics/bty191 PMC6137996

[tpj14993-bib-0065] Ling, P. , Duncan, L.W. , Deng, Z. , Dunn, D. , Hu, X. , Huang, S. and Gmitter, F.G. Jr (2000) Inheritance of citrus nematode resistance and its linkage with molecular markers. Theor. Appl. Genet. 100, 1010–1017.

[tpj14993-bib-0066] Liu, J. , Shi, Y. and Yang, S. (2018a) Insights into the regulation of C‐repeat binding factors in plant cold signaling. J. Integr. Plant Biol. 60, 780–795. 29667328 10.1111/jipb.12657

[tpj14993-bib-0067] Liu, W. , Wu, S. , Lin, Q. , Gao, S. , Ding, F. , Zhang, X. , Aljohi, H.A. , Yu, J. and Hu, S. (2018b) RGAAT: a reference‐based genome assembly and annotation tool for new genomes and upgrade of known genomes. Genomics Proteomics Bioinformatics, 16, 373–381. 30583062 10.1016/j.gpb.2018.03.006 PMC6364042

[tpj14993-bib-0068] Liu, Q. , Kasuga, M. , Sakuma, Y. , Abe, H. , Miura, S. , Yamaguchi‐Shinozaki, K. and Shinozaki, K. (1998) Two transcription factors, *DREB1* and *DREB2*, with an EREBP/AP2 DNA binding domain separate two cellular signal transduction pathways in drought‐ and low‐ temperature‐responsive gene expression, respectively, in *Arabidopsis* . Plant Cell, 10, 1391–1406. 9707537 10.1105/tpc.10.8.1391 PMC144379

[tpj14993-bib-0069] Mao, Y. (2019) GenoDup Pipeline: a tool to detect genome duplication using the dS‐based method. PeerJ, 7, e6303. 30697488 10.7717/peerj.6303 PMC6347962

[tpj14993-bib-0070] McHale, L. , Tan, X. , Koehl, P. and Michelmore, R.W. (2006) Plant NBS‐LRR proteins: adaptable guards. Genome Biol. 7, 1–11. 10.1186/gb-2006-7-4-212 PMC1557992 16677430

[tpj14993-bib-0071] McKenna, A. , Hanna, M. , Banks, E. ***et al*** (2010) The Genome Analysis Toolkit: a MapReduce framework for analyzing next‐generation DNA sequencing data. Genome Res. 20, 1297–1303. 20644199 10.1101/gr.107524.110 PMC2928508

[tpj14993-bib-0072] Nesom, G. (2014) *Citrus trifoliata* (Rutaceae): Review of biology and distribution in the USA. Phytoneuron, 46, 1–14.

[tpj14993-bib-0073] Nordby, H.E. and Yelenosky, G. (1982) Relationships of leaf fatty acids to cold hardening of citrus seedlings. Plant Physiol. 70, 132–135. 16662433 10.1104/pp.70.1.132 PMC1067100

[tpj14993-bib-0120] Ou, S. and Jiang, N. (2018) LTR_retriever: a highly accurate and sensitive program for identification of long terminal repeat retrotransposons. Plant Physiol. 176, 1410–1422. 29233850 10.1104/pp.17.01310 PMC5813529

[tpj14993-bib-0074] Oustric, J. , Morillon, R. , Luro, F. , Herbette, S. , Lourkisti, R. , Giannettini, J. , Berti, L. and Santini, J. (2017) Tetraploid Carrizo citrange rootstock (*Citrus sinensis* Osb. × *Poncirus trifoliata* L. Raf.) enhances natural chilling stress tolerance of common clementine (*Citrus clementina* Hort. ex Tan). J. Plant Physiol. 214, 108–115. 28478318 10.1016/j.jplph.2017.04.014

[tpj14993-bib-0075] Padmanabhan, M. , Cournoyer, P. and Dinesh‐Kumar, S.P. (2009) The leucine‐rich repeat domain in plant innate immunity: a wealth of possibilities. Cell. Microbiol. 11, 191–198. 19016785 10.1111/j.1462-5822.2008.01260.x PMC2762402

[tpj14993-bib-0076] Pang, X. , Hu, C. and Deng, X. (2003) Phylogenetic relationships among citrus and its relatives as revealed by SSR markers. Acta Genet. Sin. 30, 81–87. 12812081

[tpj14993-bib-0077] Park, S. , Gilmour, S.J. , Grumet, R. and Thomashow, M.F. (2018) CBF‐dependent and CBF‐independent regulatory pathways contribute to the differences in freezing tolerance and cold‐regulated gene expression of two Arabidopsis ecotypes locally adapted to sites in Sweden and Italy. PLoS One, 13, e0207723. 30517145 10.1371/journal.pone.0207723 PMC6281195

[tpj14993-bib-0078] Park, S. , Lee, C. , Doherty, C.J. , Gilmour, S.J. , Kim, Y. and Thomashow, M.F. (2015) Regulation of the *Arabidopsis* CBF regulon by a complex low‐temperature regulatory network. Plant J. 82, 193–207. 25736223 10.1111/tpj.12796

[tpj14993-bib-0079] Patterson, N. , Price, A.L. and Reich, D. (2006) Population structure and eigenanalysis. PLOS Genet. 2, 2074–2093. 10.1371/journal.pgen.0020190 PMC1713260 17194218

[tpj14993-bib-0080] Peng, T. , Jia, M. and Liu, J. (2015) RNAi‐based functional elucidation of *PtrPRP*, a gene encoding a hybrid proline rich protein, in cold tolerance of *Poncirus trifoliata* . Front. Plant Sci. 6, 808. 26483822 10.3389/fpls.2015.00808 PMC4587090

[tpj14993-bib-0081] Pfeil, B.E. and Crisp, M.D. (2008) The age and biogeography of *Citrus* and the orange subfamily (Rutaceae: Aurantioideae) in Australasia and New Caledonia. Am. J. Bot. 95, 1621–1631. 21628168 10.3732/ajb.0800214

[tpj14993-bib-0082] Quinlan, A.R. and Hall, I.M. (2010) BEDTools: a flexible suite of utilities for comparing genomic features. Bioinformatics, 26, 841–842. 20110278 10.1093/bioinformatics/btq033 PMC2832824

[tpj14993-bib-0083] Radhika, G. , Sudha, V. , Mohan Sathya, R. , Ganesan, A. and Mohan, V. (2008) Association of fruit and vegetable intake with cardiovascular risk factors in urban south Indians. Br. J. Nutr. 99, 398–405. 17678569 10.1017/S0007114507803965

[tpj14993-bib-0084] Rahman, A. , Siddiqui, S.A. , Jakhar, R. and Kang, S.C. (2015) Growth inhibition of various human cancer cell lines by imperatorin and limonin from *Poncirus trifoliata* Raf. Seeds. Anticancer Agents Med. Chem. 15, 236–241. 25244913 10.2174/1871520614666140922122358

[tpj14993-bib-0085] Sahin‐Cevik, M. and Moore, G.A. (2006) Identification and expression analysis of cold‐regulated genes from the cold‐hardy *Citrus* relative *Poncirus trifoliata* (L.) Raf. Plant Mol. Biol. 62, 83–97. 16900323 10.1007/s11103-006-9005-2

[tpj14993-bib-0086] Salamov, A.A. and Solovyev, V.V. (2000) *Ab initio* gene finding in *Drosophila* genomic DNA. Genome Res. 10, 516–522. 10779491 10.1101/gr.10.4.516 PMC310882

[tpj14993-bib-0087] Shi, Y. , Ding, Y. and Yang, S. (2018) Molecular regulation of CBF signaling in cold acclimation. Trends Plant Sci. 23, 623–637. 29735429 10.1016/j.tplants.2018.04.002

[tpj14993-bib-0088] Shi, Y. , Ding, Y. and Yang, S. (2015) Cold signal transduction and its interplay with phytohormones during cold acclimation. Plant Cell Physiol. 56, 7–15. 25189343 10.1093/pcp/pcu115

[tpj14993-bib-0089] Shu, S. , Goodstein, D.M. and Rokhsar, D. (2013) PERTRAN: genome‐guided RNA‐seq Read Assembler. Methods, 40, 50.

[tpj14993-bib-0090] Smit, A.F. and Hubley, R. (2010) RepeatModeler Open‐1.0. Available at: www.repeatmasker.org.

[tpj14993-bib-0091] Stamatakis, A. (2014) RAxML version 8: a tool for phylogenetic analysis and post‐analysis of large phylogenies. Bioinformatics, 30, 1312–1313. 24451623 10.1093/bioinformatics/btu033 PMC3998144

[tpj14993-bib-0092] Spiegel‐Roy, P. and Goldschmidt, E.E. (1996) The Biology of Citrus. Cambridge, UK: Cambridge University Press.

[tpj14993-bib-0093] Talavera, G. and Castresana, J. (2007) Improvement of phylogenies after removing divergent and ambiguously aligned blocks from protein sequence alignments. Syst. Biol. 56, 564–577. 17654362 10.1080/10635150701472164

[tpj14993-bib-0094] Talon, M. and Gmitter, F.G. (2008) Citrus genomics. Int. J. Plant Genomics, 2008, 528361. 18509486 10.1155/2008/528361 PMC2396216

[tpj14993-bib-0095] Tanaka, H. , Yamada, S. and Nakanishi, J. (1971) Approach to eliminating tristeza virus from citrus trees by using trifoliate orange seedlings. Bull. Hort. Res. Sta. Jpn. 11, 157–165.

[tpj14993-bib-0096] Tarailo‐Graovac, M. and Chen, N. (2009) Using RepeatMasker to identify repetitive elements in genomic sequences. Curr. Protoc. Bioinformatics, 25, 1–14. 10.1002/0471250953.bi0410s25 19274634

[tpj14993-bib-0097] Tian, L. , Wu, Q.S. , Kuča, K. and Rahman, M.M. (2018) Responses of four citrus plants to phytophthora‐induced root rot. Sains. Malays. 47, 1693–1700.

[tpj14993-bib-0098] Trejo‐Pech, C.J.O. , Spreen, T.H. and Zansler, M.L. (2018) Is growing oranges in Florida a good investment? Am. J. Agric. Econ. 100, 625–639.

[tpj14993-bib-0099] Wang, L. , He, F. , Huang, Y. ***et al*** (2018a) Genome of wild mandarin and domestication history of mandarin. Mol. Plant, 11, 1024–1037. 29885473 10.1016/j.molp.2018.06.001

[tpj14993-bib-0100] Wang, M. , Dai, W. , Du, J. , Ming, R. , Dahro, B. and Liu, J. (2018b) ERF 109 of trifoliate orange (*Poncirus trifoliata* (L.) Raf.) contributes to cold tolerance by directly regulating expression of *Prx1* involved in antioxidative process. Plant Biotechnol. J. 17, 1316–1332. 10.1111/pbi.13056 PMC6576027 30575255

[tpj14993-bib-0101] Wang, M. , Zhang, X. and Liu, J. (2015) Deep sequencing‐based characterization of transcriptome of trifoliate orange (*Poncirus trifoliata* (L.) Raf.) in response to cold stress. BMC Genom., 16, 555. 10.1186/s12864-015-1629-7 PMC4518522 26219960

[tpj14993-bib-0102] Wang, X. , Xu, Y. , Zhang, S. ***et al*** (2017) Genomic analyses of primitive, wild and cultivated citrus provide insights into asexual reproduction. Nat. Genet. 49, 765–772. 28394353 10.1038/ng.3839

[tpj14993-bib-0103] Wu, G.A. , Prochnik, S. , Jenkins, J. ***et al*** (2014) Sequencing of diverse mandarin, pummelo and orange genomes reveals complex history of admixture during citrus domestication. Nat. Biotechnol. 32, 656. 24908277 10.1038/nbt.2906 PMC4113729

[tpj14993-bib-0104] Wu, G.A. , Terol, J. , Ibanez, V. ***et al*** (2018) Genomics of the origin and evolution of *Citrus* . Nature, 554, 311–316. 29414943 10.1038/nature25447

[tpj14993-bib-0105] Xu, Q. , Chen, L. , Ruan, X. ***et al*** (2013) The draft genome of sweet orange (*Citrus sinensis*). Nat. Genet. 45, 59–66. 23179022 10.1038/ng.2472

[tpj14993-bib-0106] Xu, Z. and Wang, H. (2007) LTR_FINDER: an efficient tool for the prediction of full‐length LTR retrotransposons. Nucleic Acids Res. 35, W265–W268. 17485477 10.1093/nar/gkm286 PMC1933203

[tpj14993-bib-0107] Yang, Y. , Wu, H. , Zhao, L. and He, S. (2019) Analysis of *Poncirus polyandra* (Rutaceae) chloroplast genome and its phylogenetic implications. Mitochondrial DNA B, 4, 2287–2288. 10.1080/23802359.2019.1627928 PMC7687512 33365508

[tpj14993-bib-0108] Yang, Z. (2007) PAML 4: phylogenetic analysis by maximum likelihood. Mol. Biol. Evol. 24, 1586–1591. 17483113 10.1093/molbev/msm088

[tpj14993-bib-0109] Yang, Z. , Ye, X. , Choi, S. , Molina, J. , Moonan, F. , Wing, R.A. , Roose, M.L. and Mirkov, T. (2001) Construction of a 1.2‐Mb contig including the citrus tristeza virus resistance gene locus using a bacterial artificial chromosome library of *Poncirus trifoliata* (L.) Raf. Genome, 44, 382–393. 11444697

[tpj14993-bib-0110] Yang, Z. , Ye, X. , Molina, J. , Roose, M.L. and Mirkov, T.E. (2003) Sequence analysis of a 282‐kilobase region surrounding the citrus tristeza virus resistance gene (*Ctv*) locus in *Poncirus trifoliata* L. Raf. Plant Physiol. 131, 482–492. 12586873 10.1104/pp.011262 PMC166825

[tpj14993-bib-0111] Yeh, R.F. , Lim, L.P. and Burge, C.B. (2001) Computational inference of homologous gene structures in the human genome. Genome Res. 11, 803–816. 11337476 10.1101/gr.175701 PMC311055

[tpj14993-bib-0119] Yi, J.M. , Kim, M. , Koo, H.N. , Song, B.K. , Yoo, Y.H. and Kim, H.M. (2004) Poncirus trifoliata fruit induces apoptosis in human promyelocytic leukemia cells. Clin. Chim. Acta. 340, 179–185. 14734210 10.1016/j.cccn.2003.10.017

[tpj14993-bib-0112] Yu, Q. , Chen, C. , Du, D. , Huang, M. , Yao, J. , Yu, F. , Brlansky, R.H. and Gmitter, F.G. (2017) Reprogramming of a defense signaling pathway in rough lemon and sweet orange is a critical element of the early response to ‘*Candidatus* Liberibacter asiaticus’. Hortic. Res. 4, 1–15. 10.1038/hortres.2017.63 PMC5705785 29214028

[tpj14993-bib-0113] Zhang, C. , Rabiee, M. , Sayyari, E. and Mirarab, S. (2018) ASTRAL‐III: polynomial time species tree reconstruction from partially resolved gene trees. BMC Bioinformatics, 19, 153. 29745866 10.1186/s12859-018-2129-y PMC5998893

[tpj14993-bib-0114] Zhang, N. , Zeng, L. , Shan, H. and Ma, H. (2012) Highly conserved low‐copy nuclear genes as effective markers for phylogenetic analyses in angiosperms. New Phytol. 195, 923–937. 22783877 10.1111/j.1469-8137.2012.04212.x

[tpj14993-bib-0115] Zhou, H. , Hirata, M. , Osawa, R. , Fujino, K. and Kishima, Y. (2017) Detainment of Tam3 transposase at plasma membrane by its BED‐zinc finger domain. Plant Physiol. 173, 1492–1501. 28008001 10.1104/pp.16.00996 PMC5291012

[tpj14993-bib-0116] Zhu, C. , Zheng, X. , Huang, Y. ***et al*** (2019) Genome sequencing and CRISPR /Cas9 gene editing of an early flowering mini‐*Citrus* (*Fortunella hindsii*). Plant Biotechnol. J. 17, 2199–2210. 31004551 10.1111/pbi.13132 PMC6790359

[tpj14993-bib-0117] Zhu, S. , Wang, F. , Shen, W. , Jiang, D. , Hong, Q. and Zhao, X. (2015) Genetic diversity of *Poncirus* and phylogenetic relationships with its relatives revealed by SSR and SNP/InDel markers. Acta Physiol. Plant. 37, 141.

