# Supplementary figures and images for: A chromosome‐scale reference genome of trifoliate orange (Poncirus trifoliata) provides insights into disease resistance, cold tolerance and genome evolution in Citrus
Source: Plant J. 2020 Oct 18;104(5):1215–32. doi: 10.1111/tpj.14993 (PMC7756384; doi:10.1111/tpj.14993)

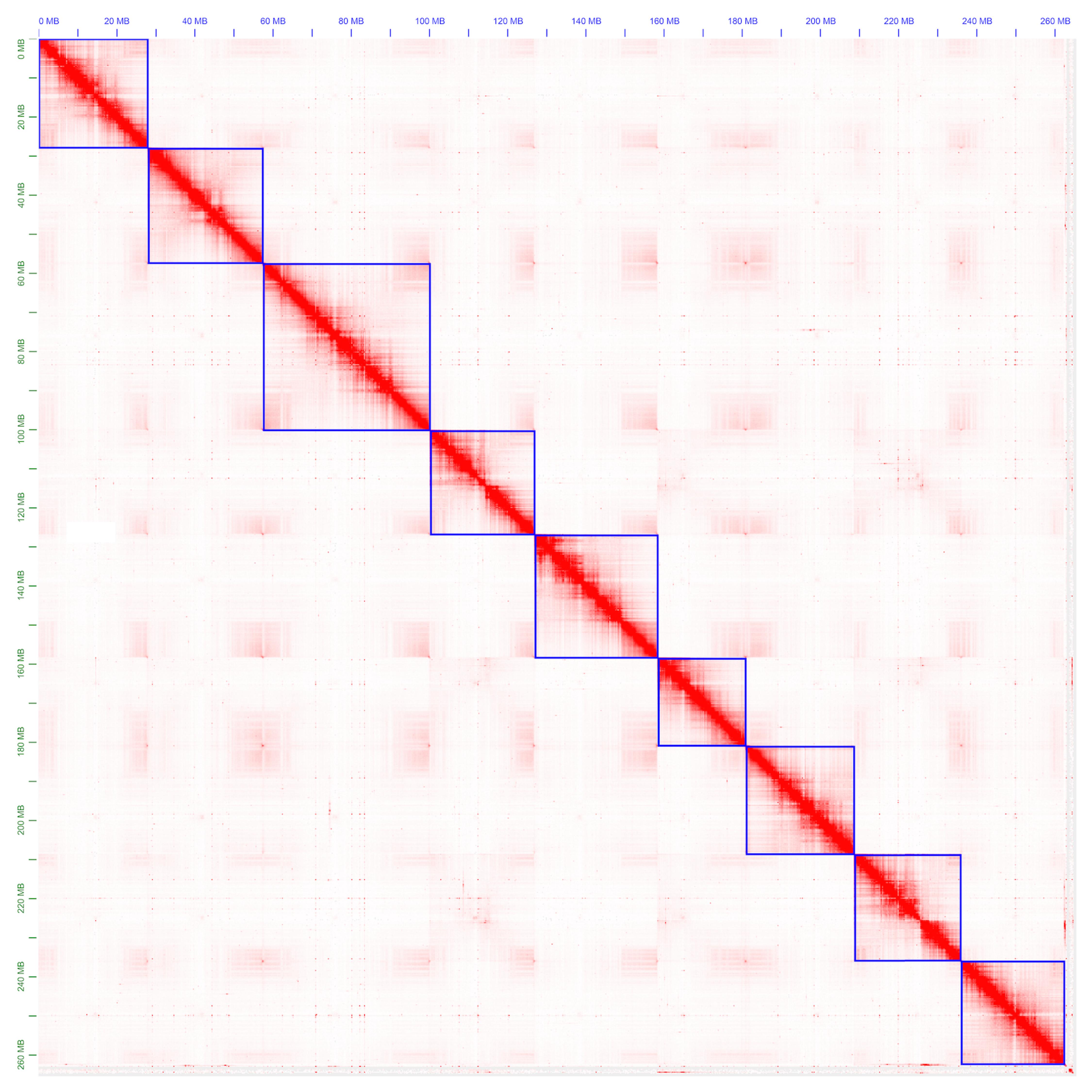

Supplement: Supplementary file 1 — Figure S1. Genome‐wide Hi‐C contact map of the Poncirus trifoliata V1.3 assembly (X and Y axes) at 250 kb matrix resolution. The nine chromosomes and 143 unplaced scaffolds are numbered and oriented according to Clementine V1.0, and are presented as blue boxes in descending order (from upper left to lower right). Contacts (red pixels) within chromosomes are denser than between chromosomes, with contacts between adjacent genomic loci (pixels nearer to the diagonal) being denser than those at greater inter‐locus distances (pixels farther off of the diagonal). This feature of Hi‐C was exploited to perform the scaffolding shown. [file TPJ-104-1215-s001.jpg]

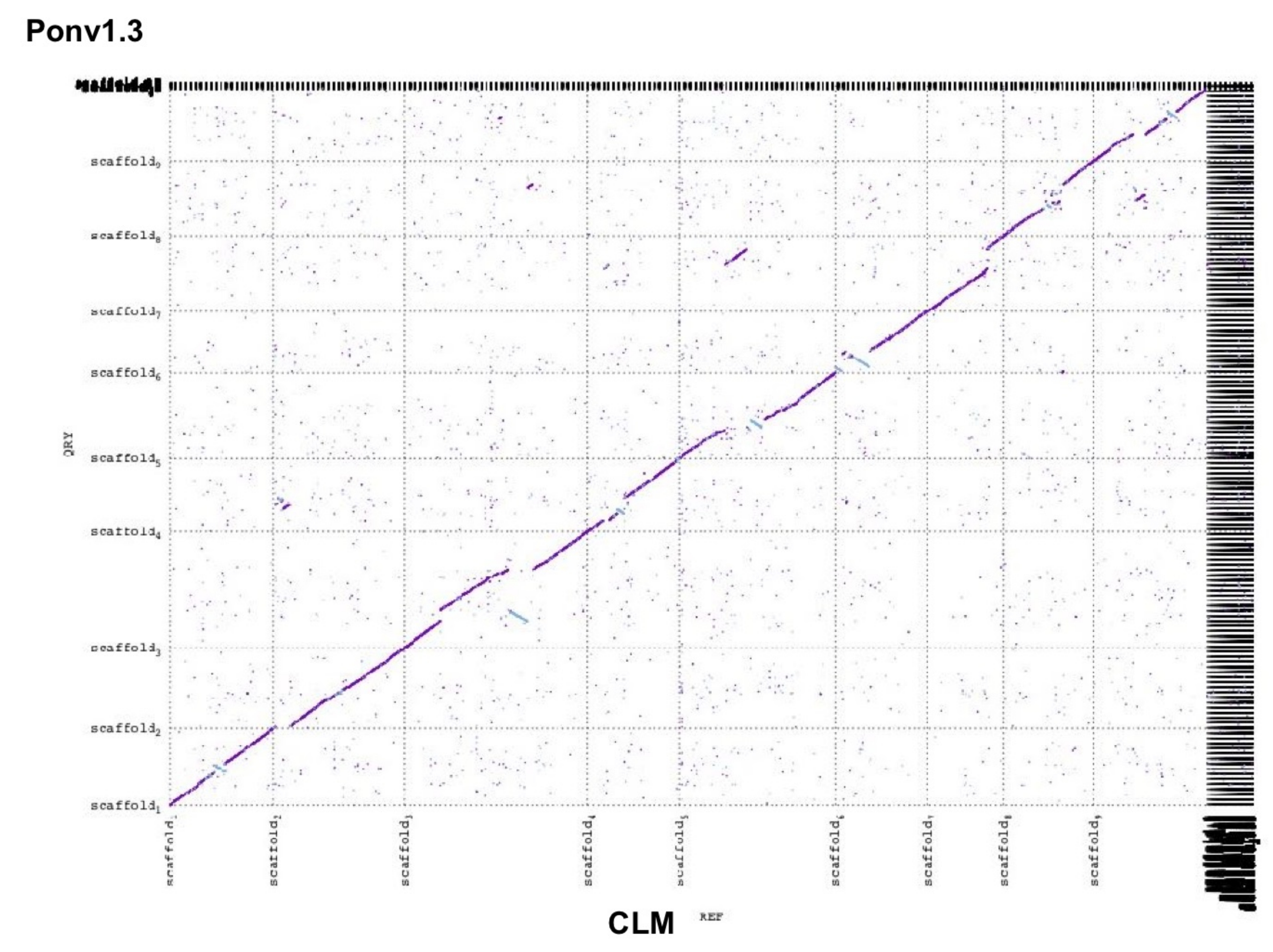

Supplement: Supplementary file 2 — Figure S2. Dot‐plot showing segmental correspondence between P. trifoliata V1.3 reference sequence (y‐axis) and haploid Clementine reference sequence V1.0 (x‐axis). Aligned segments between assemblies shown with lines; purple lines indicate alignments between sequences in the same orientation, while light‐blue lines indicate alignments in the reversed orientation. [file TPJ-104-1215-s002.jpg]

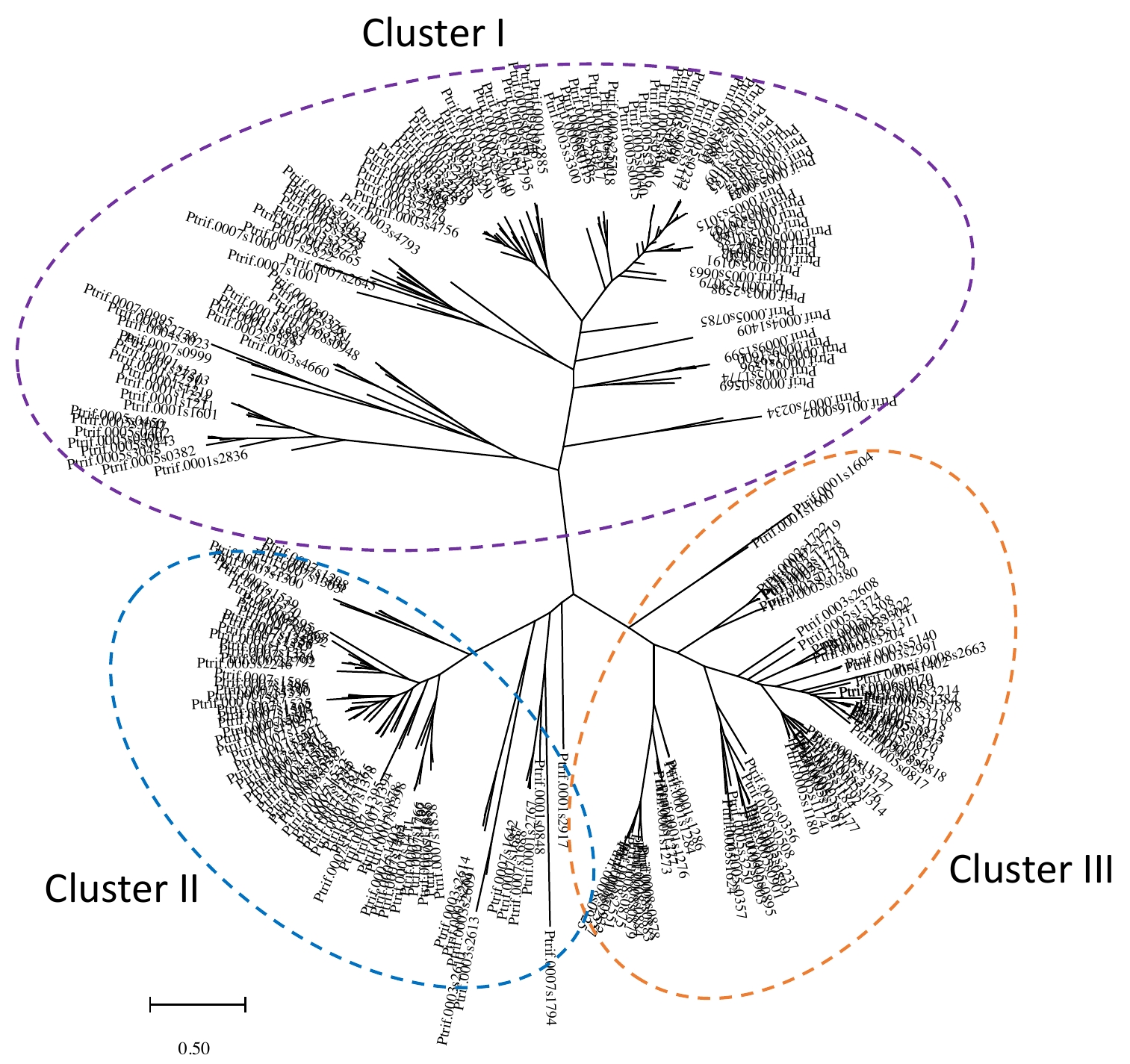

Supplement: Supplementary file 3 — Figure S3. A phylogenetic tree of identified NBS genes in Poncirus trifoliata. Three major clusters were identified (blue, orange and purple dashed lines). [file TPJ-104-1215-s003.jpg]

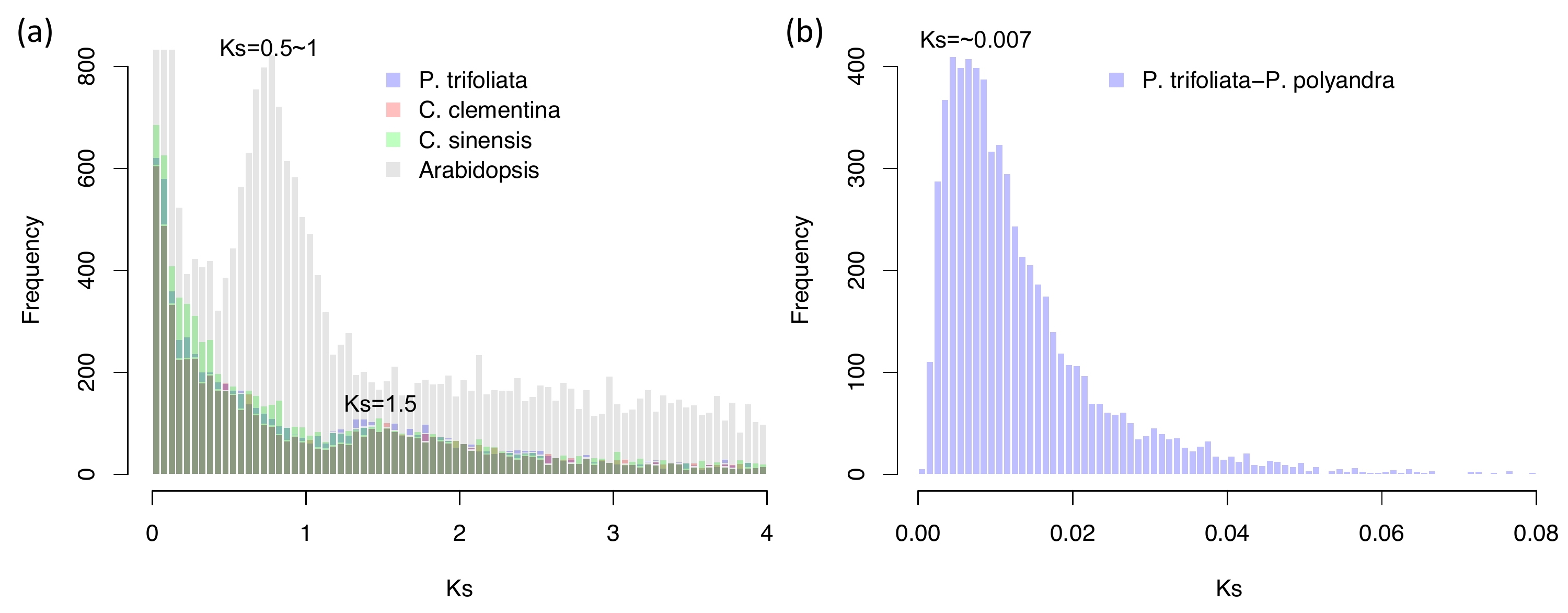

Supplement: Supplementary file 4 — Figure S4. WGD and divergence between Poncirus trifoliata and Poncirus polyandra. (a) The Ks distribution for paralogous gene groups in P. trifoliata, C. × clementina, C. × sinensis and Arabidopsis (as a control). (b) The Ks distribution between P. trifoliata and P. polyandra obtained from the 8326 gene pairs. [file TPJ-104-1215-s004.jpg]

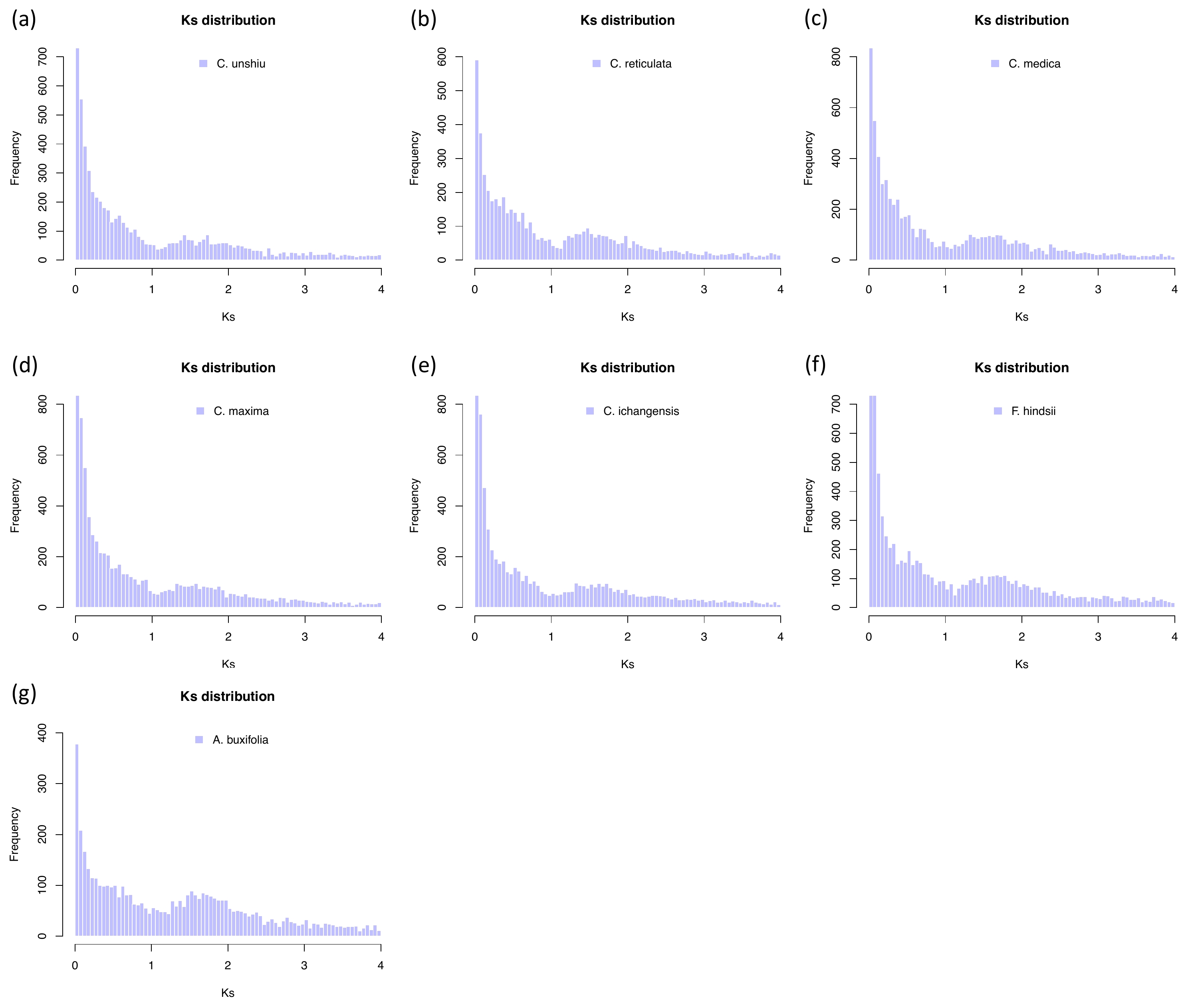

Supplement: Supplementary file 5 — Figure S5. WGD in other Citrus‐related species. [file TPJ-104-1215-s005.jpg]

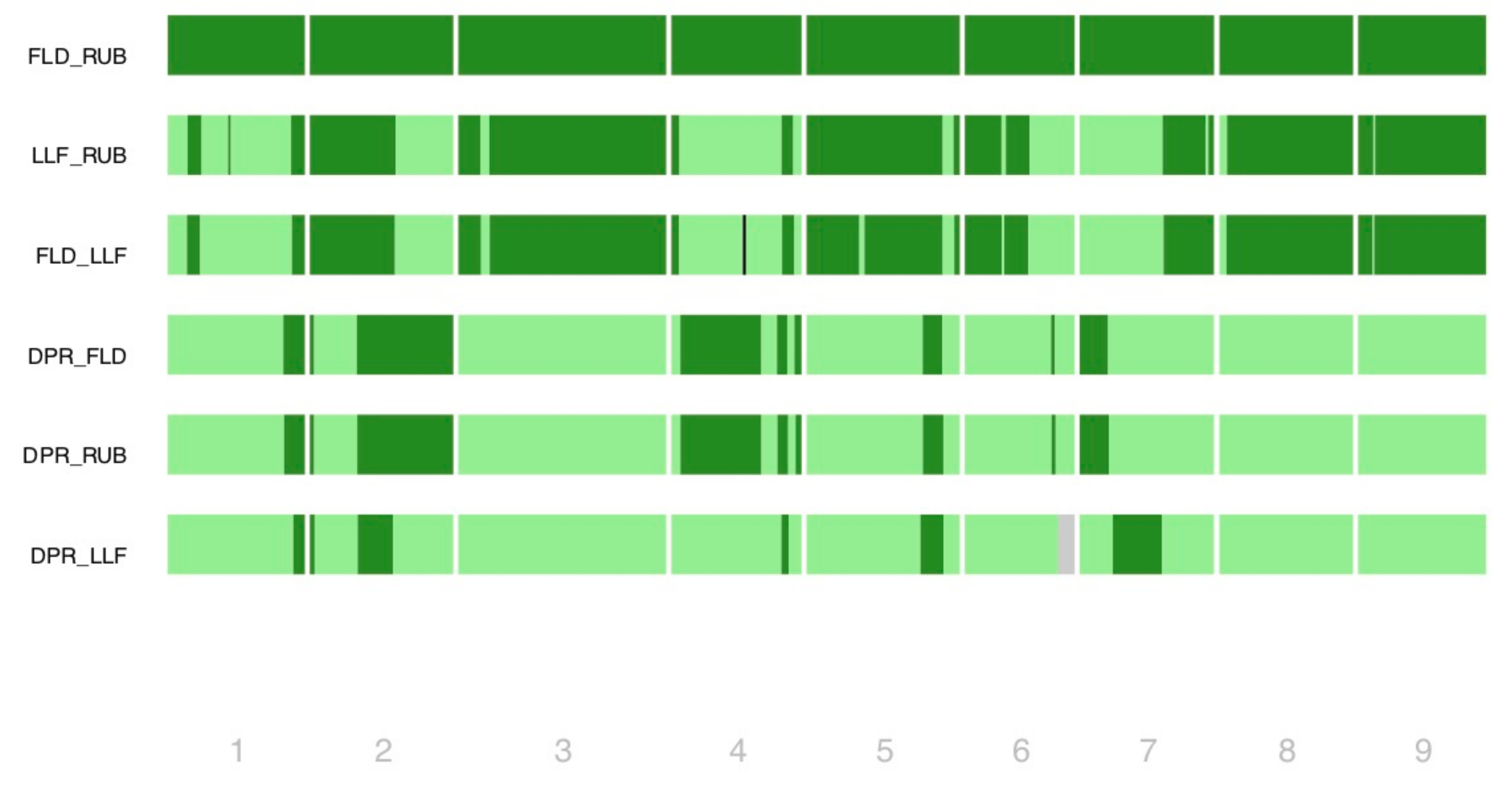

Supplement: Supplementary file 6 — Figure S6. Genetic relatedness among four P. trifoliata accessions. Each row represents one pair of accessions with codes explained in Figure S5(a). Dark and light green colors denote sharing two and one haplotypes, respectively, whereas gray stands for the absence of haplotype sharing. [file TPJ-104-1215-s006.jpg]
