# Supplementary material for: A chromosome‐scale reference genome of trifoliate orange (Poncirus trifoliata) provides insights into disease resistance, cold tolerance and genome evolution in Citrus
Source: Plant J. 2020 Oct 18;104(5):1215–32. doi: 10.1111/tpj.14993 (PMC7756384; doi:10.1111/tpj.14993)
Supplement: Supplementary file 7 — Table S1. Summary of sequenced Poncirus accessions using PacBio and Illumina Hiseq 2500 platforms Table S2. TEs in P. trifoliata genome Table S3. Comparison of completeness and statistics of assemblies among all published Citrus‐related genomes Table S4. Summary of gene families containing cold signaling‐related genes Table S5. Summary of cold signaling‐related genes recovered by re‐annotation Table S6. Classification of identified NBS genes in P. trifoliata Table S7. Summary of identified TF genes in P. trifoliata genome Table S8. Gene family assignment of 10 Citrus‐related species based on existing annotations Table S9. Genes specific to P. trifoliata Table 10. Summary of re‐annotation of single‐copy gene families across 10 genomes Table S11. Summary of rapid evolving gene families in P. trifoliata lineage from CAFÉ analysis Table S12. GO enrichment analysis of P. trifoliata‐specific genes and fast evolving genes in P. trifoliata Table S13. Summary of positively selected genes Table S14. QTLs associated with HLB tolerance mapped to P. trifoliata genome and locations of Ctv and Tyr1 regions Table S15. Candidate P. trifoliata genes responsive to CLas infection and located within QTLs associated with HLB tolerance Table S16. NBS genes located within QTL associated with HLB tolerance and Ctv and Tyr1 regions [file TPJ-104-1215-s007.zip › tpj14993-sup-0007-TableS1-S16.docx]

**Supplementary Tables**

**Table S1.** Summary of sequenced *Poncirus* accessions using PacBio and Illumina Hi-seq 2500 platforms.

| Genotype | Code | Species | Inferred parentage based on sequence analysis | Tolerance to Huanglongbing | Number of reads | Read length | Data size (Gb) | Platform | Estimated genome coverage (×) |
| --- | --- | --- | --- | --- | --- | --- | --- | --- | --- |
| DPI-50-7 | DPI | *Poncirus trifoliata* |  | Highly tolerant | 4953819 | 10874 bp (N50) | 37.1 | PacBio | 140.0 |
| DPI-50-7 | DPI | *Poncirus trifoliata* |  | Highly tolerant | 84802048 (pair) | 125 bp | 21.2 | Hi-seq 2500 | 80.0 |
| Little leaf | LLF | *Poncirus trifoliata* |  | Highly tolerant | 35410958 (pair) | 125 bp | 8.9 | Hi-seq 2500 | 33.4 |
| Flying dragon | FLD | *Poncirus trifoliata* |  | Highly tolerant | 43242390 (pair) | 125 bp | 10.8 | Hi-seq 2500 | 40.8 |
| Rubidoux | RUB | *Poncirus trifoliata* |  | Highly tolerant | 44210721 (pair) | 125 bp | 11.1 | Hi-seq 2500 | 41.7 |
| *Poncirus polyandra* | PCP | *Poncirus polyandra* |  | Unknown | 40409012 (pair) | 125 bp | 10.1 | Hi-seq 2500 | 38.1 |
| Carrizo citrange | PXO | *Citrus x sinensis* x *Poncirus trifoliata* | *Citrus x sinensis* x *Poncirus trifoliata* 'Flying Dragon', 'Rubidoux', or closely related | Tolerant | 45203209 (pair) | 125 bp | 11.3 | Hi-seq 2500 | 42.7 |
| US-897 | FXC | *Citrus reticulata* x *Poncirus trifoliata* | *Citrus reticulata* ‘Cleopatra’ x *Poncirus trifoliata* ‘Flying Dragon’ | Tolerant | 44433566 (pair) | 125 bp | 11.1 | Hi-seq 2500 | 41.9 |
| US802 | PXP | *Citrus grandis* x *Poncirus trifoliata* | Unknown pummelo x *Poncirus trifoliata* ‘Little Leaf’, or closely related | Unknown | 34453114 (pair) | 125 bp | 8.6 | Hi-seq 2500 | 32.5 |
| US-812 | FXS | *Citrus reticulata* x *Poncirus trifoliata* | Unknown madanrin x *Poncirus trifoliata* ‘Little Leaf’, or closely related | Unknown | 44253371 (pair) | 125 bp | 11.1 | Hi-seq 2500 | 41.8 |
| US-942 | FS2 | *Citrus reticulata* x *Poncirus trifoliata* | Unknown mandarin x *Poncirus trifoliata* ‘Flying Dragon’ | Unknown | 49447197 (pair) | 125 bp | 12.4 | Hi-seq 2500 | 46.7 |
| Xcitroncirus X639 | PXC | *Citrus reticulata* x *Poncirus trifoliata* | *Citrus reticulata* ‘Cleopatra’ x *Poncirus trifoliata* 'Little Leaf', or closely related | Unknown | 41005743 (pair) | 125 bp | 10.3 | Hi-seq 2500 | 38.7 |

**Table S2.** Transposable elements in *P. trifoliata* genome.

| TE Classification | Copies (number) | Length (bp) | Percent of genome (%) |
| --- | --- | --- | --- |
| LTR (overall) | 62,675 | 65,250,099 | 24.62 |
| LTR/Gypsy | 19,477 | 24,949,559 | 9.42 |
| LTR/Copia | 17,133 | 16,869,288 | 6.37 |
| LTR/Caulimovirus | 5,381 | 8,454,831 | 3.19 |
| DNA (overall) | 71,231 | 27,564,098 | 10.40 |
| DNA/hAT-Ac | 7,696 | 4689219 | 1.77 |
| DNA/MuDR | 6,199 | 3,163,864 | 1.19 |
| DNA/EnSpm | 2,955 | 1,413,695 | 0.53 |
| DNA/PIF-Harbinger | 3,594 | 1,410,058 | 0.53 |
| DNA/Helitron | 1,495 | 11,856,27 | 0.45 |
| LINE | 13,346 | 6,818,944 | 2.57 |
| SINE | 3,345 | 650,268 | 0.25 |
| Satellites | 157 | 80,068 | 0.03 |
| Simple repeats | 81,806 | 4,189,668 | 1.58 |
| Low complexity | 15,944 | 756,620 | 0.29 |
| Unknown | 24,230 | 7,663,553 | 2.89 |
| Total |  | 112,973,318 | 42.63 |

**Table S3.** Comparison of completeness and statistics of assemblies among all published *Citrus*-related genomes.

| Species name | Version | Complete BUSCOs | | Complete and single-copy BUSCOs | | Complete and duplicated BUSCOs | | Fragmented BUSCOs | | Missing BUSCOs | | Total BUSCO groups searched | Total size of assembly (bp) | No. of scaffolds/contigs | Longest scaffolds/contigs (bp) | N50 (bp) | No. of gene models |
| --- | --- | --- | --- | --- | --- | --- | --- | --- | --- | --- | --- | --- | --- | --- | --- | --- | --- |
|  |  | Number | Percentage | Number | Percentage | Number | Percentage | Number | Percentage | Number | Percentage |  |  |  |  |  |  |
| *Poncirus trifoliata* | V1.3 | 1399 | 97.20% | 1363 | 94.70% | 36 | 2.50% | 15 | 1.00% | 26 | 1.80% | 1440 | 264980413 | 152 | 42674353 | 27685527 | 25538 |
| *Fortunella hindsii* | V1.0 | 1373 | 95.40% | 1084 | 75.30% | 289 | 20.10% | 25 | 1.70% | 42 | 2.90% | 1440 | 373557569 | 1226 | 11998049 | 2209464 | 32257 |
| *Citrus x clementina* | V1.0 | 1366 | 94.90% | 1329 | 92.30% | 37 | 2.60% | 36 | 2.50% | 38 | 2.60% | 1440 | 301386998 | 1398 | 51050279 | 31410901 | 24533 |
| *Citrus maxima* | V1.0 | 1359 | 94.40% | 1313 | 91.20% | 46 | 3.20% | 38 | 2.60% | 43 | 3.00% | 1440 | 345779982 | 10 | 53007170 | 40395599 | 30123 |
| *Citrus x sinensis* (Huazhong) | V2.0 | 1355 | 94.10% | 1310 | 91.00% | 45 | 3.10% | 37 | 2.60% | 48 | 3.30% | 1440 | 327944670 | 10 | 88947451 | 30837053 | 29406 |
| *Citrus x sinensis* (JGI) | V1.1 | 1255 | 87.20% | 1221 | 84.80% | 34 | 2.40% | 94 | 6.50% | 91 | 6.30% | 1440 | 319231331 | 12574 | 5927163 | 250548 | 25379 |
| *Atalantia buxifolia* | V1.0 | 1352 | 93.90% | 1298 | 90.10% | 54 | 3.80% | 53 | 3.70% | 35 | 2.40% | 1440 | 315820821 | 25600 | 7163423 | 1073988 | 28412 |
| *Citrus medica* | V1.0 | 1348 | 93.60% | 1231 | 85.50% | 117 | 8.10% | 33 | 2.30% | 59 | 4.10% | 1440 | 406057947 | 32732 | 2443612 | 369527 | 32579 |
| *Citrus reticulata* | V1.0 | 1345 | 93.40% | 1309 | 90.90% | 36 | 2.50% | 49 | 3.40% | 46 | 3.20% | 1440 | 347457508 | 90139 | 8970695 | 1585532 | 28833 |
| *Citrus unshiu* | V1.0 | 1324 | 92.00% | 1208 | 83.90% | 116 | 8.10% | 63 | 4.40% | 53 | 3.60% | 1440 | 359652061 | 20876 | 5227725 | 386404 | 29024 |
| *Citrus ichangensis* | V1.0 | 1301 | 90.30% | 1207 | 83.80% | 94 | 6.50% | 34 | 2.40% | 105 | 7.30% | 1440 | 357621246 | 14916 | 2984400 | 501435 | 32067 |

Note: Dataset "embryophyta_odb9"; BUSCO was run in mode: proteins. The longest isoform was used for genes with multiple isoforms.

**Table S4.** Summary of gene families containing cold signaling-related genes.

Table S4 is submitted separately due to a large size.

**Table S5.** Summary of cold signaling-related genes recovered by re-annotation.

| Species | Percentage of gene families recovered by re-annotation | | Percentage of genes recovered by re-annotation | |
| --- | --- | --- | --- | --- |
|  | Overall (96 gene families) | Low-copy (less than 2 genes for each species; 76 gene families) | Overall (96 gene families) | Low-copy (less than 2 genes for each species; 76 gene families) |
| *P. trifoliata* | 91.67% | 96.05% | 90.78% | 96.30% |
| *F. hindsii* | 90.63% | 92.11% | 93.37% | 93.33% |
| *C. ichangensis* | 92.71% | 94.74% | 95.03% | 94.81% |
| *C. sinensis* | 91.67% | 94.74% | 93.48% | 94.87% |
| *C. maxima* | 90.63% | 92.11% | 93.55% | 92.31% |
| *C. medica* | 86.46% | 90.79% | 84.73% | 91.25% |
| *C. reticulata* | 89.58% | 93.42% | 91.72% | 93.98% |
| *C. unshiu* | 76.04% | 86.84% | 76.26% | 86.90% |
| *C. x clementina* | 89.58% | 93.42% | 79.64% | 93.83% |
| *A. buxifolia* | 88.54% | 92.11% | 90.85% | 91.36% |
| Average | 88.75% | 92.63% | 88.94% | 92.89% |

**Table S6.** Classification of identified NBS genes in *P. trifoliata*.

| Gene ID | Class |
| --- | --- |
| Ptrif.0001s0848.1 | CC-NBS-LRR |
| Ptrif.0001s0878.1 | NBS-LRR |
| Ptrif.0001s1190.1 | NBS |
| Ptrif.0001s1208.1 | NBS |
| Ptrif.0001s1211.2 | NBS |
| Ptrif.0001s1213.1 | CC-NBS |
| Ptrif.0001s1219.1 | NBS |
| Ptrif.0001s1221.1 | CC-NBS |
| Ptrif.0001s1224.1 | NBS |
| Ptrif.0001s1229.2 | NBS |
| Ptrif.0001s1273.1 | CC-NBS |
| Ptrif.0001s1276.2 | CC-NBS |
| Ptrif.0001s1277.2 | CC-NBS |
| Ptrif.0001s1284.2 | CC-NBS |
| Ptrif.0001s1286.1 | CC-NBS |
| Ptrif.0001s1600.1 | CC-NBS-LRR |
| Ptrif.0001s1601.1 | NBS |
| Ptrif.0001s1604.2 | CC-NBS-LRR |
| Ptrif.0001s1609.1 | CC-NBS |
| Ptrif.0001s1883.2 | NBS-LRR |
| Ptrif.0001s1884.1 | NBS-LRR |
| Ptrif.0001s1924.1 | TIR-NBS |
| Ptrif.0001s2750.1 | CC-NBS |
| Ptrif.0001s2767.1 | NBS-LRR |
| Ptrif.0001s2795.1 | NBS |
| Ptrif.0001s2836.1 | NBS |
| Ptrif.0001s2878.1 | NBS |
| Ptrif.0001s2885.1 | CC-NBS |
| Ptrif.0001s2917.1 | CC-NBS-LRR |
| Ptrif.0001s2967.1 | CC-NBS |
| Ptrif.0002s0342.2 | CC-NBS |
| Ptrif.0002s0376.1 | NBS |
| Ptrif.0002s0420.1 | TIR-NBS |
| Ptrif.0002s1714.3 | CC-NBS |
| Ptrif.0002s1718.2 | CC-NBS |
| Ptrif.0002s1719.1 | CC-NBS-LRR |
| Ptrif.0002s1722.1 | CC-NBS-LRR |
| Ptrif.0002s1724.2 | CC-NBS |
| Ptrif.0002s3281.1 | NBS |
| Ptrif.0002s3381.1 | NBS |
| Ptrif.0003s1300.2 | TIR-NBS |
| Ptrif.0003s1302.1 | TIR-NBS |
| Ptrif.0003s1914.2 | TIR-NBS |
| Ptrif.0003s2075.1 | TIR-NBS |
| Ptrif.0003s2114.1 | TIR-NBS |
| Ptrif.0003s2127.1 | NBS |
| Ptrif.0003s2149.1 | TIR-NBS |
| Ptrif.0003s2152.1 | TIR-NBS |
| Ptrif.0003s2153.1 | TIR-NBS |
| Ptrif.0003s2156.1 | TIR-NBS |
| Ptrif.0003s2157.1 | TIR-NBS |
| Ptrif.0003s2158.4 | TIR-NBS |
| Ptrif.0003s2160.2 | TIR-NBS-LRR |
| Ptrif.0003s2188.1 | TIR-NBS |
| Ptrif.0003s2190.2 | TIR-NBS |
| Ptrif.0003s2194.1 | TIR-NBS |
| Ptrif.0003s2197.1 | TIR-NBS |
| Ptrif.0003s2198.1 | NBS |
| Ptrif.0003s2204.3 | TIR-NBS-LRR |
| Ptrif.0003s2206.2 | TIR-NBS |
| Ptrif.0003s2207.1 | TIR-NBS |
| Ptrif.0003s2208.1 | TIR-NBS-LRR |
| Ptrif.0003s2213.1 | TIR-NBS |
| Ptrif.0003s2218.1 | TIR-NBS |
| Ptrif.0003s2224.2 | TIR-NBS-LRR |
| Ptrif.0003s2235.1 | TIR-NBS-LRR |
| Ptrif.0003s2237.1 | TIR-NBS-LRR |
| Ptrif.0003s2240.2 | TIR-NBS |
| Ptrif.0003s2241.2 | TIR-NBS |
| Ptrif.0003s2246.1 | NBS-LRR |
| Ptrif.0003s2326.1 | TIR-NBS |
| Ptrif.0003s2329.4 | TIR-NBS |
| Ptrif.0003s2331.1 | TIR-NBS |
| Ptrif.0003s2332.1 | TIR-CC-NBS |
| Ptrif.0003s2334.1 | NBS |
| Ptrif.0003s2337.1 | TIR-CC-NBS |
| Ptrif.0003s2389.1 | NBS-LRR |
| Ptrif.0003s2390.1 | NBS |
| Ptrif.0003s2391.3 | NBS |
| Ptrif.0003s2398.1 | NBS-LRR |
| Ptrif.0003s2405.1 | NBS |
| Ptrif.0003s2407.1 | CC-NBS |
| Ptrif.0003s2408.1 | NBS |
| Ptrif.0003s2410.2 | NBS-LRR |
| Ptrif.0003s2419.2 | NBS-LRR |
| Ptrif.0003s2420.1 | NBS |
| Ptrif.0003s2422.2 | NBS |
| Ptrif.0003s2429.2 | NBS-LRR |
| Ptrif.0003s2473.1 | CC-NBS-LRR |
| Ptrif.0003s2475.2 | NBS-LRR |
| Ptrif.0003s2477.1 | NBS-LRR |
| Ptrif.0003s2479.1 | NBS-LRR |
| Ptrif.0003s2485.1 | NBS-LRR |
| Ptrif.0003s2486.1 | NBS-LRR |
| Ptrif.0003s2489.2 | NBS |
| Ptrif.0003s2561.1 | NBS |
| Ptrif.0003s2570.1 | NBS |
| Ptrif.0003s2598.1 | CC-NBS |
| Ptrif.0003s2608.1 | CC-NBS-LRR |
| Ptrif.0003s2609.1 | TIR-NBS-LRR |
| Ptrif.0003s2610.1 | NBS-LRR |
| Ptrif.0003s2611.1 | NBS-LRR |
| Ptrif.0003s2612.1 | NBS |
| Ptrif.0003s2613.1 | NBS-LRR |
| Ptrif.0003s2614.1 | NBS-LRR |
| Ptrif.0003s2769.2 | CC-NBS |
| Ptrif.0003s2807.1 | TIR-NBS-LRR |
| Ptrif.0003s2991.2 | CC-NBS-LRR |
| Ptrif.0003s3300.1 | NBS |
| Ptrif.0003s4660.1 | NBS |
| Ptrif.0003s4680.1 | TIR-NBS |
| Ptrif.0003s4702.1 | TIR-NBS |
| Ptrif.0003s4705.1 | TIR-NBS |
| Ptrif.0003s4718.1 | NBS |
| Ptrif.0003s4723.1 | TIR-NBS |
| Ptrif.0003s4756.1 | NBS |
| Ptrif.0003s4772.1 | TIR-NBS |
| Ptrif.0003s4775.1 | TIR-NBS |
| Ptrif.0003s4780.1 | TIR-NBS |
| Ptrif.0003s4782.1 | NBS |
| Ptrif.0003s4793.1 | NBS |
| Ptrif.0003s4806.1 | TIR-NBS |
| Ptrif.0003s4816.1 | TIR-NBS |
| Ptrif.0003s4840.1 | CC-NBS |
| Ptrif.0003s4848.1 | NBS |
| Ptrif.0003s4869.1 | NBS-LRR |
| Ptrif.0003s4890.1 | NBS |
| Ptrif.0003s4916.1 | TIR-NBS |
| Ptrif.0003s4953.1 | TIR-NBS |
| Ptrif.0003s5140.1 | CC-NBS-LRR |
| Ptrif.0004s1409.1 | CC-NBS-LRR |
| Ptrif.0004s1503.1 | NBS-LRR |
| Ptrif.0004s1505.1 | NBS-LRR |
| Ptrif.0004s1508.1 | NBS-LRR |
| Ptrif.0004s1532.1 | CC-NBS |
| Ptrif.0004s2276.2 | TIR-NBS |
| Ptrif.0004s3023.1 | NBS-LRR |
| Ptrif.0005s0015.1 | NBS |
| Ptrif.0005s0030.1 | NBS |
| Ptrif.0005s0031.1 | NBS-LRR |
| Ptrif.0005s0032.2 | NBS-LRR |
| Ptrif.0005s0037.1 | CC-NBS |
| Ptrif.0005s0040.1 | NBS |
| Ptrif.0005s0042.1 | CC-NBS |
| Ptrif.0005s0043.1 | NBS |
| Ptrif.0005s0072.2 | NBS |
| Ptrif.0005s0075.1 | CC-NBS |
| Ptrif.0005s0084.1 | NBS |
| Ptrif.0005s0090.2 | CC-NBS |
| Ptrif.0005s0175.1 | NBS-LRR |
| Ptrif.0005s0181.1 | NBS |
| Ptrif.0005s0188.2 | NBS |
| Ptrif.0005s0191.2 | NBS |
| Ptrif.0005s0220.1 | CC-NBS |
| Ptrif.0005s0232.1 | NBS |
| Ptrif.0005s0250.1 | NBS-LRR |
| Ptrif.0005s0356.1 | CC-NBS |
| Ptrif.0005s0357.1 | CC-NBS-LRR |
| Ptrif.0005s0379.2 | CC-NBS-LRR |
| Ptrif.0005s0380.1 | CC-NBS-LRR |
| Ptrif.0005s0382.2 | NBS-LRR |
| Ptrif.0005s0400.1 | NBS |
| Ptrif.0005s0402.2 | NBS |
| Ptrif.0005s0443.1 | NBS-LRR |
| Ptrif.0005s0450.2 | NBS |
| Ptrif.0005s0469.1 | NBS |
| Ptrif.0005s0471.1 | NBS |
| Ptrif.0005s0641.1 | NBS |
| Ptrif.0005s0663.1 | NBS |
| Ptrif.0005s0785.1 | NBS-LRR |
| Ptrif.0005s0813.1 | CC-NBS-LRR |
| Ptrif.0005s0815.2 | CC-NBS-LRR |
| Ptrif.0005s0817.1 | CC-NBS-LRR |
| Ptrif.0005s0818.1 | CC-NBS |
| Ptrif.0005s0820.1 | CC-NBS |
| Ptrif.0005s0823.1 | CC-NBS |
| Ptrif.0005s0895.1 | NBS |
| Ptrif.0005s0899.2 | NBS-LRR |
| Ptrif.0005s0901.1 | NBS-LRR |
| Ptrif.0005s0903.1 | NBS |
| Ptrif.0005s1172.1 | CC-NBS |
| Ptrif.0005s1174.1 | CC-NBS |
| Ptrif.0005s1177.1 | CC-NBS-LRR |
| Ptrif.0005s1180.1 | CC-NBS-LRR |
| Ptrif.0005s1181.1 | CC-NBS-LRR |
| Ptrif.0005s1184.1 | CC-NBS-LRR |
| Ptrif.0005s1191.1 | CC-NBS-LRR |
| Ptrif.0005s1192.2 | CC-NBS-LRR |
| Ptrif.0005s1308.1 | CC-NBS |
| Ptrif.0005s1311.2 | CC-NBS |
| Ptrif.0005s1314.2 | CC-NBS-LRR |
| Ptrif.0005s1315.1 | CC-NBS |
| Ptrif.0005s1322.1 | NBS |
| Ptrif.0005s1374.1 | CC-NBS |
| Ptrif.0005s1378.2 | CC-NBS-LRR |
| Ptrif.0005s1380.2 | NBS-LRR |
| Ptrif.0005s1384.1 | CC-NBS |
| Ptrif.0005s1402.1 | CC-NBS |
| Ptrif.0005s1624.1 | CC-NBS |
| Ptrif.0005s1774.1 | NBS |
| Ptrif.0005s2548.1 | NBS |
| Ptrif.0005s2985.1 | NBS |
| Ptrif.0005s2990.1 | NBS |
| Ptrif.0005s2993.1 | NBS |
| Ptrif.0005s3010.1 | CC-NBS |
| Ptrif.0005s3015.1 | CC-NBS |
| Ptrif.0005s3016.1 | CC-NBS |
| Ptrif.0005s3032.1 | NBS |
| Ptrif.0005s3047.1 | NBS-LRR |
| Ptrif.0005s3048.1 | NBS-LRR |
| Ptrif.0005s3050.1 | TIR-NBS |
| Ptrif.0005s3071.1 | CC-NBS-LRR |
| Ptrif.0005s3079.1 | NBS |
| Ptrif.0005s3085.1 | CC-NBS |
| Ptrif.0005s3109.1 | NBS |
| Ptrif.0005s3117.1 | CC-NBS |
| Ptrif.0005s3118.1 | CC-NBS-LRR |
| Ptrif.0005s3176.1 | CC-NBS |
| Ptrif.0005s3177.1 | CC-NBS |
| Ptrif.0005s3192.1 | CC-NBS |
| Ptrif.0005s3204.1 | NBS-LRR |
| Ptrif.0005s3205.1 | CC-NBS |
| Ptrif.0005s3213.1 | CC-NBS |
| Ptrif.0005s3214.1 | CC-NBS-LRR |
| Ptrif.0005s3218.1 | CC-NBS-LRR |
| Ptrif.0005s3222.1 | CC-NBS |
| Ptrif.0005s3224.1 | NBS-LRR |
| Ptrif.0005s3228.1 | CC-NBS |
| Ptrif.0005s3237.1 | CC-NBS |
| Ptrif.0005s3250.1 | CC-NBS-LRR |
| Ptrif.0005s3304.1 | CC-NBS |
| Ptrif.0005s3525.1 | NBS-LRR |
| Ptrif.0006s0069.1 | CC-NBS |
| Ptrif.0006s0070.2 | CC-NBS-LRR |
| Ptrif.0006s0113.1 | CC-NBS-LRR |
| Ptrif.0006s0115.1 | NBS |
| Ptrif.0006s0117.2 | CC-NBS |
| Ptrif.0006s0118.1 | CC-NBS-LRR |
| Ptrif.0006s0508.1 | CC-NBS |
| Ptrif.0006s1501.1 | CC-NBS |
| Ptrif.0006s2286.1 | CC-NBS |
| Ptrif.0007s0234.1 | NBS-LRR |
| Ptrif.0007s0387.1 | CC-NBS |
| Ptrif.0007s0995.1 | NBS |
| Ptrif.0007s0999.1 | CC-NBS-LRR |
| Ptrif.0007s1000.1 | NBS |
| Ptrif.0007s1001.1 | CC-NBS-LRR |
| Ptrif.0007s1130.1 | NBS |
| Ptrif.0007s1212.1 | TIR-NBS |
| Ptrif.0007s1217.2 | TIR-NBS |
| Ptrif.0007s1298.1 | CC-NBS-LRR |
| Ptrif.0007s1300.2 | CC-NBS-LRR |
| Ptrif.0007s1301.1 | CC-NBS-LRR |
| Ptrif.0007s1305.1 | CC-NBS-LRR |
| Ptrif.0007s1354.1 | CC-NBS-LRR |
| Ptrif.0007s1355.1 | CC-NBS |
| Ptrif.0007s1356.1 | CC-NBS-LRR |
| Ptrif.0007s1357.1 | NBS-LRR |
| Ptrif.0007s1358.2 | CC-NBS-LRR |
| Ptrif.0007s1360.1 | CC-NBS-LRR |
| Ptrif.0007s1363.1 | CC-NBS-LRR |
| Ptrif.0007s1365.1 | CC-NBS |
| Ptrif.0007s1367.1 | CC-NBS-LRR |
| Ptrif.0007s1370.1 | NBS-LRR |
| Ptrif.0007s1378.1 | CC-NBS-LRR |
| Ptrif.0007s1394.1 | CC-NBS-LRR |
| Ptrif.0007s1395.1 | CC-NBS-LRR |
| Ptrif.0007s1396.1 | NBS |
| Ptrif.0007s1398.1 | CC-NBS-LRR |
| Ptrif.0007s1402.1 | CC-NBS-LRR |
| Ptrif.0007s1404.1 | CC-NBS |
| Ptrif.0007s1406.2 | NBS-LRR |
| Ptrif.0007s1411.2 | CC-NBS-LRR |
| Ptrif.0007s1415.1 | CC-NBS-LRR |
| Ptrif.0007s1481.1 | NBS |
| Ptrif.0007s1484.1 | CC-NBS-LRR |
| Ptrif.0007s1501.1 | CC-NBS-LRR |
| Ptrif.0007s1502.1 | CC-NBS-LRR |
| Ptrif.0007s1503.1 | CC-NBS-LRR |
| Ptrif.0007s1509.1 | CC-NBS-LRR |
| Ptrif.0007s1511.1 | CC-NBS |
| Ptrif.0007s1514.1 | CC-NBS-LRR |
| Ptrif.0007s1515.1 | CC-NBS |
| Ptrif.0007s1518.1 | CC-NBS |
| Ptrif.0007s1522.1 | CC-NBS-LRR |
| Ptrif.0007s1527.1 | CC-NBS-LRR |
| Ptrif.0007s1529.2 | NBS-LRR |
| Ptrif.0007s1530.1 | CC-NBS-LRR |
| Ptrif.0007s1532.1 | CC-NBS-LRR |
| Ptrif.0007s1533.1 | CC-NBS-LRR |
| Ptrif.0007s1534.1 | CC-NBS-LRR |
| Ptrif.0007s1535.1 | CC-NBS-LRR |
| Ptrif.0007s1539.1 | CC-NBS-LRR |
| Ptrif.0007s1586.1 | CC-NBS-LRR |
| Ptrif.0007s1587.1 | NBS-LRR |
| Ptrif.0007s1590.1 | CC-NBS-LRR |
| Ptrif.0007s1595.1 | CC-NBS-LRR |
| Ptrif.0007s1642.2 | NBS-LRR |
| Ptrif.0007s1687.1 | CC-NBS-LRR |
| Ptrif.0007s1688.1 | CC-NBS-LRR |
| Ptrif.0007s1766.1 | NBS |
| Ptrif.0007s1767.1 | NBS-LRR |
| Ptrif.0007s1768.1 | CC-NBS-LRR |
| Ptrif.0007s1794.2 | NBS-LRR |
| Ptrif.0007s1836.1 | CC-NBS |
| Ptrif.0007s1837.2 | CC-NBS-LRR |
| Ptrif.0007s1838.1 | NBS |
| Ptrif.0007s2331.2 | CC-NBS |
| Ptrif.0007s2645.1 | CC-NBS |
| Ptrif.0007s2665.1 | NBS |
| Ptrif.0007s2692.1 | CC-NBS |
| Ptrif.0007s2695.1 | CC-NBS |
| Ptrif.0007s2703.1 | CC-NBS |
| Ptrif.0007s2792.1 | CC-NBS-LRR |
| Ptrif.0007s2822.1 | CC-NBS |
| Ptrif.0008s0569.2 | CC-NBS |
| Ptrif.0008s0582.2 | CC-NBS-LRR |
| Ptrif.0008s0875.4 | CC-NBS |
| Ptrif.0008s0878.1 | CC-NBS |
| Ptrif.0008s0879.1 | CC-NBS |
| Ptrif.0008s0883.1 | CC-NBS |
| Ptrif.0008s0884.2 | CC-NBS |
| Ptrif.0008s0925.2 | CC-NBS |
| Ptrif.0008s0932.2 | CC-NBS |
| Ptrif.0008s0948.1 | NBS |
| Ptrif.0008s2520.1 | CC-NBS-LRR |
| Ptrif.0008s2551.1 | CC-NBS |
| Ptrif.0008s2557.1 | CC-NBS |
| Ptrif.0008s2663.1 | CC-NBS-LRR |
| Ptrif.0008s2738.1 | NBS-LRR |
| Ptrif.0008s2764.1 | CC-NBS |
| Ptrif.0009s0943.1 | NBS |
| Ptrif.0009s0944.1 | NBS-LRR |
| Ptrif.0009s0946.2 | NBS |
| Ptrif.0009s1023.1 | TIR-NBS |
| Ptrif.0009s1449.1 | TIR-NBS |
| Ptrif.0009s1451.1 | TIR-NBS |
| Ptrif.0009s1453.1 | TIR-NBS |
| Ptrif.0009s1458.2 | TIR-NBS |
| Ptrif.0009s1595.1 | NBS |
| Ptrif.0009s1596.1 | NBS |
| Ptrif.0009s1599.1 | CC-NBS |
| Ptrif.0009s1600.1 | NBS |
| Ptrif.0009s1735.1 | NBS |
| Ptrif.0009s2497.1 | CC-NBS |
| Ptrif.0009s2550.1 | TIR-NBS |
| Ptrif.0009s2650.1 | TIR-NBS |
| Ptrif.0016s0007.1 | NBS-LRR |

**Table S7.** Summary of identified transcription factor genes in *P. trifoliata* genome.

| Transcription factor family | No. of genes |
| --- | --- |
| bHLH | 124 |
| NAC | 123 |
| ERF | 110 |
| MYB | 109 |
| C2H2 | 82 |
| FAR1 | 89 |
| B3 | 54 |
| WRKY | 53 |
| bZIP | 53 |
| MYB_related | 53 |
| M-type_MADS | 50 |
| GRAS | 45 |
| LBD | 35 |
| C3H | 41 |
| G2-like | 35 |
| HD-ZIP | 34 |
| Trihelix | 32 |
| MIKC_MADS | 35 |
| GATA | 26 |
| Dof | 24 |
| HSF | 19 |
| AP2 | 17 |
| TALE | 20 |
| TCP | 20 |
| NF-YB | 13 |
| ARF | 18 |
| SBP | 14 |
| WOX | 11 |
| ZF-HD | 12 |
| ARR-B | 10 |
| DBB | 6 |
| NF-YC | 9 |
| BES1 | 8 |
| GRF | 10 |
| Nin-like | 8 |
| GeBP | 7 |
| HB-other | 7 |
| YABBY | 7 |
| CO-like | 9 |
| CPP | 7 |
| E2F/DP | 6 |
| NF-YA | 6 |
| EIL | 5 |
| SRS | 5 |
| CAMTA | 6 |
| LSD | 3 |
| RAV | 4 |
| BBR-BPC | 3 |
| HB-PHD | 2 |
| HRT-like | 2 |
| NF-X1 | 2 |
| NZZ/SPL | 2 |
| VOZ | 2 |
| Whirly | 2 |
| LFY | 1 |
| S1Fa-like | 1 |
| SAP | 1 |
| STAT | 1 |
| Total | 1493 |

**Table S8.** Gene family assignment of 10 *Citrus*-related species based on existing annotations.

Table S8 is submitted separately due to a large size.

**Table S9.** Genes specific to *Poncirus trifoliata*.

| Gene ID | Type |
| --- | --- |
| Ptrif.0001s0069.1 | Unclustered genes of *Poncirus trifoliata* |
| Ptrif.0001s0251.1 | Unclustered genes of *Poncirus trifoliata* |
| Ptrif.0001s0475.1 | Unclustered genes of *Poncirus trifoliata* |
| Ptrif.0001s0498.1 | Unclustered genes of *Poncirus trifoliata* |
| Ptrif.0001s0501.1 | Unclustered genes of *Poncirus trifoliata* |
| Ptrif.0001s0723.1 | Unclustered genes of *Poncirus trifoliata* |
| Ptrif.0001s0854.2 | Unclustered genes of *Poncirus trifoliata* |
| Ptrif.0001s0937.1 | Unclustered genes of *Poncirus trifoliata* |
| Ptrif.0001s0946.1 | Unclustered genes of *Poncirus trifoliata* |
| Ptrif.0001s1095.1 | Unclustered genes of *Poncirus trifoliata* |
| Ptrif.0001s1145.1 | Unclustered genes of *Poncirus trifoliata* |
| Ptrif.0001s1170.1 | Unclustered genes of *Poncirus trifoliata* |
| Ptrif.0001s1240.1 | Unclustered genes of *Poncirus trifoliata* |
| Ptrif.0001s1246.2 | Unclustered genes of *Poncirus trifoliata* |
| Ptrif.0001s1352.1 | Unclustered genes of *Poncirus trifoliata* |
| Ptrif.0001s1391.1 | Unclustered genes of *Poncirus trifoliata* |
| Ptrif.0001s1506.1 | Unclustered genes of *Poncirus trifoliata* |
| Ptrif.0001s1606.1 | Unclustered genes of *Poncirus trifoliata* |
| Ptrif.0001s1679.2 | Unclustered genes of *Poncirus trifoliata* |
| Ptrif.0001s1709.1 | Unclustered genes of *Poncirus trifoliata* |
| Ptrif.0001s1809.1 | Unclustered genes of *Poncirus trifoliata* |
| Ptrif.0001s1810.1 | Unclustered genes of *Poncirus trifoliata* |
| Ptrif.0001s1823.1 | Unclustered genes of *Poncirus trifoliata* |
| Ptrif.0001s1930.1 | Unclustered genes of *Poncirus trifoliata* |
| Ptrif.0001s2112.2 | Unclustered genes of *Poncirus trifoliata* |
| Ptrif.0001s2235.1 | Unclustered genes of *Poncirus trifoliata* |
| Ptrif.0001s2456.2 | Unclustered genes of *Poncirus trifoliata* |
| Ptrif.0001s2457.1 | Unclustered genes of *Poncirus trifoliata* |
| Ptrif.0001s2647.1 | Unclustered genes of *Poncirus trifoliata* |
| Ptrif.0001s2652.1 | Unclustered genes of *Poncirus trifoliata* |
| Ptrif.0001s2653.1 | Unclustered genes of *Poncirus trifoliata* |
| Ptrif.0001s2663.1 | Unclustered genes of *Poncirus trifoliata* |
| Ptrif.0001s2679.1 | Unclustered genes of *Poncirus trifoliata* |
| Ptrif.0001s2680.1 | Unclustered genes of *Poncirus trifoliata* |
| Ptrif.0001s2686.1 | Unclustered genes of *Poncirus trifoliata* |
| Ptrif.0001s2697.1 | Unclustered genes of *Poncirus trifoliata* |
| Ptrif.0001s2706.1 | Unclustered genes of *Poncirus trifoliata* |
| Ptrif.0001s2708.1 | Unclustered genes of *Poncirus trifoliata* |
| Ptrif.0001s2725.1 | Unclustered genes of *Poncirus trifoliata* |
| Ptrif.0001s2728.1 | Unclustered genes of *Poncirus trifoliata* |
| Ptrif.0001s2731.1 | Unclustered genes of *Poncirus trifoliata* |
| Ptrif.0001s2732.1 | Unclustered genes of *Poncirus trifoliata* |
| Ptrif.0001s2736.1 | Unclustered genes of *Poncirus trifoliata* |
| Ptrif.0001s2752.1 | Unclustered genes of *Poncirus trifoliata* |
| Ptrif.0001s2760.1 | Unclustered genes of *Poncirus trifoliata* |
| Ptrif.0001s2803.1 | Unclustered genes of *Poncirus trifoliata* |
| Ptrif.0001s2804.1 | Unclustered genes of *Poncirus trifoliata* |
| Ptrif.0001s2824.1 | Unclustered genes of *Poncirus trifoliata* |
| Ptrif.0001s2830.1 | Unclustered genes of *Poncirus trifoliata* |
| Ptrif.0001s2832.1 | Unclustered genes of *Poncirus trifoliata* |
| Ptrif.0001s2845.1 | Unclustered genes of *Poncirus trifoliata* |
| Ptrif.0001s2847.1 | Unclustered genes of *Poncirus trifoliata* |
| Ptrif.0001s2891.1 | Unclustered genes of *Poncirus trifoliata* |
| Ptrif.0002s0119.2 | Unclustered genes of *Poncirus trifoliata* |
| Ptrif.0002s0201.1 | Unclustered genes of *Poncirus trifoliata* |
| Ptrif.0002s0221.1 | Unclustered genes of *Poncirus trifoliata* |
| Ptrif.0002s0317.2 | Unclustered genes of *Poncirus trifoliata* |
| Ptrif.0002s0323.1 | Unclustered genes of *Poncirus trifoliata* |
| Ptrif.0002s0326.2 | Unclustered genes of *Poncirus trifoliata* |
| Ptrif.0002s0327.1 | Unclustered genes of *Poncirus trifoliata* |
| Ptrif.0002s0527.2 | Unclustered genes of *Poncirus trifoliata* |
| Ptrif.0002s0570.1 | Unclustered genes of *Poncirus trifoliata* |
| Ptrif.0002s0708.1 | Unclustered genes of *Poncirus trifoliata* |
| Ptrif.0002s0709.1 | Unclustered genes of *Poncirus trifoliata* |
| Ptrif.0002s0845.1 | Unclustered genes of *Poncirus trifoliata* |
| Ptrif.0002s0855.2 | Unclustered genes of *Poncirus trifoliata* |
| Ptrif.0002s0877.1 | Unclustered genes of *Poncirus trifoliata* |
| Ptrif.0002s0889.1 | Unclustered genes of *Poncirus trifoliata* |
| Ptrif.0002s0942.1 | Unclustered genes of *Poncirus trifoliata* |
| Ptrif.0002s0960.1 | Unclustered genes of *Poncirus trifoliata* |
| Ptrif.0002s1027.1 | Unclustered genes of *Poncirus trifoliata* |
| Ptrif.0002s1148.2 | Unclustered genes of *Poncirus trifoliata* |
| Ptrif.0002s1173.2 | Unclustered genes of *Poncirus trifoliata* |
| Ptrif.0002s1243.2 | Unclustered genes of *Poncirus trifoliata* |
| Ptrif.0002s1320.1 | Unclustered genes of *Poncirus trifoliata* |
| Ptrif.0002s1321.1 | Unclustered genes of *Poncirus trifoliata* |
| Ptrif.0002s1358.1 | Unclustered genes of *Poncirus trifoliata* |
| Ptrif.0002s1367.1 | Unclustered genes of *Poncirus trifoliata* |
| Ptrif.0002s1371.1 | Unclustered genes of *Poncirus trifoliata* |
| Ptrif.0002s1385.2 | Unclustered genes of *Poncirus trifoliata* |
| Ptrif.0002s1390.1 | Unclustered genes of *Poncirus trifoliata* |
| Ptrif.0002s1391.2 | Unclustered genes of *Poncirus trifoliata* |
| Ptrif.0002s1448.1 | Unclustered genes of *Poncirus trifoliata* |
| Ptrif.0002s1458.1 | Unclustered genes of *Poncirus trifoliata* |
| Ptrif.0002s1548.1 | Unclustered genes of *Poncirus trifoliata* |
| Ptrif.0002s1550.1 | Unclustered genes of *Poncirus trifoliata* |
| Ptrif.0002s1559.1 | Unclustered genes of *Poncirus trifoliata* |
| Ptrif.0002s1723.1 | Unclustered genes of *Poncirus trifoliata* |
| Ptrif.0002s1765.1 | Unclustered genes of *Poncirus trifoliata* |
| Ptrif.0002s1838.2 | Unclustered genes of *Poncirus trifoliata* |
| Ptrif.0002s1855.1 | Unclustered genes of *Poncirus trifoliata* |
| Ptrif.0002s1860.1 | Unclustered genes of *Poncirus trifoliata* |
| Ptrif.0002s1963.1 | Unclustered genes of *Poncirus trifoliata* |
| Ptrif.0002s2010.1 | Unclustered genes of *Poncirus trifoliata* |
| Ptrif.0002s2067.1 | Unclustered genes of *Poncirus trifoliata* |
| Ptrif.0002s2106.1 | Unclustered genes of *Poncirus trifoliata* |
| Ptrif.0002s2184.1 | Unclustered genes of *Poncirus trifoliata* |
| Ptrif.0002s3040.1 | Unclustered genes of *Poncirus trifoliata* |
| Ptrif.0002s3075.1 | Unclustered genes of *Poncirus trifoliata* |
| Ptrif.0002s3151.1 | Unclustered genes of *Poncirus trifoliata* |
| Ptrif.0002s3244.1 | Unclustered genes of *Poncirus trifoliata* |
| Ptrif.0002s3254.1 | Unclustered genes of *Poncirus trifoliata* |
| Ptrif.0002s3271.1 | Unclustered genes of *Poncirus trifoliata* |
| Ptrif.0002s3313.1 | Unclustered genes of *Poncirus trifoliata* |
| Ptrif.0002s3324.1 | Unclustered genes of *Poncirus trifoliata* |
| Ptrif.0002s3328.1 | Unclustered genes of *Poncirus trifoliata* |
| Ptrif.0002s3331.1 | Unclustered genes of *Poncirus trifoliata* |
| Ptrif.0002s3350.1 | Unclustered genes of *Poncirus trifoliata* |
| Ptrif.0002s3359.1 | Unclustered genes of *Poncirus trifoliata* |
| Ptrif.0002s3382.1 | Unclustered genes of *Poncirus trifoliata* |
| Ptrif.0002s3387.1 | Unclustered genes of *Poncirus trifoliata* |
| Ptrif.0002s3413.1 | Unclustered genes of *Poncirus trifoliata* |
| Ptrif.0002s3424.1 | Unclustered genes of *Poncirus trifoliata* |
| Ptrif.0002s3430.1 | Unclustered genes of *Poncirus trifoliata* |
| Ptrif.0002s3438.1 | Unclustered genes of *Poncirus trifoliata* |
| Ptrif.0002s3443.1 | Unclustered genes of *Poncirus trifoliata* |
| Ptrif.0002s3460.1 | Unclustered genes of *Poncirus trifoliata* |
| Ptrif.0002s3473.1 | Unclustered genes of *Poncirus trifoliata* |
| Ptrif.0002s3536.1 | Unclustered genes of *Poncirus trifoliata* |
| Ptrif.0002s3558.1 | Unclustered genes of *Poncirus trifoliata* |
| Ptrif.0003s0172.1 | Unclustered genes of *Poncirus trifoliata* |
| Ptrif.0003s0193.2 | Unclustered genes of *Poncirus trifoliata* |
| Ptrif.0003s0440.1 | Unclustered genes of *Poncirus trifoliata* |
| Ptrif.0003s0711.1 | Unclustered genes of *Poncirus trifoliata* |
| Ptrif.0003s0712.1 | Unclustered genes of *Poncirus trifoliata* |
| Ptrif.0003s0713.2 | Unclustered genes of *Poncirus trifoliata* |
| Ptrif.0003s0714.1 | Unclustered genes of *Poncirus trifoliata* |
| Ptrif.0003s0832.1 | Unclustered genes of *Poncirus trifoliata* |
| Ptrif.0003s0944.1 | Unclustered genes of *Poncirus trifoliata* |
| Ptrif.0003s0995.1 | Unclustered genes of *Poncirus trifoliata* |
| Ptrif.0003s1192.1 | Unclustered genes of *Poncirus trifoliata* |
| Ptrif.0003s1214.1 | Unclustered genes of *Poncirus trifoliata* |
| Ptrif.0003s1285.1 | Unclustered genes of *Poncirus trifoliata* |
| Ptrif.0003s1421.1 | Unclustered genes of *Poncirus trifoliata* |
| Ptrif.0003s1452.1 | Unclustered genes of *Poncirus trifoliata* |
| Ptrif.0003s1454.1 | Unclustered genes of *Poncirus trifoliata* |
| Ptrif.0003s1456.1 | Unclustered genes of *Poncirus trifoliata* |
| Ptrif.0003s1462.1 | Unclustered genes of *Poncirus trifoliata* |
| Ptrif.0003s1488.1 | Unclustered genes of *Poncirus trifoliata* |
| Ptrif.0003s1611.1 | Unclustered genes of *Poncirus trifoliata* |
| Ptrif.0003s1636.1 | Unclustered genes of *Poncirus trifoliata* |
| Ptrif.0003s1641.1 | Unclustered genes of *Poncirus trifoliata* |
| Ptrif.0003s1642.1 | Unclustered genes of *Poncirus trifoliata* |
| Ptrif.0003s1652.1 | Unclustered genes of *Poncirus trifoliata* |
| Ptrif.0003s1653.2 | Unclustered genes of *Poncirus trifoliata* |
| Ptrif.0003s1679.1 | Unclustered genes of *Poncirus trifoliata* |
| Ptrif.0003s1702.1 | Unclustered genes of *Poncirus trifoliata* |
| Ptrif.0003s1703.1 | Unclustered genes of *Poncirus trifoliata* |
| Ptrif.0003s1957.2 | Unclustered genes of *Poncirus trifoliata* |
| Ptrif.0003s1964.1 | Unclustered genes of *Poncirus trifoliata* |
| Ptrif.0003s1969.1 | Unclustered genes of *Poncirus trifoliata* |
| Ptrif.0003s1975.1 | Unclustered genes of *Poncirus trifoliata* |
| Ptrif.0003s1982.1 | Unclustered genes of *Poncirus trifoliata* |
| Ptrif.0003s1985.1 | Unclustered genes of *Poncirus trifoliata* |
| Ptrif.0003s2051.1 | Unclustered genes of *Poncirus trifoliata* |
| Ptrif.0003s2059.1 | Unclustered genes of *Poncirus trifoliata* |
| Ptrif.0003s2066.2 | Unclustered genes of *Poncirus trifoliata* |
| Ptrif.0003s2148.1 | Unclustered genes of *Poncirus trifoliata* |
| Ptrif.0003s2155.1 | Unclustered genes of *Poncirus trifoliata* |
| Ptrif.0003s2430.2 | Unclustered genes of *Poncirus trifoliata* |
| Ptrif.0003s2481.1 | Unclustered genes of *Poncirus trifoliata* |
| Ptrif.0003s2499.1 | Unclustered genes of *Poncirus trifoliata* |
| Ptrif.0003s2639.1 | Unclustered genes of *Poncirus trifoliata* |
| Ptrif.0003s2640.1 | Unclustered genes of *Poncirus trifoliata* |
| Ptrif.0003s2743.1 | Unclustered genes of *Poncirus trifoliata* |
| Ptrif.0003s2878.2 | Unclustered genes of *Poncirus trifoliata* |
| Ptrif.0003s2898.1 | Unclustered genes of *Poncirus trifoliata* |
| Ptrif.0003s2946.1 | Unclustered genes of *Poncirus trifoliata* |
| Ptrif.0003s3037.1 | Unclustered genes of *Poncirus trifoliata* |
| Ptrif.0003s3214.1 | Unclustered genes of *Poncirus trifoliata* |
| Ptrif.0003s3215.1 | Unclustered genes of *Poncirus trifoliata* |
| Ptrif.0003s3382.1 | Unclustered genes of *Poncirus trifoliata* |
| Ptrif.0003s3420.1 | Unclustered genes of *Poncirus trifoliata* |
| Ptrif.0003s3513.1 | Unclustered genes of *Poncirus trifoliata* |
| Ptrif.0003s3597.1 | Unclustered genes of *Poncirus trifoliata* |
| Ptrif.0003s3798.1 | Unclustered genes of *Poncirus trifoliata* |
| Ptrif.0003s4088.1 | Unclustered genes of *Poncirus trifoliata* |
| Ptrif.0003s4138.2 | Unclustered genes of *Poncirus trifoliata* |
| Ptrif.0003s4160.1 | Unclustered genes of *Poncirus trifoliata* |
| Ptrif.0003s4364.1 | Unclustered genes of *Poncirus trifoliata* |
| Ptrif.0003s4468.1 | Unclustered genes of *Poncirus trifoliata* |
| Ptrif.0003s4474.1 | Unclustered genes of *Poncirus trifoliata* |
| Ptrif.0003s4491.1 | Unclustered genes of *Poncirus trifoliata* |
| Ptrif.0003s4530.1 | Unclustered genes of *Poncirus trifoliata* |
| Ptrif.0003s4578.1 | Unclustered genes of *Poncirus trifoliata* |
| Ptrif.0003s4590.1 | Unclustered genes of *Poncirus trifoliata* |
| Ptrif.0003s4606.1 | Unclustered genes of *Poncirus trifoliata* |
| Ptrif.0003s4622.1 | Unclustered genes of *Poncirus trifoliata* |
| Ptrif.0003s4646.1 | Unclustered genes of *Poncirus trifoliata* |
| Ptrif.0003s4650.1 | Unclustered genes of *Poncirus trifoliata* |
| Ptrif.0003s4654.1 | Unclustered genes of *Poncirus trifoliata* |
| Ptrif.0003s4679.1 | Unclustered genes of *Poncirus trifoliata* |
| Ptrif.0003s4682.1 | Unclustered genes of *Poncirus trifoliata* |
| Ptrif.0003s4699.1 | Unclustered genes of *Poncirus trifoliata* |
| Ptrif.0003s4704.1 | Unclustered genes of *Poncirus trifoliata* |
| Ptrif.0003s4722.1 | Unclustered genes of *Poncirus trifoliata* |
| Ptrif.0003s4742.1 | Unclustered genes of *Poncirus trifoliata* |
| Ptrif.0003s4744.1 | Unclustered genes of *Poncirus trifoliata* |
| Ptrif.0003s4747.1 | Unclustered genes of *Poncirus trifoliata* |
| Ptrif.0003s4753.1 | Unclustered genes of *Poncirus trifoliata* |
| Ptrif.0003s4779.1 | Unclustered genes of *Poncirus trifoliata* |
| Ptrif.0003s4810.1 | Unclustered genes of *Poncirus trifoliata* |
| Ptrif.0003s4819.1 | Unclustered genes of *Poncirus trifoliata* |
| Ptrif.0003s4820.1 | Unclustered genes of *Poncirus trifoliata* |
| Ptrif.0003s4849.1 | Unclustered genes of *Poncirus trifoliata* |
| Ptrif.0003s4894.1 | Unclustered genes of *Poncirus trifoliata* |
| Ptrif.0003s4926.1 | Unclustered genes of *Poncirus trifoliata* |
| Ptrif.0003s4985.1 | Unclustered genes of *Poncirus trifoliata* |
| Ptrif.0004s0075.1 | Unclustered genes of *Poncirus trifoliata* |
| Ptrif.0004s0121.1 | Unclustered genes of *Poncirus trifoliata* |
| Ptrif.0004s0123.2 | Unclustered genes of *Poncirus trifoliata* |
| Ptrif.0004s0197.1 | Unclustered genes of *Poncirus trifoliata* |
| Ptrif.0004s0208.2 | Unclustered genes of *Poncirus trifoliata* |
| Ptrif.0004s0264.1 | Unclustered genes of *Poncirus trifoliata* |
| Ptrif.0004s0270.1 | Unclustered genes of *Poncirus trifoliata* |
| Ptrif.0004s0320.2 | Unclustered genes of *Poncirus trifoliata* |
| Ptrif.0004s0357.2 | Unclustered genes of *Poncirus trifoliata* |
| Ptrif.0004s0505.1 | Unclustered genes of *Poncirus trifoliata* |
| Ptrif.0004s0677.1 | Unclustered genes of *Poncirus trifoliata* |
| Ptrif.0004s0678.1 | Unclustered genes of *Poncirus trifoliata* |
| Ptrif.0004s0697.1 | Unclustered genes of *Poncirus trifoliata* |
| Ptrif.0004s0745.1 | Unclustered genes of *Poncirus trifoliata* |
| Ptrif.0004s0774.1 | Unclustered genes of *Poncirus trifoliata* |
| Ptrif.0004s0817.2 | Unclustered genes of *Poncirus trifoliata* |
| Ptrif.0004s0818.1 | Unclustered genes of *Poncirus trifoliata* |
| Ptrif.0004s0927.2 | Unclustered genes of *Poncirus trifoliata* |
| Ptrif.0004s0936.1 | Unclustered genes of *Poncirus trifoliata* |
| Ptrif.0004s0982.1 | Unclustered genes of *Poncirus trifoliata* |
| Ptrif.0004s1001.1 | Unclustered genes of *Poncirus trifoliata* |
| Ptrif.0004s1009.1 | Unclustered genes of *Poncirus trifoliata* |
| Ptrif.0004s1073.1 | Unclustered genes of *Poncirus trifoliata* |
| Ptrif.0004s1109.1 | Unclustered genes of *Poncirus trifoliata* |
| Ptrif.0004s1413.1 | Unclustered genes of *Poncirus trifoliata* |
| Ptrif.0004s1419.1 | Unclustered genes of *Poncirus trifoliata* |
| Ptrif.0004s1437.1 | Unclustered genes of *Poncirus trifoliata* |
| Ptrif.0004s1506.1 | Unclustered genes of *Poncirus trifoliata* |
| Ptrif.0004s1543.1 | Unclustered genes of *Poncirus trifoliata* |
| Ptrif.0004s1582.1 | Unclustered genes of *Poncirus trifoliata* |
| Ptrif.0004s1755.1 | Unclustered genes of *Poncirus trifoliata* |
| Ptrif.0004s1759.2 | Unclustered genes of *Poncirus trifoliata* |
| Ptrif.0004s1915.2 | Unclustered genes of *Poncirus trifoliata* |
| Ptrif.0004s2058.1 | Unclustered genes of *Poncirus trifoliata* |
| Ptrif.0004s2060.1 | Unclustered genes of *Poncirus trifoliata* |
| Ptrif.0004s2474.1 | Unclustered genes of *Poncirus trifoliata* |
| Ptrif.0004s2657.2 | Unclustered genes of *Poncirus trifoliata* |
| Ptrif.0004s2692.1 | Unclustered genes of *Poncirus trifoliata* |
| Ptrif.0004s2701.1 | Unclustered genes of *Poncirus trifoliata* |
| Ptrif.0004s2711.1 | Unclustered genes of *Poncirus trifoliata* |
| Ptrif.0004s2718.1 | Unclustered genes of *Poncirus trifoliata* |
| Ptrif.0004s2719.1 | Unclustered genes of *Poncirus trifoliata* |
| Ptrif.0004s2720.1 | Unclustered genes of *Poncirus trifoliata* |
| Ptrif.0004s2740.1 | Unclustered genes of *Poncirus trifoliata* |
| Ptrif.0004s2753.1 | Unclustered genes of *Poncirus trifoliata* |
| Ptrif.0004s2774.1 | Unclustered genes of *Poncirus trifoliata* |
| Ptrif.0004s2779.1 | Unclustered genes of *Poncirus trifoliata* |
| Ptrif.0004s2803.1 | Unclustered genes of *Poncirus trifoliata* |
| Ptrif.0004s2809.1 | Unclustered genes of *Poncirus trifoliata* |
| Ptrif.0004s2820.1 | Unclustered genes of *Poncirus trifoliata* |
| Ptrif.0004s2822.1 | Unclustered genes of *Poncirus trifoliata* |
| Ptrif.0004s2838.1 | Unclustered genes of *Poncirus trifoliata* |
| Ptrif.0004s2854.1 | Unclustered genes of *Poncirus trifoliata* |
| Ptrif.0004s2859.1 | Unclustered genes of *Poncirus trifoliata* |
| Ptrif.0004s2894.1 | Unclustered genes of *Poncirus trifoliata* |
| Ptrif.0004s2911.1 | Unclustered genes of *Poncirus trifoliata* |
| Ptrif.0004s2917.1 | Unclustered genes of *Poncirus trifoliata* |
| Ptrif.0004s2924.1 | Unclustered genes of *Poncirus trifoliata* |
| Ptrif.0004s2932.1 | Unclustered genes of *Poncirus trifoliata* |
| Ptrif.0004s3000.1 | Unclustered genes of *Poncirus trifoliata* |
| Ptrif.0004s3047.1 | Unclustered genes of *Poncirus trifoliata* |
| Ptrif.0005s0057.1 | Unclustered genes of *Poncirus trifoliata* |
| Ptrif.0005s0163.1 | Unclustered genes of *Poncirus trifoliata* |
| Ptrif.0005s0164.2 | Unclustered genes of *Poncirus trifoliata* |
| Ptrif.0005s0194.1 | Unclustered genes of *Poncirus trifoliata* |
| Ptrif.0005s0208.1 | Unclustered genes of *Poncirus trifoliata* |
| Ptrif.0005s0247.1 | Unclustered genes of *Poncirus trifoliata* |
| Ptrif.0005s0253.2 | Unclustered genes of *Poncirus trifoliata* |
| Ptrif.0005s0259.2 | Unclustered genes of *Poncirus trifoliata* |
| Ptrif.0005s0261.1 | Unclustered genes of *Poncirus trifoliata* |
| Ptrif.0005s0294.1 | Unclustered genes of *Poncirus trifoliata* |
| Ptrif.0005s0317.1 | Unclustered genes of *Poncirus trifoliata* |
| Ptrif.0005s0373.1 | Unclustered genes of *Poncirus trifoliata* |
| Ptrif.0005s0375.2 | Unclustered genes of *Poncirus trifoliata* |
| Ptrif.0005s0447.1 | Unclustered genes of *Poncirus trifoliata* |
| Ptrif.0005s0457.2 | Unclustered genes of *Poncirus trifoliata* |
| Ptrif.0005s0485.1 | Unclustered genes of *Poncirus trifoliata* |
| Ptrif.0005s0529.2 | Unclustered genes of *Poncirus trifoliata* |
| Ptrif.0005s0590.1 | Unclustered genes of *Poncirus trifoliata* |
| Ptrif.0005s0591.1 | Unclustered genes of *Poncirus trifoliata* |
| Ptrif.0005s0602.1 | Unclustered genes of *Poncirus trifoliata* |
| Ptrif.0005s0609.2 | Unclustered genes of *Poncirus trifoliata* |
| Ptrif.0005s0625.1 | Unclustered genes of *Poncirus trifoliata* |
| Ptrif.0005s0659.1 | Unclustered genes of *Poncirus trifoliata* |
| Ptrif.0005s0667.1 | Unclustered genes of *Poncirus trifoliata* |
| Ptrif.0005s0692.1 | Unclustered genes of *Poncirus trifoliata* |
| Ptrif.0005s0695.1 | Unclustered genes of *Poncirus trifoliata* |
| Ptrif.0005s0822.1 | Unclustered genes of *Poncirus trifoliata* |
| Ptrif.0005s0881.1 | Unclustered genes of *Poncirus trifoliata* |
| Ptrif.0005s0884.1 | Unclustered genes of *Poncirus trifoliata* |
| Ptrif.0005s1010.2 | Unclustered genes of *Poncirus trifoliata* |
| Ptrif.0005s1017.1 | Unclustered genes of *Poncirus trifoliata* |
| Ptrif.0005s1126.1 | Unclustered genes of *Poncirus trifoliata* |
| Ptrif.0005s1243.1 | Unclustered genes of *Poncirus trifoliata* |
| Ptrif.0005s1250.1 | Unclustered genes of *Poncirus trifoliata* |
| Ptrif.0005s1252.1 | Unclustered genes of *Poncirus trifoliata* |
| Ptrif.0005s1262.1 | Unclustered genes of *Poncirus trifoliata* |
| Ptrif.0005s1556.1 | Unclustered genes of *Poncirus trifoliata* |
| Ptrif.0005s1561.1 | Unclustered genes of *Poncirus trifoliata* |
| Ptrif.0005s1613.1 | Unclustered genes of *Poncirus trifoliata* |
| Ptrif.0005s1641.1 | Unclustered genes of *Poncirus trifoliata* |
| Ptrif.0005s1690.1 | Unclustered genes of *Poncirus trifoliata* |
| Ptrif.0005s1896.1 | Unclustered genes of *Poncirus trifoliata* |
| Ptrif.0005s2004.1 | Unclustered genes of *Poncirus trifoliata* |
| Ptrif.0005s2248.1 | Unclustered genes of *Poncirus trifoliata* |
| Ptrif.0005s2249.1 | Unclustered genes of *Poncirus trifoliata* |
| Ptrif.0005s2415.1 | Unclustered genes of *Poncirus trifoliata* |
| Ptrif.0005s2692.1 | Unclustered genes of *Poncirus trifoliata* |
| Ptrif.0005s2806.1 | Unclustered genes of *Poncirus trifoliata* |
| Ptrif.0005s2828.1 | Unclustered genes of *Poncirus trifoliata* |
| Ptrif.0005s2839.2 | Unclustered genes of *Poncirus trifoliata* |
| Ptrif.0005s2841.1 | Unclustered genes of *Poncirus trifoliata* |
| Ptrif.0005s2860.1 | Unclustered genes of *Poncirus trifoliata* |
| Ptrif.0005s3030.1 | Unclustered genes of *Poncirus trifoliata* |
| Ptrif.0005s3033.1 | Unclustered genes of *Poncirus trifoliata* |
| Ptrif.0005s3038.1 | Unclustered genes of *Poncirus trifoliata* |
| Ptrif.0005s3052.1 | Unclustered genes of *Poncirus trifoliata* |
| Ptrif.0005s3056.1 | Unclustered genes of *Poncirus trifoliata* |
| Ptrif.0005s3061.1 | Unclustered genes of *Poncirus trifoliata* |
| Ptrif.0005s3066.1 | Unclustered genes of *Poncirus trifoliata* |
| Ptrif.0005s3091.1 | Unclustered genes of *Poncirus trifoliata* |
| Ptrif.0005s3097.1 | Unclustered genes of *Poncirus trifoliata* |
| Ptrif.0005s3123.1 | Unclustered genes of *Poncirus trifoliata* |
| Ptrif.0005s3142.1 | Unclustered genes of *Poncirus trifoliata* |
| Ptrif.0005s3145.1 | Unclustered genes of *Poncirus trifoliata* |
| Ptrif.0005s3180.1 | Unclustered genes of *Poncirus trifoliata* |
| Ptrif.0005s3217.1 | Unclustered genes of *Poncirus trifoliata* |
| Ptrif.0005s3315.1 | Unclustered genes of *Poncirus trifoliata* |
| Ptrif.0005s3332.1 | Unclustered genes of *Poncirus trifoliata* |
| Ptrif.0005s3378.1 | Unclustered genes of *Poncirus trifoliata* |
| Ptrif.0005s3425.1 | Unclustered genes of *Poncirus trifoliata* |
| Ptrif.0006s0041.1 | Unclustered genes of *Poncirus trifoliata* |
| Ptrif.0006s0043.1 | Unclustered genes of *Poncirus trifoliata* |
| Ptrif.0006s0050.1 | Unclustered genes of *Poncirus trifoliata* |
| Ptrif.0006s0052.1 | Unclustered genes of *Poncirus trifoliata* |
| Ptrif.0006s0107.1 | Unclustered genes of *Poncirus trifoliata* |
| Ptrif.0006s0235.3 | Unclustered genes of *Poncirus trifoliata* |
| Ptrif.0006s0236.1 | Unclustered genes of *Poncirus trifoliata* |
| Ptrif.0006s0600.2 | Unclustered genes of *Poncirus trifoliata* |
| Ptrif.0006s0647.1 | Unclustered genes of *Poncirus trifoliata* |
| Ptrif.0006s0723.1 | Unclustered genes of *Poncirus trifoliata* |
| Ptrif.0006s0847.1 | Unclustered genes of *Poncirus trifoliata* |
| Ptrif.0006s1079.2 | Unclustered genes of *Poncirus trifoliata* |
| Ptrif.0006s1099.1 | Unclustered genes of *Poncirus trifoliata* |
| Ptrif.0006s1305.2 | Unclustered genes of *Poncirus trifoliata* |
| Ptrif.0006s1497.1 | Unclustered genes of *Poncirus trifoliata* |
| Ptrif.0006s1620.1 | Unclustered genes of *Poncirus trifoliata* |
| Ptrif.0006s1766.2 | Unclustered genes of *Poncirus trifoliata* |
| Ptrif.0006s2067.2 | Unclustered genes of *Poncirus trifoliata* |
| Ptrif.0006s2211.1 | Unclustered genes of *Poncirus trifoliata* |
| Ptrif.0006s2281.1 | Unclustered genes of *Poncirus trifoliata* |
| Ptrif.0006s2305.1 | Unclustered genes of *Poncirus trifoliata* |
| Ptrif.0006s2306.1 | Unclustered genes of *Poncirus trifoliata* |
| Ptrif.0006s2311.1 | Unclustered genes of *Poncirus trifoliata* |
| Ptrif.0006s2316.1 | Unclustered genes of *Poncirus trifoliata* |
| Ptrif.0006s2318.1 | Unclustered genes of *Poncirus trifoliata* |
| Ptrif.0006s2352.1 | Unclustered genes of *Poncirus trifoliata* |
| Ptrif.0006s2404.1 | Unclustered genes of *Poncirus trifoliata* |
| Ptrif.0006s2412.1 | Unclustered genes of *Poncirus trifoliata* |
| Ptrif.0006s2464.1 | Unclustered genes of *Poncirus trifoliata* |
| Ptrif.0006s2473.1 | Unclustered genes of *Poncirus trifoliata* |
| Ptrif.0006s2474.1 | Unclustered genes of *Poncirus trifoliata* |
| Ptrif.0006s2488.1 | Unclustered genes of *Poncirus trifoliata* |
| Ptrif.0006s2498.1 | Unclustered genes of *Poncirus trifoliata* |
| Ptrif.0006s2506.1 | Unclustered genes of *Poncirus trifoliata* |
| Ptrif.0007s0478.1 | Unclustered genes of *Poncirus trifoliata* |
| Ptrif.0007s0513.1 | Unclustered genes of *Poncirus trifoliata* |
| Ptrif.0007s0562.1 | Unclustered genes of *Poncirus trifoliata* |
| Ptrif.0007s0647.1 | Unclustered genes of *Poncirus trifoliata* |
| Ptrif.0007s0699.1 | Unclustered genes of *Poncirus trifoliata* |
| Ptrif.0007s0703.2 | Unclustered genes of *Poncirus trifoliata* |
| Ptrif.0007s0709.1 | Unclustered genes of *Poncirus trifoliata* |
| Ptrif.0007s0720.1 | Unclustered genes of *Poncirus trifoliata* |
| Ptrif.0007s0889.1 | Unclustered genes of *Poncirus trifoliata* |
| Ptrif.0007s1110.1 | Unclustered genes of *Poncirus trifoliata* |
| Ptrif.0007s1208.1 | Unclustered genes of *Poncirus trifoliata* |
| Ptrif.0007s1266.1 | Unclustered genes of *Poncirus trifoliata* |
| Ptrif.0007s1276.1 | Unclustered genes of *Poncirus trifoliata* |
| Ptrif.0007s1291.1 | Unclustered genes of *Poncirus trifoliata* |
| Ptrif.0007s1314.1 | Unclustered genes of *Poncirus trifoliata* |
| Ptrif.0007s1472.1 | Unclustered genes of *Poncirus trifoliata* |
| Ptrif.0007s1539.1 | Unclustered genes of *Poncirus trifoliata* |
| Ptrif.0007s1618.1 | Unclustered genes of *Poncirus trifoliata* |
| Ptrif.0007s1741.1 | Unclustered genes of *Poncirus trifoliata* |
| Ptrif.0007s1799.1 | Unclustered genes of *Poncirus trifoliata* |
| Ptrif.0007s1807.2 | Unclustered genes of *Poncirus trifoliata* |
| Ptrif.0007s1839.1 | Unclustered genes of *Poncirus trifoliata* |
| Ptrif.0007s1927.1 | Unclustered genes of *Poncirus trifoliata* |
| Ptrif.0007s2074.1 | Unclustered genes of *Poncirus trifoliata* |
| Ptrif.0007s2186.1 | Unclustered genes of *Poncirus trifoliata* |
| Ptrif.0007s2224.1 | Unclustered genes of *Poncirus trifoliata* |
| Ptrif.0007s2349.1 | Unclustered genes of *Poncirus trifoliata* |
| Ptrif.0007s2456.1 | Unclustered genes of *Poncirus trifoliata* |
| Ptrif.0007s2471.1 | Unclustered genes of *Poncirus trifoliata* |
| Ptrif.0007s2572.1 | Unclustered genes of *Poncirus trifoliata* |
| Ptrif.0007s2573.1 | Unclustered genes of *Poncirus trifoliata* |
| Ptrif.0007s2579.1 | Unclustered genes of *Poncirus trifoliata* |
| Ptrif.0007s2600.1 | Unclustered genes of *Poncirus trifoliata* |
| Ptrif.0007s2631.1 | Unclustered genes of *Poncirus trifoliata* |
| Ptrif.0007s2643.1 | Unclustered genes of *Poncirus trifoliata* |
| Ptrif.0007s2648.1 | Unclustered genes of *Poncirus trifoliata* |
| Ptrif.0007s2700.1 | Unclustered genes of *Poncirus trifoliata* |
| Ptrif.0007s2711.1 | Unclustered genes of *Poncirus trifoliata* |
| Ptrif.0007s2733.1 | Unclustered genes of *Poncirus trifoliata* |
| Ptrif.0007s2734.1 | Unclustered genes of *Poncirus trifoliata* |
| Ptrif.0007s2789.1 | Unclustered genes of *Poncirus trifoliata* |
| Ptrif.0007s2811.1 | Unclustered genes of *Poncirus trifoliata* |
| Ptrif.0007s2843.1 | Unclustered genes of *Poncirus trifoliata* |
| Ptrif.0007s2846.1 | Unclustered genes of *Poncirus trifoliata* |
| Ptrif.0007s2866.1 | Unclustered genes of *Poncirus trifoliata* |
| Ptrif.0007s2877.1 | Unclustered genes of *Poncirus trifoliata* |
| Ptrif.0007s2947.1 | Unclustered genes of *Poncirus trifoliata* |
| Ptrif.0008s0084.1 | Unclustered genes of *Poncirus trifoliata* |
| Ptrif.0008s0160.1 | Unclustered genes of *Poncirus trifoliata* |
| Ptrif.0008s0223.1 | Unclustered genes of *Poncirus trifoliata* |
| Ptrif.0008s0308.2 | Unclustered genes of *Poncirus trifoliata* |
| Ptrif.0008s0325.1 | Unclustered genes of *Poncirus trifoliata* |
| Ptrif.0008s0377.1 | Unclustered genes of *Poncirus trifoliata* |
| Ptrif.0008s0516.1 | Unclustered genes of *Poncirus trifoliata* |
| Ptrif.0008s0629.1 | Unclustered genes of *Poncirus trifoliata* |
| Ptrif.0008s0633.1 | Unclustered genes of *Poncirus trifoliata* |
| Ptrif.0008s0659.2 | Unclustered genes of *Poncirus trifoliata* |
| Ptrif.0008s0663.1 | Unclustered genes of *Poncirus trifoliata* |
| Ptrif.0008s0886.1 | Unclustered genes of *Poncirus trifoliata* |
| Ptrif.0008s0889.1 | Unclustered genes of *Poncirus trifoliata* |
| Ptrif.0008s0895.1 | Unclustered genes of *Poncirus trifoliata* |
| Ptrif.0008s0997.1 | Unclustered genes of *Poncirus trifoliata* |
| Ptrif.0008s1046.1 | Unclustered genes of *Poncirus trifoliata* |
| Ptrif.0008s1047.1 | Unclustered genes of *Poncirus trifoliata* |
| Ptrif.0008s1056.1 | Unclustered genes of *Poncirus trifoliata* |
| Ptrif.0008s1086.1 | Unclustered genes of *Poncirus trifoliata* |
| Ptrif.0008s1088.2 | Unclustered genes of *Poncirus trifoliata* |
| Ptrif.0008s1091.1 | Unclustered genes of *Poncirus trifoliata* |
| Ptrif.0008s1094.1 | Unclustered genes of *Poncirus trifoliata* |
| Ptrif.0008s1097.1 | Unclustered genes of *Poncirus trifoliata* |
| Ptrif.0008s1099.1 | Unclustered genes of *Poncirus trifoliata* |
| Ptrif.0008s1100.1 | Unclustered genes of *Poncirus trifoliata* |
| Ptrif.0008s1101.1 | Unclustered genes of *Poncirus trifoliata* |
| Ptrif.0008s1103.1 | Unclustered genes of *Poncirus trifoliata* |
| Ptrif.0008s1105.1 | Unclustered genes of *Poncirus trifoliata* |
| Ptrif.0008s1106.1 | Unclustered genes of *Poncirus trifoliata* |
| Ptrif.0008s1126.1 | Unclustered genes of *Poncirus trifoliata* |
| Ptrif.0008s1229.1 | Unclustered genes of *Poncirus trifoliata* |
| Ptrif.0008s1317.1 | Unclustered genes of *Poncirus trifoliata* |
| Ptrif.0008s1326.1 | Unclustered genes of *Poncirus trifoliata* |
| Ptrif.0008s1330.1 | Unclustered genes of *Poncirus trifoliata* |
| Ptrif.0008s1355.1 | Unclustered genes of *Poncirus trifoliata* |
| Ptrif.0008s1360.1 | Unclustered genes of *Poncirus trifoliata* |
| Ptrif.0008s1514.1 | Unclustered genes of *Poncirus trifoliata* |
| Ptrif.0008s1553.1 | Unclustered genes of *Poncirus trifoliata* |
| Ptrif.0008s1587.1 | Unclustered genes of *Poncirus trifoliata* |
| Ptrif.0008s1620.2 | Unclustered genes of *Poncirus trifoliata* |
| Ptrif.0008s1672.2 | Unclustered genes of *Poncirus trifoliata* |
| Ptrif.0008s1700.1 | Unclustered genes of *Poncirus trifoliata* |
| Ptrif.0008s1713.3 | Unclustered genes of *Poncirus trifoliata* |
| Ptrif.0008s1745.1 | Unclustered genes of *Poncirus trifoliata* |
| Ptrif.0008s1807.1 | Unclustered genes of *Poncirus trifoliata* |
| Ptrif.0008s1898.1 | Unclustered genes of *Poncirus trifoliata* |
| Ptrif.0008s1942.1 | Unclustered genes of *Poncirus trifoliata* |
| Ptrif.0008s2224.1 | Unclustered genes of *Poncirus trifoliata* |
| Ptrif.0008s2268.1 | Unclustered genes of *Poncirus trifoliata* |
| Ptrif.0008s2327.1 | Unclustered genes of *Poncirus trifoliata* |
| Ptrif.0008s2476.1 | Unclustered genes of *Poncirus trifoliata* |
| Ptrif.0008s2504.1 | Unclustered genes of *Poncirus trifoliata* |
| Ptrif.0008s2532.1 | Unclustered genes of *Poncirus trifoliata* |
| Ptrif.0008s2533.1 | Unclustered genes of *Poncirus trifoliata* |
| Ptrif.0008s2547.1 | Unclustered genes of *Poncirus trifoliata* |
| Ptrif.0008s2583.1 | Unclustered genes of *Poncirus trifoliata* |
| Ptrif.0008s2584.1 | Unclustered genes of *Poncirus trifoliata* |
| Ptrif.0008s2586.1 | Unclustered genes of *Poncirus trifoliata* |
| Ptrif.0008s2599.1 | Unclustered genes of *Poncirus trifoliata* |
| Ptrif.0008s2602.1 | Unclustered genes of *Poncirus trifoliata* |
| Ptrif.0008s2603.1 | Unclustered genes of *Poncirus trifoliata* |
| Ptrif.0008s2606.1 | Unclustered genes of *Poncirus trifoliata* |
| Ptrif.0008s2629.1 | Unclustered genes of *Poncirus trifoliata* |
| Ptrif.0008s2664.1 | Unclustered genes of *Poncirus trifoliata* |
| Ptrif.0008s2683.1 | Unclustered genes of *Poncirus trifoliata* |
| Ptrif.0008s2695.1 | Unclustered genes of *Poncirus trifoliata* |
| Ptrif.0008s2697.1 | Unclustered genes of *Poncirus trifoliata* |
| Ptrif.0008s2710.1 | Unclustered genes of *Poncirus trifoliata* |
| Ptrif.0008s2712.1 | Unclustered genes of *Poncirus trifoliata* |
| Ptrif.0008s2718.1 | Unclustered genes of *Poncirus trifoliata* |
| Ptrif.0008s2758.1 | Unclustered genes of *Poncirus trifoliata* |
| Ptrif.0008s2762.1 | Unclustered genes of *Poncirus trifoliata* |
| Ptrif.0008s2764.1 | Unclustered genes of *Poncirus trifoliata* |
| Ptrif.0009s0119.1 | Unclustered genes of *Poncirus trifoliata* |
| Ptrif.0009s0123.1 | Unclustered genes of *Poncirus trifoliata* |
| Ptrif.0009s0124.4 | Unclustered genes of *Poncirus trifoliata* |
| Ptrif.0009s0188.1 | Unclustered genes of *Poncirus trifoliata* |
| Ptrif.0009s0387.1 | Unclustered genes of *Poncirus trifoliata* |
| Ptrif.0009s0412.2 | Unclustered genes of *Poncirus trifoliata* |
| Ptrif.0009s0599.1 | Unclustered genes of *Poncirus trifoliata* |
| Ptrif.0009s0628.1 | Unclustered genes of *Poncirus trifoliata* |
| Ptrif.0009s0700.1 | Unclustered genes of *Poncirus trifoliata* |
| Ptrif.0009s0711.1 | Unclustered genes of *Poncirus trifoliata* |
| Ptrif.0009s0780.1 | Unclustered genes of *Poncirus trifoliata* |
| Ptrif.0009s0798.1 | Unclustered genes of *Poncirus trifoliata* |
| Ptrif.0009s0839.1 | Unclustered genes of *Poncirus trifoliata* |
| Ptrif.0009s0855.1 | Unclustered genes of *Poncirus trifoliata* |
| Ptrif.0009s0912.1 | Unclustered genes of *Poncirus trifoliata* |
| Ptrif.0009s0976.1 | Unclustered genes of *Poncirus trifoliata* |
| Ptrif.0009s1044.1 | Unclustered genes of *Poncirus trifoliata* |
| Ptrif.0009s1051.1 | Unclustered genes of *Poncirus trifoliata* |
| Ptrif.0009s1094.1 | Unclustered genes of *Poncirus trifoliata* |
| Ptrif.0009s1151.1 | Unclustered genes of *Poncirus trifoliata* |
| Ptrif.0009s1228.1 | Unclustered genes of *Poncirus trifoliata* |
| Ptrif.0009s1299.1 | Unclustered genes of *Poncirus trifoliata* |
| Ptrif.0009s1334.1 | Unclustered genes of *Poncirus trifoliata* |
| Ptrif.0009s1354.1 | Unclustered genes of *Poncirus trifoliata* |
| Ptrif.0009s1355.1 | Unclustered genes of *Poncirus trifoliata* |
| Ptrif.0009s1392.1 | Unclustered genes of *Poncirus trifoliata* |
| Ptrif.0009s1393.1 | Unclustered genes of *Poncirus trifoliata* |
| Ptrif.0009s1492.1 | Unclustered genes of *Poncirus trifoliata* |
| Ptrif.0009s1516.1 | Unclustered genes of *Poncirus trifoliata* |
| Ptrif.0009s1530.1 | Unclustered genes of *Poncirus trifoliata* |
| Ptrif.0009s1562.2 | Unclustered genes of *Poncirus trifoliata* |
| Ptrif.0009s1567.1 | Unclustered genes of *Poncirus trifoliata* |
| Ptrif.0009s1573.1 | Unclustered genes of *Poncirus trifoliata* |
| Ptrif.0009s1574.1 | Unclustered genes of *Poncirus trifoliata* |
| Ptrif.0009s1579.1 | Unclustered genes of *Poncirus trifoliata* |
| Ptrif.0009s1581.1 | Unclustered genes of *Poncirus trifoliata* |
| Ptrif.0009s1625.1 | Unclustered genes of *Poncirus trifoliata* |
| Ptrif.0009s1691.1 | Unclustered genes of *Poncirus trifoliata* |
| Ptrif.0009s1725.1 | Unclustered genes of *Poncirus trifoliata* |
| Ptrif.0009s1754.1 | Unclustered genes of *Poncirus trifoliata* |
| Ptrif.0009s1995.2 | Unclustered genes of *Poncirus trifoliata* |
| Ptrif.0009s2070.3 | Unclustered genes of *Poncirus trifoliata* |
| Ptrif.0009s2256.1 | Unclustered genes of *Poncirus trifoliata* |
| Ptrif.0009s2396.1 | Unclustered genes of *Poncirus trifoliata* |
| Ptrif.0009s2435.1 | Unclustered genes of *Poncirus trifoliata* |
| Ptrif.0009s2446.1 | Unclustered genes of *Poncirus trifoliata* |
| Ptrif.0009s2462.1 | Unclustered genes of *Poncirus trifoliata* |
| Ptrif.0009s2468.1 | Unclustered genes of *Poncirus trifoliata* |
| Ptrif.0009s2515.1 | Unclustered genes of *Poncirus trifoliata* |
| Ptrif.0009s2525.1 | Unclustered genes of *Poncirus trifoliata* |
| Ptrif.0009s2528.1 | Unclustered genes of *Poncirus trifoliata* |
| Ptrif.0009s2530.1 | Unclustered genes of *Poncirus trifoliata* |
| Ptrif.0009s2540.1 | Unclustered genes of *Poncirus trifoliata* |
| Ptrif.0009s2541.1 | Unclustered genes of *Poncirus trifoliata* |
| Ptrif.0009s2590.1 | Unclustered genes of *Poncirus trifoliata* |
| Ptrif.0009s2599.1 | Unclustered genes of *Poncirus trifoliata* |
| Ptrif.0009s2616.1 | Unclustered genes of *Poncirus trifoliata* |
| Ptrif.0009s2630.1 | Unclustered genes of *Poncirus trifoliata* |
| Ptrif.0009s2632.1 | Unclustered genes of *Poncirus trifoliata* |
| Ptrif.0009s2640.1 | Unclustered genes of *Poncirus trifoliata* |
| Ptrif.0009s2642.1 | Unclustered genes of *Poncirus trifoliata* |
| Ptrif.0009s2645.1 | Unclustered genes of *Poncirus trifoliata* |
| Ptrif.0009s2665.1 | Unclustered genes of *Poncirus trifoliata* |
| Ptrif.0009s2713.1 | Unclustered genes of *Poncirus trifoliata* |
| Ptrif.0009s2714.1 | Unclustered genes of *Poncirus trifoliata* |
| Ptrif.0016s0001.1 | Unclustered genes of *Poncirus trifoliata* |
| Ptrif.0016s0002.1 | Unclustered genes of *Poncirus trifoliata* |
| Ptrif.0023s0002.1 | Unclustered genes of *Poncirus trifoliata* |
| Ptrif.0040s0001.1 | Unclustered genes of *Poncirus trifoliata* |
| Ptrif.0040s0003.1 | Unclustered genes of *Poncirus trifoliata* |
| Ptrif.0001s0457.1 | Belong to *Poncirus*-specific gene families |
| Ptrif.0001s1147.1 | Belong to *Poncirus*-specific gene families |
| Ptrif.0002s0223.1 | Belong to *Poncirus*-specific gene families |
| Ptrif.0002s0436.1 | Belong to *Poncirus*-specific gene families |
| Ptrif.0002s3124.1 | Belong to *Poncirus*-specific gene families |
| Ptrif.0003s1191.1 | Belong to *Poncirus*-specific gene families |
| Ptrif.0003s1822.1 | Belong to *Poncirus*-specific gene families |
| Ptrif.0003s3072.1 | Belong to *Poncirus*-specific gene families |
| Ptrif.0003s3073.1 | Belong to *Poncirus*-specific gene families |
| Ptrif.0003s4429.1 | Belong to *Poncirus*-specific gene families |
| Ptrif.0003s4746.1 | Belong to *Poncirus*-specific gene families |
| Ptrif.0003s4893.1 | Belong to *Poncirus*-specific gene families |
| Ptrif.0004s1465.1 | Belong to *Poncirus*-specific gene families |
| Ptrif.0004s2891.1 | Belong to *Poncirus*-specific gene families |
| Ptrif.0004s3036.1 | Belong to *Poncirus*-specific gene families |
| Ptrif.0005s0173.1 | Belong to *Poncirus*-specific gene families |
| Ptrif.0005s0180.1 | Belong to *Poncirus*-specific gene families |
| Ptrif.0005s3105.1 | Belong to *Poncirus*-specific gene families |
| Ptrif.0007s0777.1 | Belong to *Poncirus*-specific gene families |
| Ptrif.0007s2538.1 | Belong to *Poncirus*-specific gene families |
| Ptrif.0007s2577.1 | Belong to *Poncirus*-specific gene families |
| Ptrif.0007s2581.1 | Belong to *Poncirus*-specific gene families |
| Ptrif.0007s2754.1 | Belong to *Poncirus*-specific gene families |
| Ptrif.0008s1250.2 | Belong to *Poncirus*-specific gene families |
| Ptrif.0008s1341.1 | Belong to *Poncirus*-specific gene families |
| Ptrif.0008s1345.1 | Belong to *Poncirus*-specific gene families |
| Ptrif.0008s1650.1 | Belong to *Poncirus*-specific gene families |
| Ptrif.0008s2572.1 | Belong to *Poncirus*-specific gene families |
| Ptrif.0008s2628.1 | Belong to *Poncirus*-specific gene families |
| Ptrif.0009s1830.1 | Belong to *Poncirus*-specific gene families |
| Ptrif.0009s1831.1 | Belong to *Poncirus*-specific gene families |
| Ptrif.0141s0101.1 | Belong to *Poncirus*-specific gene families |
| Ptrif.0142s0100.1 | Belong to *Poncirus*-specific gene families |
| Ptrif.0144s0001.2 | Belong to *Poncirus*-specific gene families |
| Ptrif.0145s0001.1 | Belong to *Poncirus*-specific gene families |
| Ptrif.0146s0002.1 | Belong to *Poncirus*-specific gene families |
| Ptrif.0146s0102.1 | Belong to *Poncirus*-specific gene families |
| Ptrif.0148s0001.1 | Belong to *Poncirus*-specific gene families |
| Ptrif.0148s0002.1 | Belong to *Poncirus*-specific gene families |
| Ptrif.0150s0001.1 | Belong to *Poncirus*-specific gene families |
| Ptrif.0150s0101.1 | Belong to *Poncirus*-specific gene families |
| Ptrif.0151s0001.1 | Belong to *Poncirus*-specific gene families |

**Table 10.** Summary of re-annotation of single-copy gene families across 10 genomes.

| Genome | Re-annotated single-copy gene models (5751 seeding peptides) | | |
| --- | --- | --- | --- |
|  | No. of recovered genes | Average CDS similarity with pre-existing models | CDS 100% same with pre-existing models |
| *A. buxifolia* | 5557 (96.63%) | 96.55% | 4122 (71.67%) |
| *C. clementina* | 5616 (97.65%) | 97.24% | 4513 (78.47%) |
| *C. ichangensis* | 5634 (97.97%) | 96.60% | 4367 (75.93%) |
| *C. maxima* | 5636 (98.00%) | 96.96% | 4491 (78.09%) |
| *C. medica* | 5606 (97.48%) | 96.57% | 4304 (74.84%) |
| *C. reticulata* | 5625 (97.81%) | 96.44% | 4426 (76.96%) |
| *C. unshiu* | 5542 (96.37%) | 95.47% | 3934 (68.41%) |
| *F. hindsii* | 5606 (97.48%) | 96.31% | 4330 (75.29%) |
| *C. sinensis* | 5627 (97.84%) | 97.09% | 4539 (78.93%) |
| *P. trifoliata* | 5741 (99.83%) | 98.98% | 5533 (96.21%) |

**Table S11.** Summary of rapid evolving gene families in *P. trifoliata* lineage from CAFÉ analysis.

Table S11 is submitted separately due to a large size.

**Table S12.** Gene ontology enrichment analysis of *P. trifoliata*-specific genes and fast evolving genes in *P. trifoliata*.

Table S12 is submitted separately due to a large size.

**Table S13.** Summary of positively selected genes.

Table S13 is submitted separately due to a large size.

**Table S14.** QTLs associated with Huanglongbing tolerance mapped to *Poncirus trifoliata* genome and locations of *Ctv* and *Tyr1* regions.

| QTL name | Trait | Time | Phenotypic variation explained (%) | Location on *Citrus x clementina* genome | | | Location on *Poncirus trifoliata* genome | | | Physical size (Mb) |
| --- | --- | --- | --- | --- | --- | --- | --- | --- | --- | --- |
|  |  |  |  | scaffold | start | end | scaffold | start | end |  |
| CD-2015-t6 | Canopy damage | 2015 | 22 | scaffold_6 | 16395904 | 18659973 | scaffold_6 | 13796799 | 15980951 | 2.18 |
| CD-2015-t8 | Canopy damage | 2015 | 16.8 | scaffold_8 | 9646437 | 17264644 | scaffold_8 | 8755447 | 19363458 | 10.61 |
| CD-2015-t9 | Canopy damage | 2015 | 21.2 | scaffold_9 | 17990974 | 22736095 | scaffold_9 | 13047222 | 16103880 | 3.06 |
| CD-2016-t6 | Canopy damage | 2016 | 14.6 | scaffold_6 | 15161846 | 15804706 | scaffold_6 | 12763250 | 13327851 | 0.56 |
| CD-2016-t7 | Canopy damage | 2016 | 15.9 | scaffold_7 | 4878808 | 6389778 | scaffold_7 | 4367353 | 5824743 | 1.46 |
| FS-2015-t6a | Foliar symptom | 2015 | 20.9 | scaffold_6 | 16395904 | 17858316 | scaffold_6 | 13796799 | 15148321 | 1.35 |
| FS-2015-t6b | Foliar symptom | 2015 | 20.8 | scaffold_6 | 18489848 | 20566262 | scaffold_6 | 15769382 | 17888475 | 2.12 |
| FS-2015-t8a | Foliar symptom | 2015 | 22.1 | scaffold_8 | 5494424 | 6930561 | scaffold_8 | 5228815 | 6567490 | 1.34 |
| FS-2015-t8b | Foliar symptom | 2015 | 24.6 | scaffold_8 | 11058273 | 17540866 | scaffold_8 | 9928054 | 19602409 | 9.67 |
| FS-2015-t9a | Foliar symptom | 2015 | 24.5 | scaffold_9 | 21990015 | 25309054 | scaffold_9 | 16683266 | 20431536 | 3.75 |
| FS-2015-t9b | Foliar symptom | 2015 | 20.8 | scaffold_9 | 27148980 | 27791447 | scaffold_9 | 22451088 | 22939583 | 0.49 |
| FS-2016-t6 | Foliar symptom | 2016 | 29.9 | scaffold_6 | 15161846 | 20566262 | scaffold_6 | 12763250 | 17888475 | 5.13 |
| FS-2016-t8 | Foliar symptom | 2016 | 13.9 | scaffold_8 | 17264644 | 17540866 | scaffold_8 | 19363181 | 19602409 | 0.24 |
| FS-2016-t9 | Foliar symptom | 2016 | 18.1 | scaffold_9 | 27515104 | 27791447 | scaffold_9 | 22690250 | 22939583 | 0.25 |
| Ctv region | Citrus tristeza virus disease |  |  |  |  |  | scaffold_7 | 11739669 | 11979231 | 0.24 |
| Tyr1 region | Nematode resistance |  |  |  |  |  | scaffold_7 | 9842401 | 10883670 | 1.04 |
| Marker Pt8 for Tyr1 | Nematode resistance |  |  |  |  |  | scaffold_7 | 9842401 | 9842822 |  |
| Marker SCO07 for Tyr1 | Nematode resistance |  |  |  |  |  | scaffold_7 | 10883021 | 10883670 |  |

**Table S15.** Candidate *Poncirus trifoliata* genes responsive to *C*Las infection and located within QTLs associated with Huanglongbing tolerance.

| Differentially expressed genes | CLas infected species | Expression Log2 fold change | Counterpart* in *Poncirus trifoliata* | Located within QTL | Positive selection** | Gene function | Transcriptome study |
| --- | --- | --- | --- | --- | --- | --- | --- |
| orange1.1g008366m | *Citrus x sinensis* | 2.2 | Ptrif.0006s0773.1 | CD-2016-t6, FS-2016-t6 |  | laccase7 | Fu et al., 2016 |
| orange1.1g020291m | *Citrus x sinensis* | 4.9 | Ptrif.0006s1042.1 | CD-2015-t6, FS-2015-t6a, FS-2016-t6 | Yes | transcription factor WRKY70 | Fu et al., 2016 |
| orange1.1g044722m | *Citrus x sinensis* | 1.4 | Ptrif.0006s1224.1 | FS-2015-t6b, FS-2016-t6 |  | myb-like HTH transcriptional regulator family protein | Fu et al., 2016 |
| orange1.1g029641m | *Citrus x sinensis* | 2.2 | Ptrif.0006s1285.1 | FS-2015-t6b, FS-2016-t6 |  | Disease resistance-responsive (dirigent-like protein) family protein | Fu et al., 2016 |
| Ciclev10013749m | *Citrus x sinensis* | 4.94 | Ptrif.0006s1339.1 | FS-2015-t6b, FS-2016-t6 |  | VQ motif-containing protein | Yu et al., 2017 |
| Ciclev10011844m | *Citrus sinensis* and *Citrus jambhiri* | 5.42 | Ptrif.0006s1374.1 | FS-2015-t6b, FS-2016-t6 |  | plant U-box 22 | Yu et al., 2017 |
| orange1.1g047927m | *Citrus x sinensis* | 1.2 | Ptrif.0006s2381.1 | CD-2015-t6, FS-2015-t6a, FS-2016-t6 |  | receptor like protein 6 | Fu et al., 2016 |
| Cs1g20230.2 (102630988) | *Citrus x sinensis* | -1.6 | Ptrif.0007s0712.1 | CD-2016-t7 |  | peroxidase 4-like | Hu et al., 2017 |
| orange1.1g041382m | *Citrus x sinensis* | 1.6 | Ptrif.0007s0808.1 | CD-2016-t7 |  | cytochrome BC1 synthesis | Fu et al., 2016 |
| Ciclev10026096m | *Citrus x sinensis* | 5.3 | Ptrif.0007s0854.1 | CD-2016-t7 |  | Pyridoxal phosphate phosphatase-related protein | Yu et al., 2017 |
| orange1.1g011254m | *Citrus x sinensis* | 1.0 | Ptrif.0008s0764.2 | FS-2015-t8a |  | cytochrome BC1 synthesis | Fu et al., 2016 |
| orange1.1t01983.1 (102621540) | *Citrus x sinensis* | -1.3 | Ptrif.0008s1137.2 | CD-2015-t8, FS-2015-t8b |  | UDP-glycosyltransferase 73C3-like | Hu et al., 2017 |
| orange1.1t01984.1 (102621842) | *Citrus hystrix* | 1 | Ptrif.0008s1139.2 | CD-2015-t8, FS-2015-t8b |  | UDP-glycosyltransferase 73C6-like | Hu et al., 2017 |
| orange1.1g032389m | *Citrus x sinensis* | 1.0 | Ptrif.0008s1497.1 | FS-2015-t8b, FS-2016-t8 |  | pathogenesis-related 4 | Fu et al., 2016 |
| orange1.1t00706.1 (NBS) (102624560) | *Citrus x sinensis* | 1 | Ptrif.0009s1449.1 | FS-2015-t9a |  | TIR-NBS | Hu et al., 2017 |
| orange1.1t02930.1 (102619375) | *Citrus x sinensis* | 1.2 | Ptrif.0009s1511.1 | FS-2015-t9a |  | LRR receptor-like serine/threonine-protein kinase GSO1 | Hu et al., 2017 |
| orange1.1g044801m | *Citrus x sinensis* | 1.7 | Ptrif.0009s1874.1 | FS-2015-t9b |  | chitinaseA | Fu et al., 2016 |
| orange1.1g019027m | *Citrus x sinensis* | 7.0 | Ptrif.0009s1899.1 | FS-2015-t9b |  | 2-oxoglutarate (2OG) and Fe(II)-dependent oxygenases | Fu et al., 2016 |
| orange1.1g027094m | *Citrus x sinensis* | 1.5 | Ptrif.0009s1909.1 | FS-2015-t9b, FS-2016-t9 |  | Plant invertase/pectinmethyl esterase inhibitor | Fu et al., 2016 |
| orange1.1g004702m | *Citrus x sinensis* | -1.1 | Ptrif.0009s2586.1 | FS-2015-t9a |  | wall-associated kinase 2 | Fu et al., 2016 |

Note: *: based on reciprocal best hit and OrthoMCL. **: positively selected only in Poncirus trifoliata lineage among 10 species.

**Table S16.** NBS genes located within quantitative trait loci associated with Huanglongbing tolerance and *Ctv* and *Tyr1* regions.

| Gene ID | Located within |
| --- | --- |
| Ptrif.0006s1501.1 | FS-2016-t6, FS-2016-t6b (HLB) |
| Ptrif.0009s1449.1 | FS-2015-t9a (HLB) |
| Ptrif.0009s1451.1 | FS-2015-t9a (HLB) |
| Ptrif.0009s1453.1 | FS-2015-t9a (HLB) |
| Ptrif.0009s1458.2 | FS-2015-t9a (HLB) |
| Ptrif.0009s1595.1 | FS-2015-t9a (HLB) |
| Ptrif.0009s1596.1 | FS-2015-t9a (HLB) |
| Ptrif.0009s1599.1 | FS-2015-t9a (HLB) |
| Ptrif.0009s1600.1 | FS-2015-t9a (HLB) |
| Ptrif.0009s2550.1 | FS-2015-t9a (HLB) |
| Ptrif.0009s2650.1 | FS-2015-t9a (HLB) |
| Ptrif.0007s1586.1 | Ctv region |
| Ptrif.0007s1587.1 | Ctv region |
| Ptrif.0007s1590.1 | Ctv region |
| Ptrif.0007s1595.1 | Ctv region |
| Ptrif.0007s1378.1 | Tyr1 region |
| Ptrif.0007s1394.1 | Tyr1 region |
| Ptrif.0007s1395.1 | Tyr1 region |
| Ptrif.0007s1396.1 | Tyr1 region |
| Ptrif.0007s1398.1 | Tyr1 region |
| Ptrif.0007s1402.1 | Tyr1 region |
| Ptrif.0007s1404.1 | Tyr1 region |
| Ptrif.0007s1406.2 | Tyr1 region |
| Ptrif.0007s1411.2 | Tyr1 region |
| Ptrif.0007s1415.1 | Tyr1 region |
| Ptrif.0007s1481.1 | Tyr1 region |
| Ptrif.0007s1484.1 | Tyr1 region |
| Ptrif.0007s1501.1 | Tyr1 region |
| Ptrif.0007s1502.1 | Tyr1 region |
| Ptrif.0007s2703.1 | Tyr1 region |
